# Supplementary material for: Individualized prescriptive inference in ischaemic stroke
Source: Nat Commun. 2025 Oct 16;16:8968. doi: 10.1038/s41467-025-64593-7 (PMC12531334; doi:10.1038/s41467-025-64593-7)
Supplement: Supplementary file 1 — Supplementary Information [file 41467_2025_64593_MOESM1_ESM.pdf]

Supplementary Material

Figure	Title	Page
1-2	Functional parcellation visualization	1
3	Ordered list of neurological terms for each functional network	3
4	CONSORT 2010 flow diagram of dataset curation	4
5-6	Lesion archetypes	5
7-8	Disconnectome archetypes	7
9	Lesion volume distribution	10
10	Patient age and sex distributions	11
11-42	Neurotransmitter receptome subnetworks	13
43-74	Transcriptome subnetworks	45
75-76	Lesion representations & location bias results	78
77-78	Disconnectome representations & location bias results	83
79-80	Lesion representations & unobservable bias results	88
81-82	Disconnectome representations & unobservable bias results	93
83	Prescriptive analysis summary by PEHE	97
84	Prescriptive analysis summary, stratified by deficit, by PEHE	98
85	Glossary	100

Table	Title	Page
1	Linear separability, overlap, & sample sizes for lesion masks	9
2	Linear separability, overlap, & sample sizes for disconnectomes	9
3	Neurotransmitter receptome subnetworks	12
4	Lesion representations & location bias results	77
5	Disconnectome representations & location bias results	82
6	Lesion representations & unobservable bias results	87
7	Disconnectome representations & unobservable bias results	82
8	Performance comparison under ideal experimental conditions	99

Supplementary Figure 1: Functional parcellation surface renders

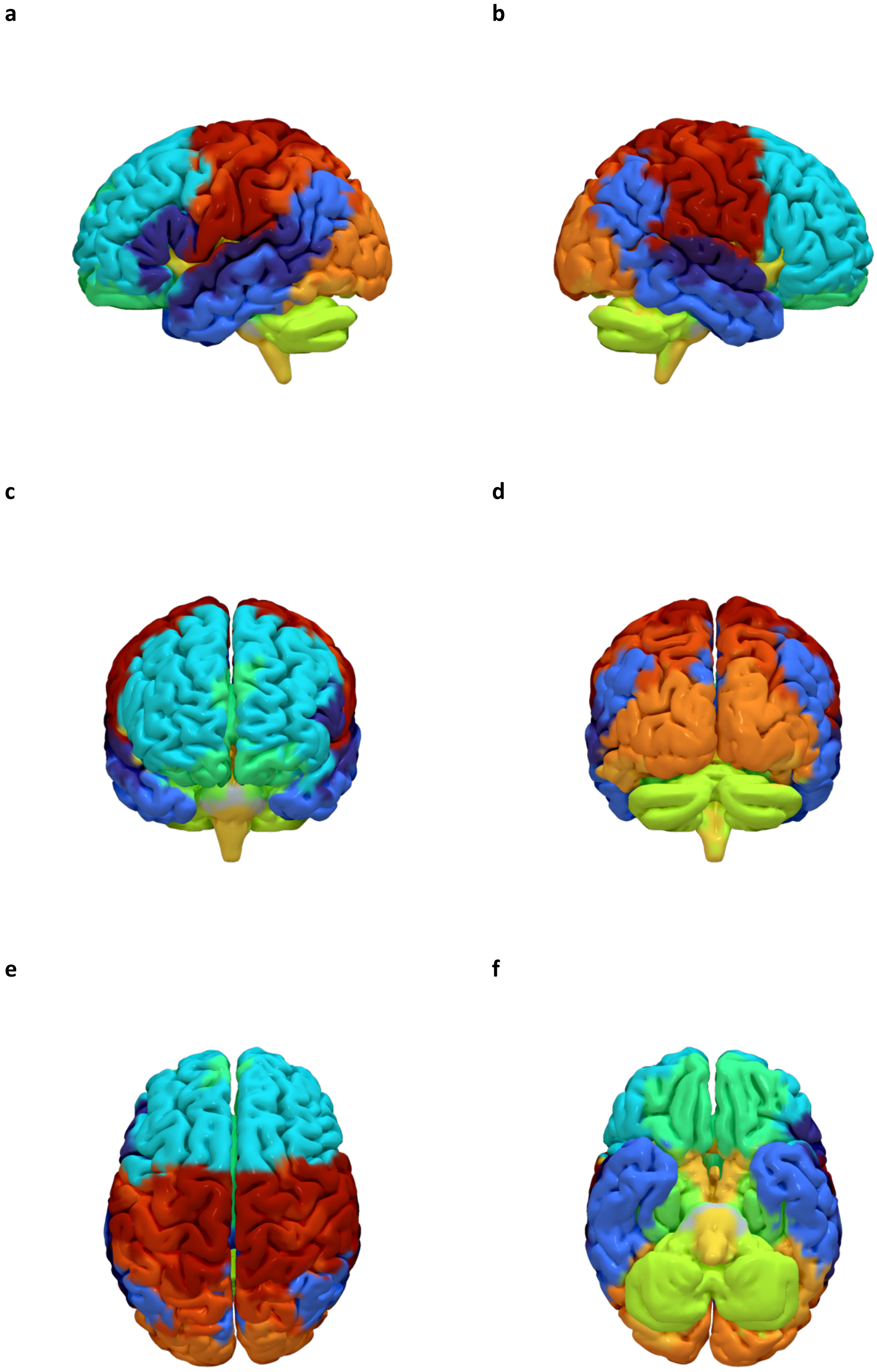

Surface rendering of the functional grey matter parcellation, with 16 distinct regions labelled by colour. The same colours are used in Figures 4, Supplementary Figure 1 and Supplementary Figure 2. The functional categories associated with each region are described in the coloured text of Figures 4, derived from the term lists in Supplementary Figure 3. Each panel shows the same surface rendering from a different spatial perspective: **a**, left; **b**, right; **c**, anterior; **d**, posterior, **e**, superior; **f**, inferior.

Supplementary Figure 2: Functional parcellation slices

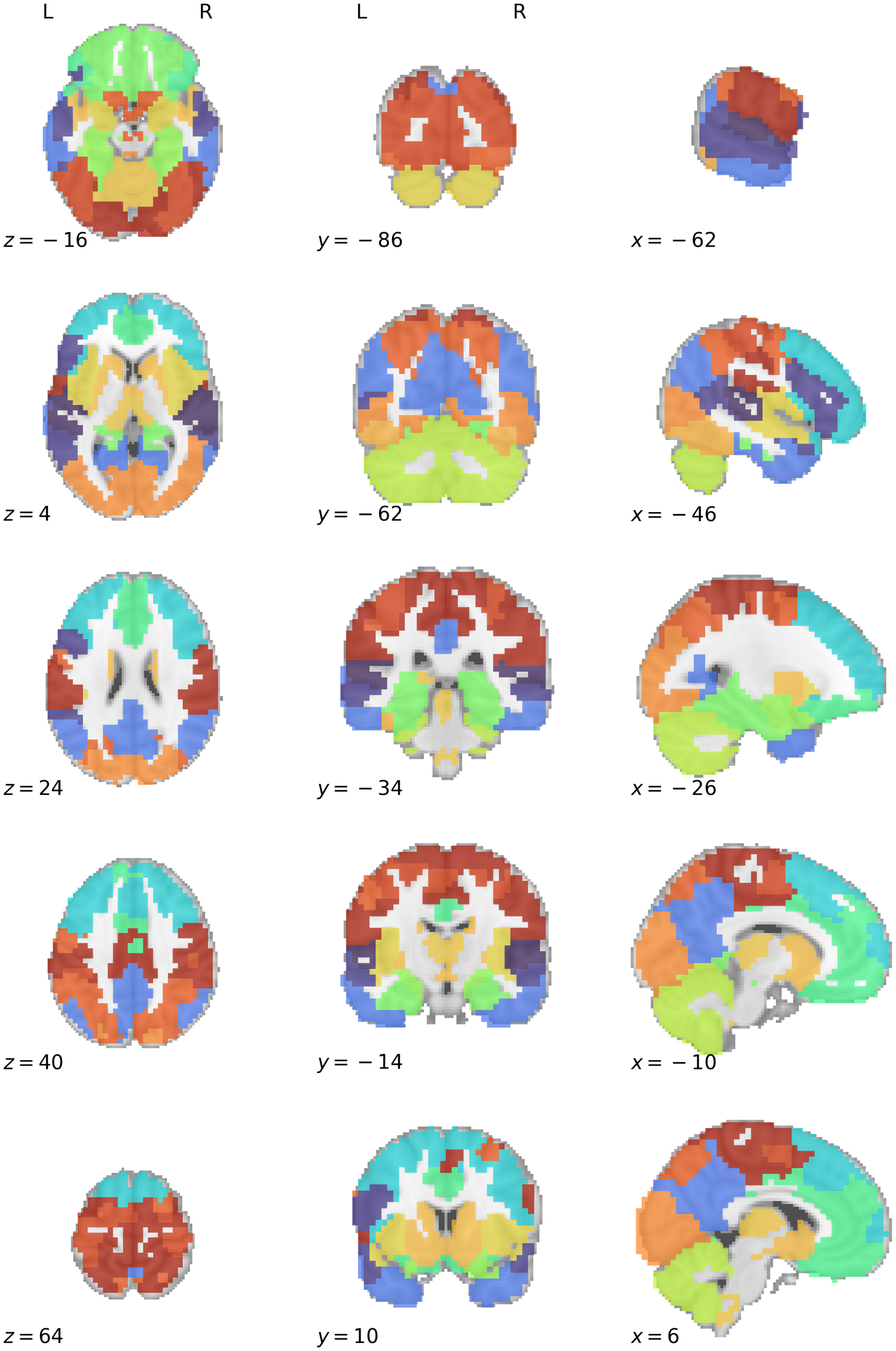

Slice visualization of the functional grey matter parcellation, with 16 distinct regions labelled by colour, overlaid onto the standard MNI152 template. The slice co-ordinates refer to MNI space. The same colours are used in Figures 4, Supplementary Figure 1 and Supplementary Figure 2. The functional categories associated with each region are described in the coloured text of Figure 4, derived from the term lists in Supplementary Figure 3.

Supplementary Figure 3: Ordered list of neurological terms for each functional network

Hearing	Lexical processing	Self-referential	Sorting
Auditory feedback	Phonological processing	Referential	Cognitive control
Acoustic	Linguistic	Mind	Handling
Auditory perception	Story	Conscious	Response execution
Acoustic processing	Spoken	Rumination	Opiate dependence
Auditory stimuli	Auditory sentence comp.	Dream	Primitive
Auditory stream segregation	Parsing	Narcisstic	Psychological stress
Vocalisation	Deafness	Self-control	Inattentional blindness
Hearing loss	Lexical	Schizotypal personality dis.	Reappraisal
Melody	Speaker	Wandering	Punish
Compulsive behaviour	Amnesia	Fearful faces	Tremor
Mood	Novelty detection	Fearful	Orthostatic
Mania	Active retrieval	Temperament	Corneal
Discipline	Spatial memory	Aggressive	Dysmetria
Unipolar depression	Spatial navigation	Emotional stimuli	Incoordination
Reversal learning	Memory loss	Uninhibited	Friedreich ataxia
Suicidability	Navigation	Psychopathic personality	Uvula
Accountability	Cognitive symptom	Neutral stimuli	Gastrocnemius
Hoarding	Encoding	Bereavement	Ataxia
Awakening	Maze	Separation anxiety	Spatial organisation
Feeling	Cataplexy	Gratification	Recognition
Viscerosensory	Slow wave sleep	Negative reinforcement	Photographs
Interoceptive	Sleep latency	Habit learning	Facial recognition
Destructive	Sleep	Habit	Visual representation
Hate	Slow wave sleep	Physical anhedonia	Prejudice
Shyness	REM sleep	Love	Selective
Tachycardia	Sleep disordered breathing	Substance dependance	Agnosia
Risk processing	Memory acquisition	Anhedonia	Viewing
Gustation	Circadian rhythm	Erotic	Repetition suppression
Disgust	Tiredness	Instrumental conditioning	Recognised
Blindness	Calculation	Flexor	Sensory perception
Visual perception	Mathematical	Extremity	Extremity
Sighted	Visuospatial	Extensor	Count
Blind	Mental arithmetic	Finger movements	Primary somatosensory
Visual impairment	Procedural knowledge	Voluntary	Muscle
Visual angle	Acalculia	Paresis	Kinaesthetic
Blindsight	Cue validity	Execution	Plantar
Ambylopia	Capacity limitation	Extension	Proprioceptive
Form perception	Arithmetic processing	Dressing	Embodied
Stereoscopic	Arithmetic	Isometric	Reflex

Ordered lists of the 10 commonest functional labels associated with each grey matter network in the parcellation (as visualized in Figures 4 and Supplementary Figures 1 and 2) by dot product with the original meta-analytic statistical maps from NeuroQuery. The colours are unified with Figure 4 and Supplementary Figures 1 and 2, with the archetypal functional categories manually identified from the above to be: (upper row, left to right) hearing, language, introspection, cognition; (second row, left to right) mood, memory, aversion, co-ordination; (third row, left to right) interoception, sleep, reward, visual recognition and (lower row, left to right) visual perception, spatial reasoning, motor, somatosensory.

Supplementary Figure 4: CONSORT 2010 lesion dataset flow chart

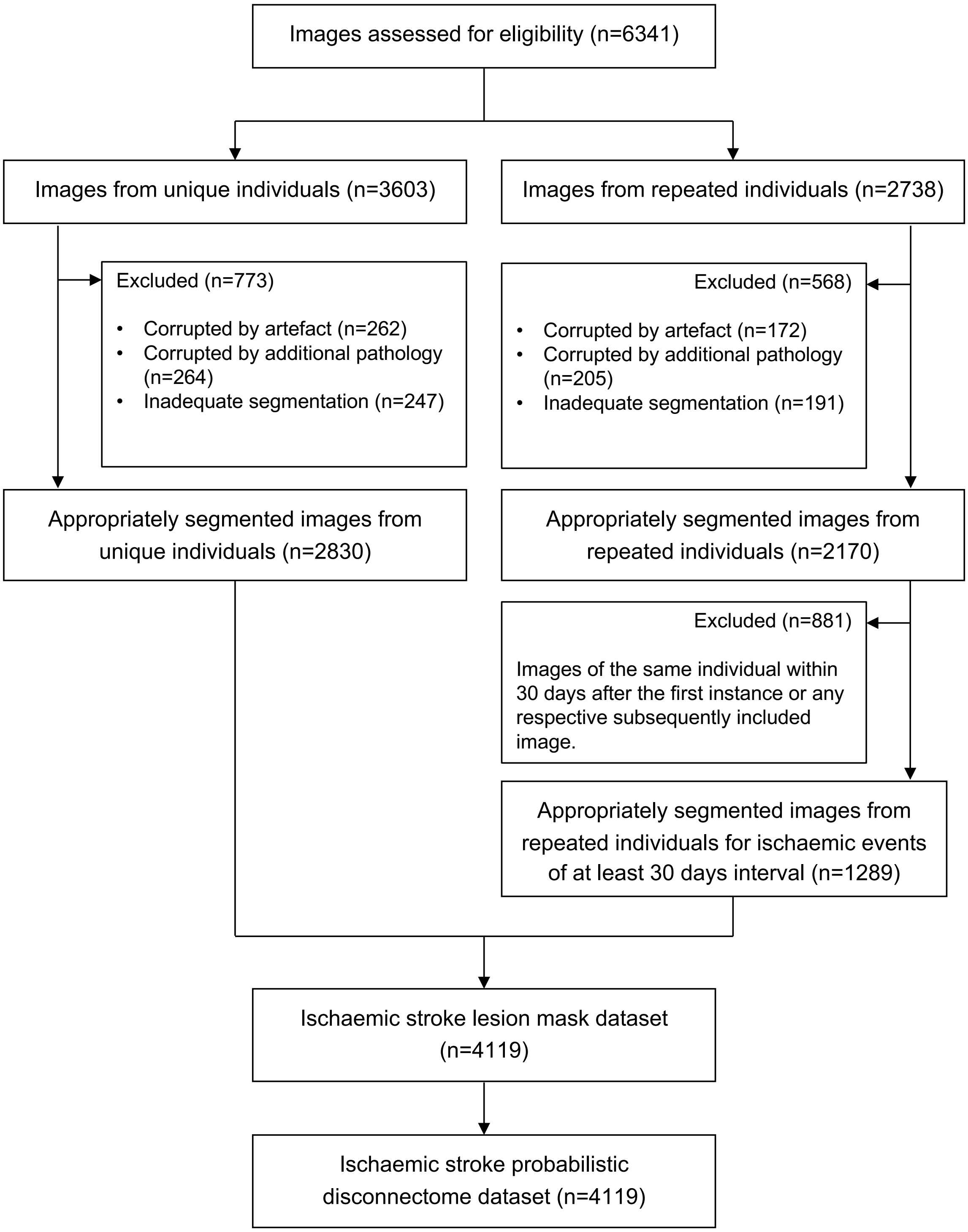

Supplementary Figure 5: Lesion NMF archetypes

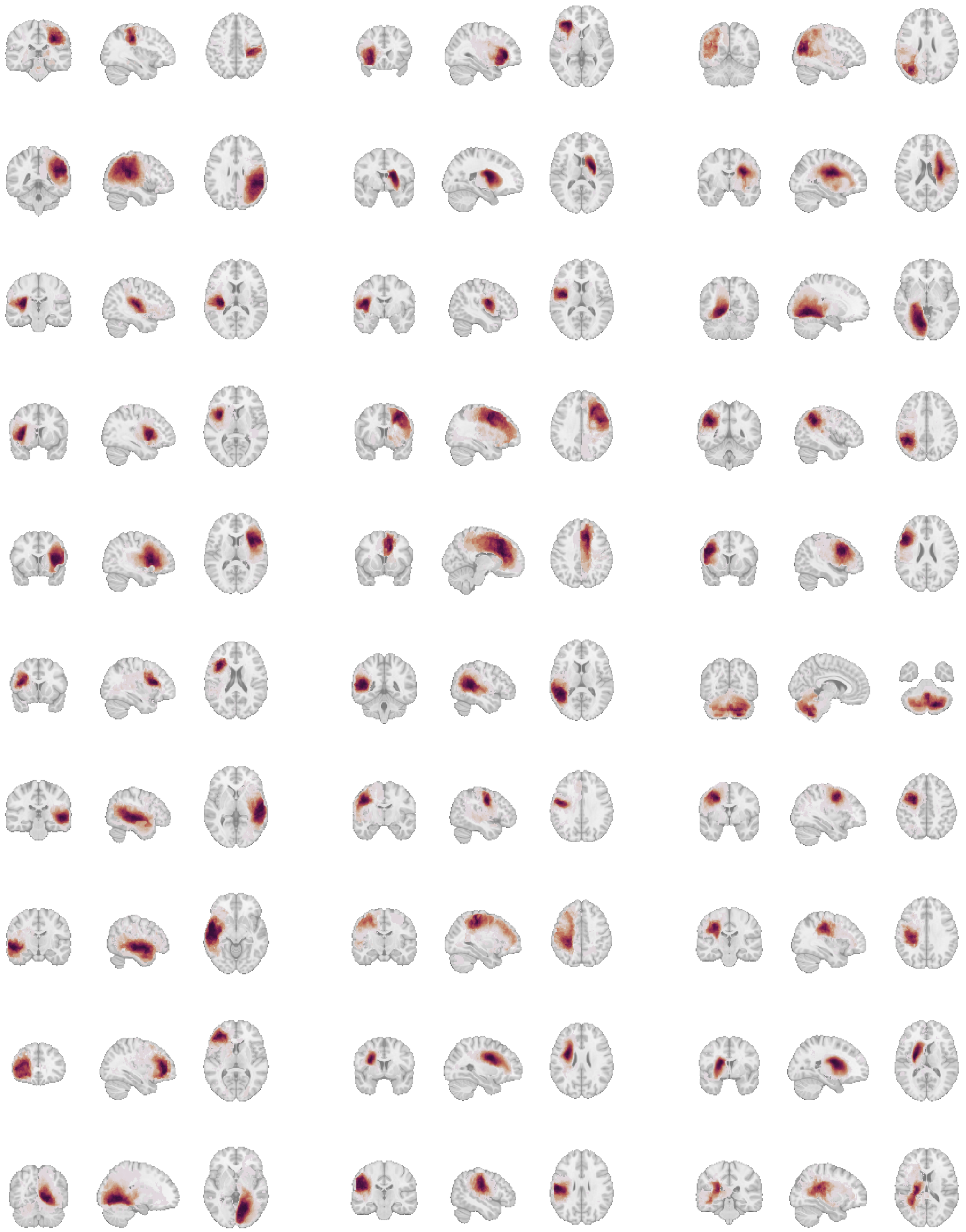

Spatial reconstructions of the latent factors from a 30-dimensional non-negative matrix factorization of the lesion masks. The spatial distribution of each reconstruction is visualized. Coronal (left), sagittal (centre) and axial (right) slice are shown for each, with co-ordinates set to the location of maximum density.

Supplementary Figure 6: Lesion PCA archetypes

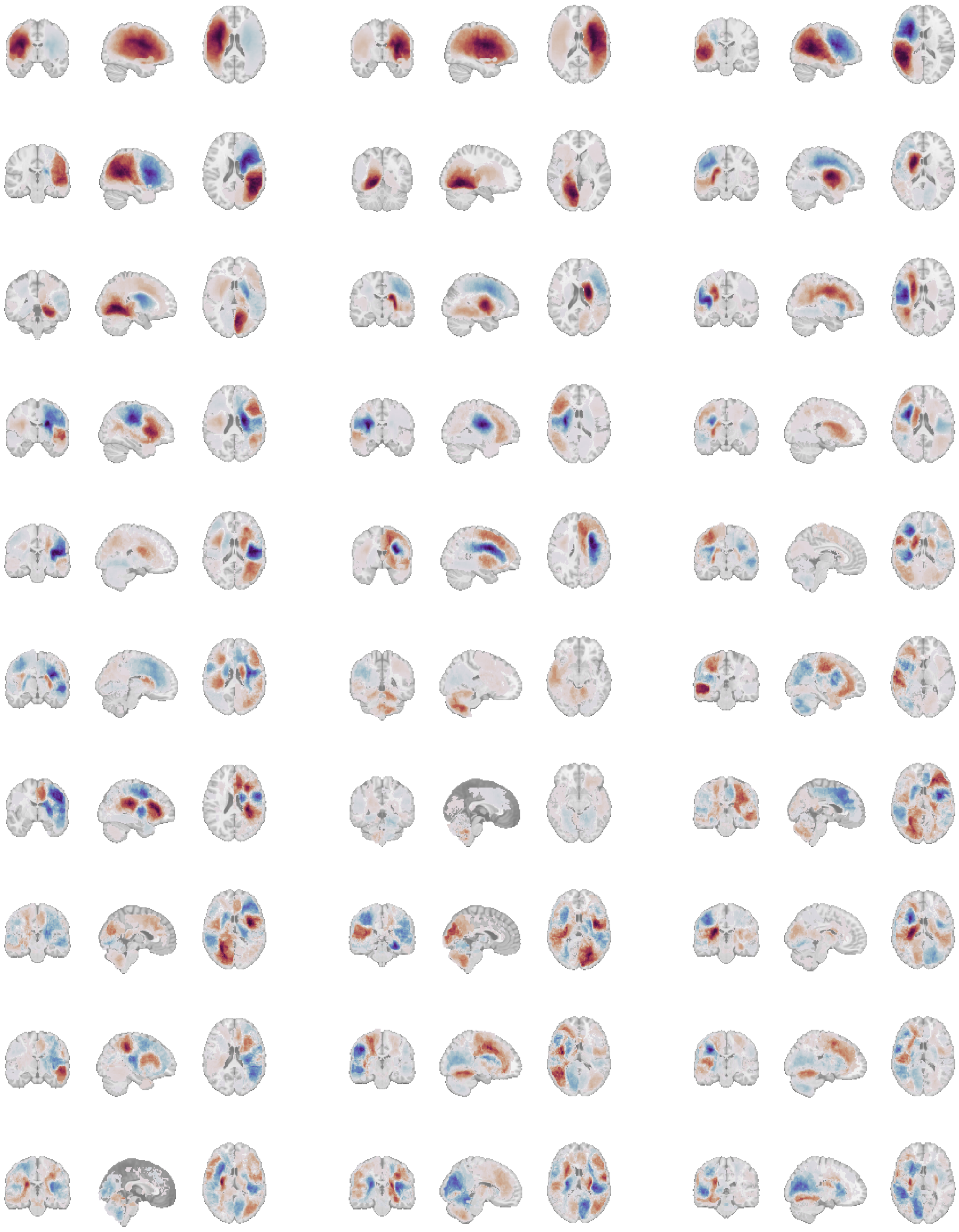

Spatial reconstructions of the latent factors from a 30-dimensional principal component analysis-derived representation of the lesion masks. The spatial distribution of each reconstruction is visualized. Coronal (left), sagittal (centre) and axial (right) slice are shown for each, with co-ordinates set to the location of maximum density.

Supplementary Figure 7: Disconnectome NMF archetypes

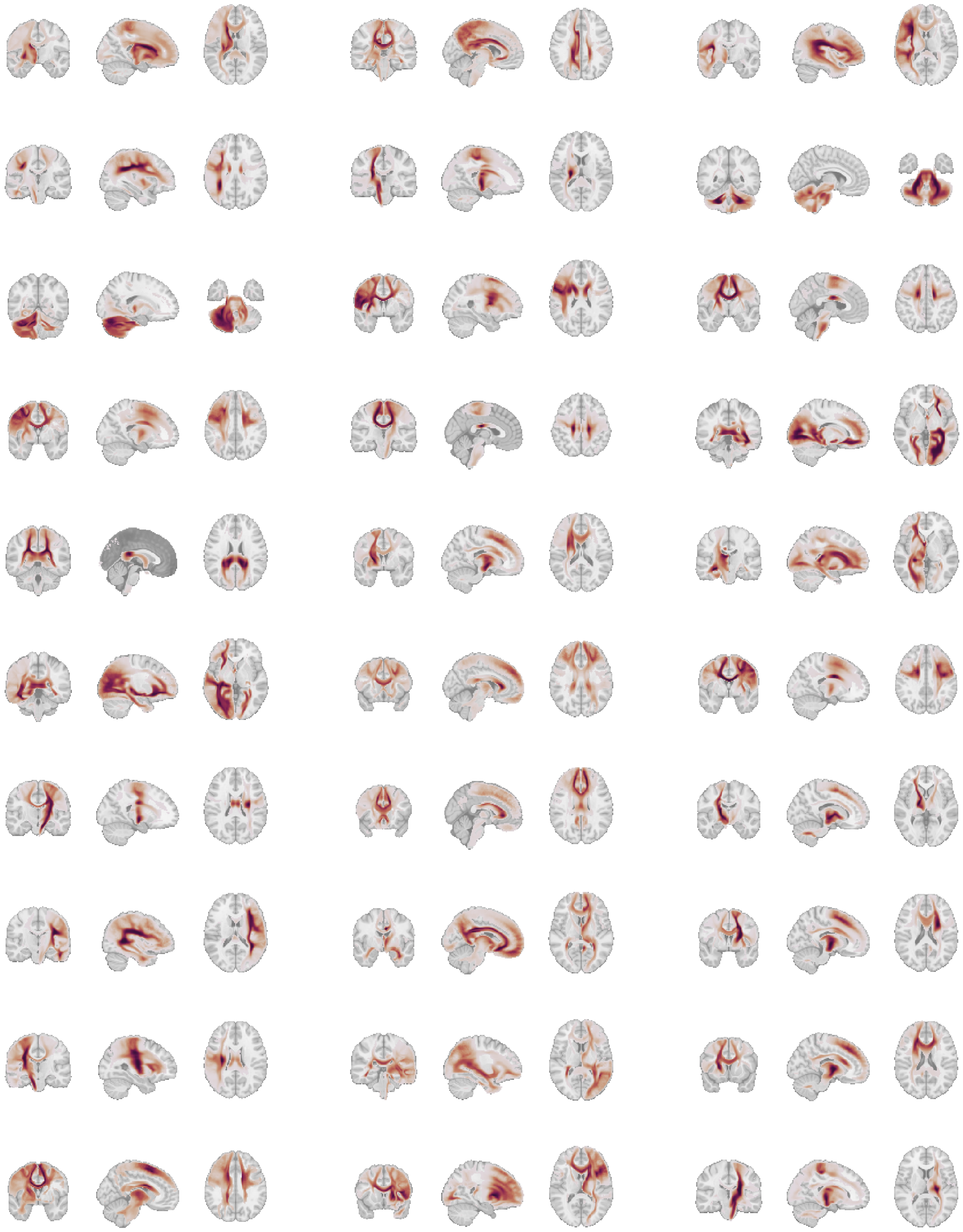

Spatial reconstructions of the latent factors from a 30-dimensional non-negative matrix factorization of the disconnectomes. The spatial distribution of each reconstruction is visualized. Coronal (left), sagittal (centre) and axial (right) slice are shown for each, with co-ordinates set to the location of maximum density.

Supplementary Figure 8: Disconnectome PCA archetypes

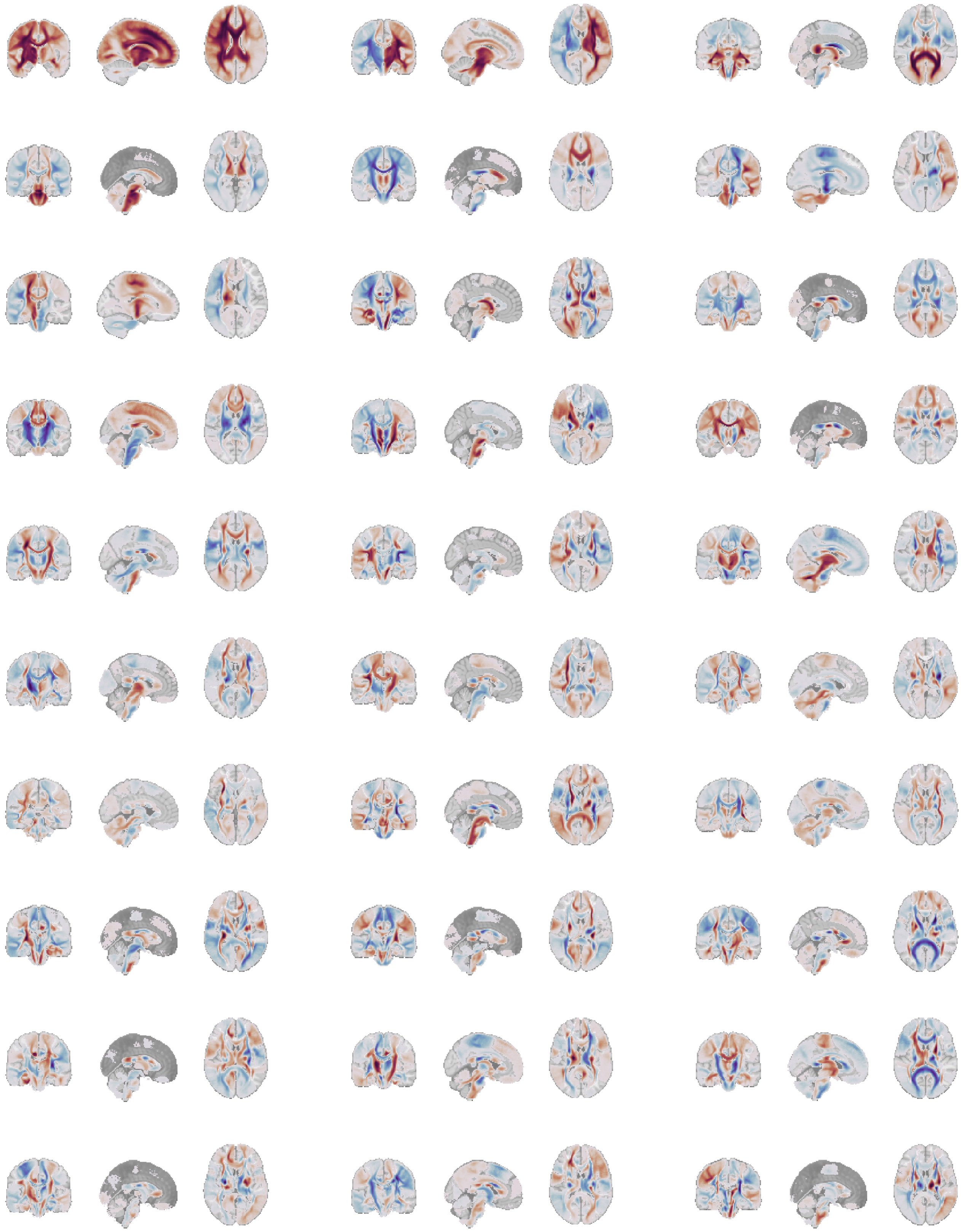

Spatial reconstructions of the latent factors from a 30-dimensional principal component analysis-derived representation of the disconnectomes. The spatial distribution of each reconstruction is visualized. Coronal (left), sagittal (centre) and axial (right) slice are shown for each, with co-ordinates set to the location of maximum density.

Supplementary Table 1: Linear separability, overlap, & sample sizes for lesion masks

Lesions, N = 4,119, threshold = 0.05

	Receptome				Transcriptome			
	Linear separability		Overlap	N	Linear separability		Overlap	N
	OR	XOR			OR	XOR		
**Hearing**	0.618	0.985	0.603	305	0.684	0.949	0.506	322
**Language**	0.567	NaN	0.463	240	0.991	0.991	0	264
**Introspection**	0.799	0.857	0.309	94	0.739	0.908	0.291	103
**Cognition**	0.546	0.500	0.321	196	0.653	0.919	0.548	168
**Mood**	0.593	0.667	0.516	31	0.938	1	0.194	31
**Memory**	0.580	0.643	0.779	136	0.593	0.954	0.650	157
**Aversion**	0.603	0.686	0.341	88	0.695	0.780	0.344	96
**Coordination**	0.816	0.950	0.413	109	0.648	0.883	0.543	105
**Interoception**	0.623	0.938	0.669	495	0.577	0.903	0.665	487
**Sleep**	0.702	0.938	0.337	83	0.722	0.908	0.372	102
**Reward**	0.694	0.900	0.355	304	0.550	0.667	0.039	356
**Visual recognition**	0.876	1	0.151	86	0.784	0.895	0.190	84
**Visual perception**	0.762	0.772	0.318	154	0.655	0.950	0.506	176
**Spatial reasoning**	0.658	0.925	0.594	207	0.819	0.955	0.339	239
**Motor**	0.737	0.940	0.403	278	0.993	0.993	0.006	324
**Somatosensory**	0.693	0.902	0.464	267	0.981	0.992	0.009	335

Separability, overlap and sample size characteristics of the virtual cohorts defined by functional subgroup and physiology type for lesion models. The *linear separability* column lists the balanced accuracy of a linear support vector machine trained to distinguish between “affected” and “unaffected” members of each cohort as defined by the intersection of the functional and physiological labels at a threshold of 0.05; the *overlap* column lists the proportion of lesions that exceed the threshold for both hypothetical treatments; and the *N* column lists the total number of patients from the dataset with lesions meeting the threshold for at least one subnetwork.

Supplementary Table 2: Linear separability, overlap, & sample sizes for disconnectomes

Disconnectomes, N = 4,119, threshold = 0.05, *p* > 0.5

	Receptome				Transcriptome			
	Linear separability		Overlap	N	Linear separability		Overlap	N
	OR	XOR			OR	XOR		
**Hearing**	0.645	0.987	0.614	868	0.704	0.986	0.512	887
**Language**	0.593	0.500	0.660	688	0.989	0.998	0.009	851
**Introspection**	0.666	0.990	0.545	508	0.846	0.990	0.223	637
**Cognition**	0.574	0.800	0.573	853	0.580	0.982	0.743	829
**Mood**	0.605	0.988	0.604	364	0.645	0.949	0.588	381
**Memory**	0.617	NaN	0.409	978	0.566	0.958	0.708	955
**Aversion**	0.608	0.769	0.645	1503	0.556	0.953	0.800	1346
**Coordination**	0.529	0.772	0.698	325	0.607	0.943	0.616	378
**Interoception**	0.554	0.903	0.761	1806	0.583	0.823	0.675	1872
**Sleep**	0.652	0.939	0.553	3078	0.616	0.993	0.630	2781
**Reward**	0.629	0.974	0.658	1854	0.660	0.972	0.550	1993
**Visual recognition**	0.848	0.988	0.167	335	0.838	0.963	0.215	297
**Visual perception**	0.626	0.843	0.469	407	0.577	0.900	0.234	376
**Spatial reasoning**	0.662	0.972	0.408	1011	0.714	0.951	0.407	863
**Motor**	0.697	0.970	0.461	1230	0.903	0.998	0.157	1394
**Somatosensory**	0.700	0.966	0.452	1202	0.905	0.999	0.158	1427

Separability, overlap and sample size characteristics of the virtual cohorts defined by functional subgroup and physiology type for disconnectome models, where the disconnectome is binarized at a disconnection threshold of 0.5. The *linear separability* column lists the balanced accuracy of a linear support vector machine trained to distinguish between “affected” and “unaffected” members of each cohort as defined by the intersection of the functional and physiological labels at a threshold of 0.05; the *overlap* column lists the proportion of lesions that exceed the threshold for both hypothetical treatments; and the *N* column lists the total number of patients from the dataset with lesions meeting the threshold for at least one subnetwork.

Supplementary Figure 9: Lesion volume distribution

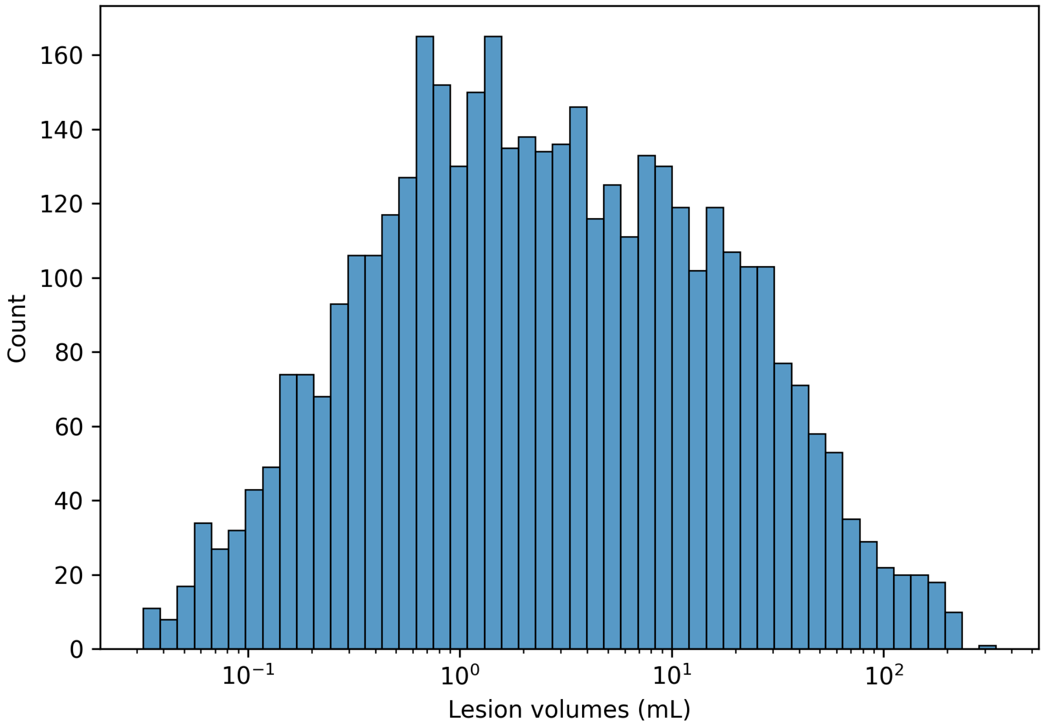

a. Histogram showing the distribution of lesion volumes for the dataset of 4,119 events, after non-linear registration to MNI space. The mean is 11.68 mL, with standard deviation of 25.49 mL. Note decimal log scale.

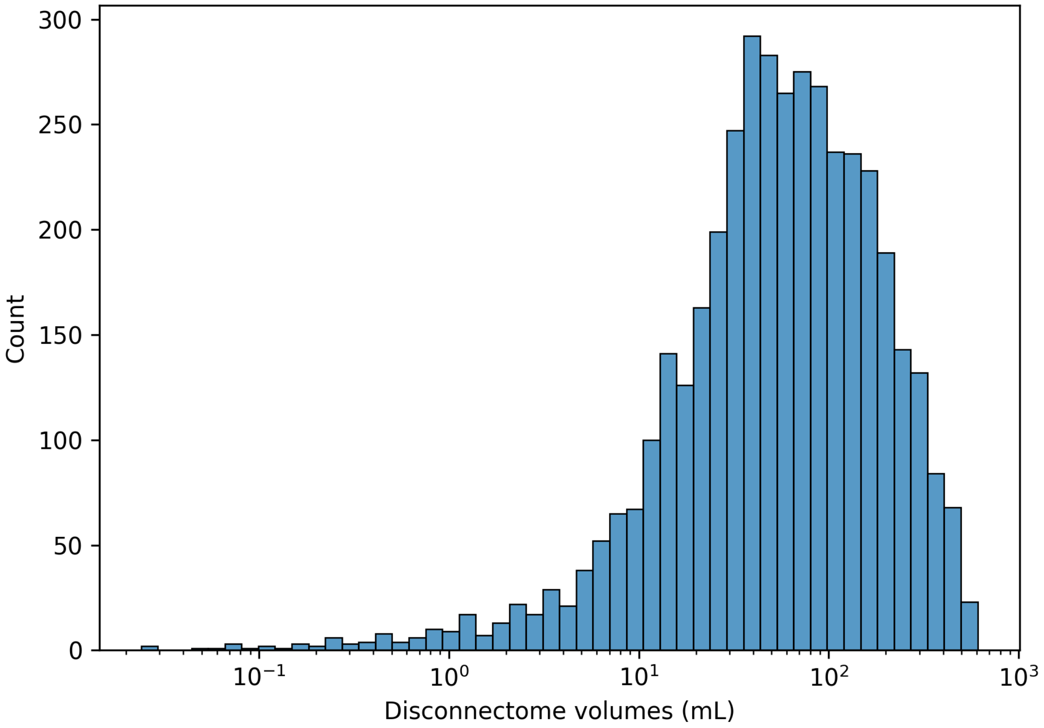

b. Histogram showing the distribution of binarized (p > 0.5) disconnectome volumes for the dataset of 4,119 events, after non-linear registration to MNI space. The mean is 94.26 mL, with standard deviation of 101.04 mL. Note decimal log scale.

Supplementary Figure 10: Patient age distribution

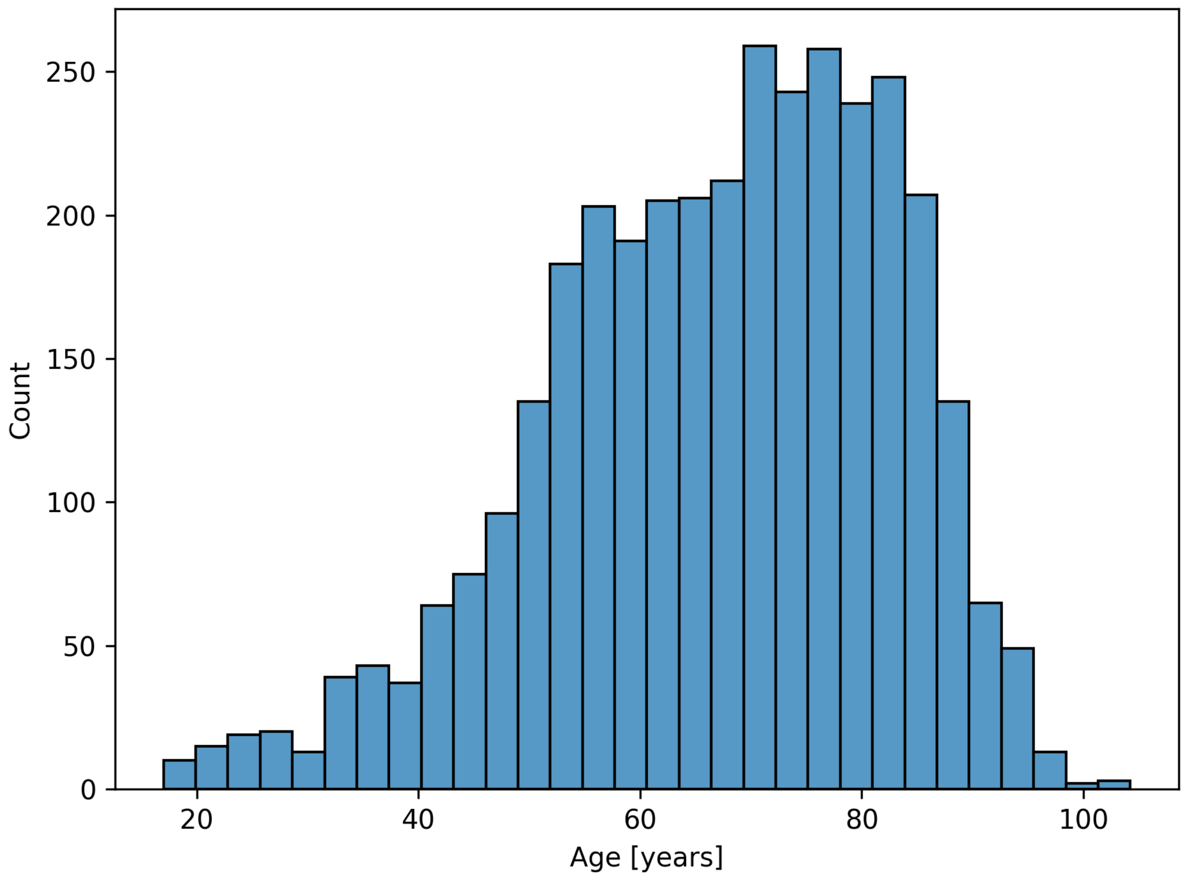

Histogram showing the distribution of patient age. Age data was available for 3487/4119 images and exhibited a mean of 67.042 years and standard deviation 15.46 years. From 3478/4119 images with complete data on patient sex, 1,960 were recorded as male (56.4%) and 1,518 as female (43.6%).

Supplementary Table 3: Neurotransmitter receptome subnetworks

Functional Deficit	Primary receptor type	Secondary receptor type
**Hearing**	Noradrenaline	Glutamate
**Language**	Cannabinoid	Glutamate
**Introspection**	Glutamate	5HT
**Cognition**	Cannabinoid	Opioid
**Mood**	Opioid	Histamine
**Memory**	Dopamine	5HT
**Aversion**	Dopamine	Histamine
**Coordination**	Opioid	Acetylcholine
**Interoception**	Dopamine	Histamine
**Sleep**	Opioid	Noradrenaline
**Reward**	Dopamine	Histamine
**Visual recognition**	Cannabinoid	GABA
**Visual perception**	GABA	5HT
**Spatial reasoning**	Glutamate	Noradrenaline
**Motor**	Noradrenaline	Glutamate
**Somatosensory**	Noradrenaline	Glutamate

The primary and secondary receptor classes by preponderance within each of the functional networks, according to aggregation of Z-valued transformations of positron emission tomography data, each targeted to receptor subclasses. The preponderance was determined by the proportions of voxels from each functional network whose receptor type aggregated Z-value was maximal (primary) and second-maximal (secondary) over all others. The following figures show, for each functional network, the functional network subdivisions based upon neurotransmitter receptor preponderance, derived from positron emission tomography data made available by Hansen et al. (2022).

Supplementary Figure 11: *Hearing* subnetwork by receptome render

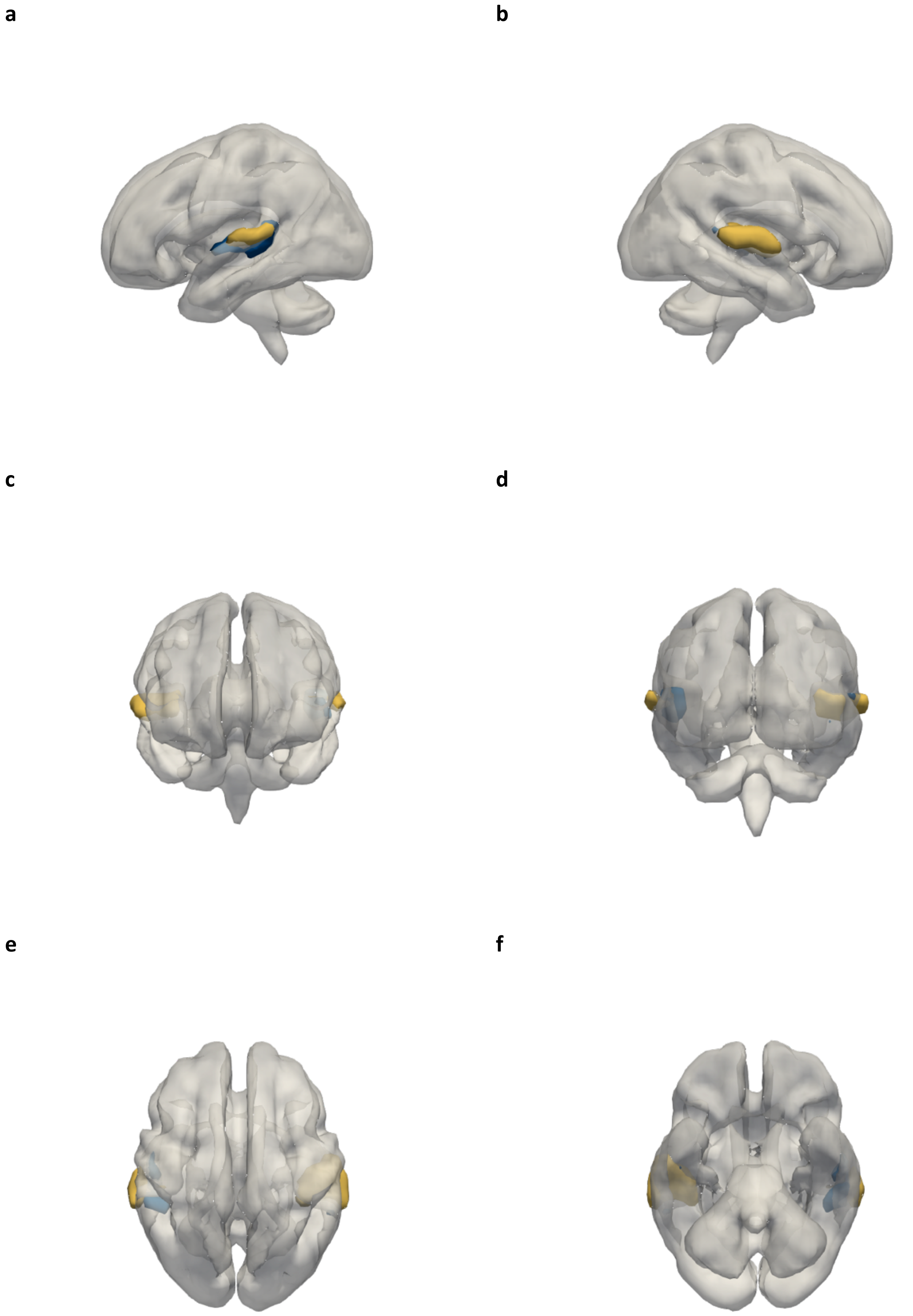

Three-dimensional rendering of the functional grey matter network representing *hearing*. The colours label functional subnetworks separated by neurotransmitter receptor distribution preponderance, here noradrenaline in yellow and glutamate in blue. This forms the basis upon which treatment effect heterogeneity is simulated, with hypothetical treatments selectively effective for lesions disrupting defined receptor territories. Each panel shows the same render from a different spatial perspective: **a**, left; **b**, right; **c**, anterior; **d**, posterior, **e**, superior; **f**, inferior. The underlay is a thresholded white matter template surface in MNI.

Supplementary Figure 12: *Hearing* subnetwork by receptome slices

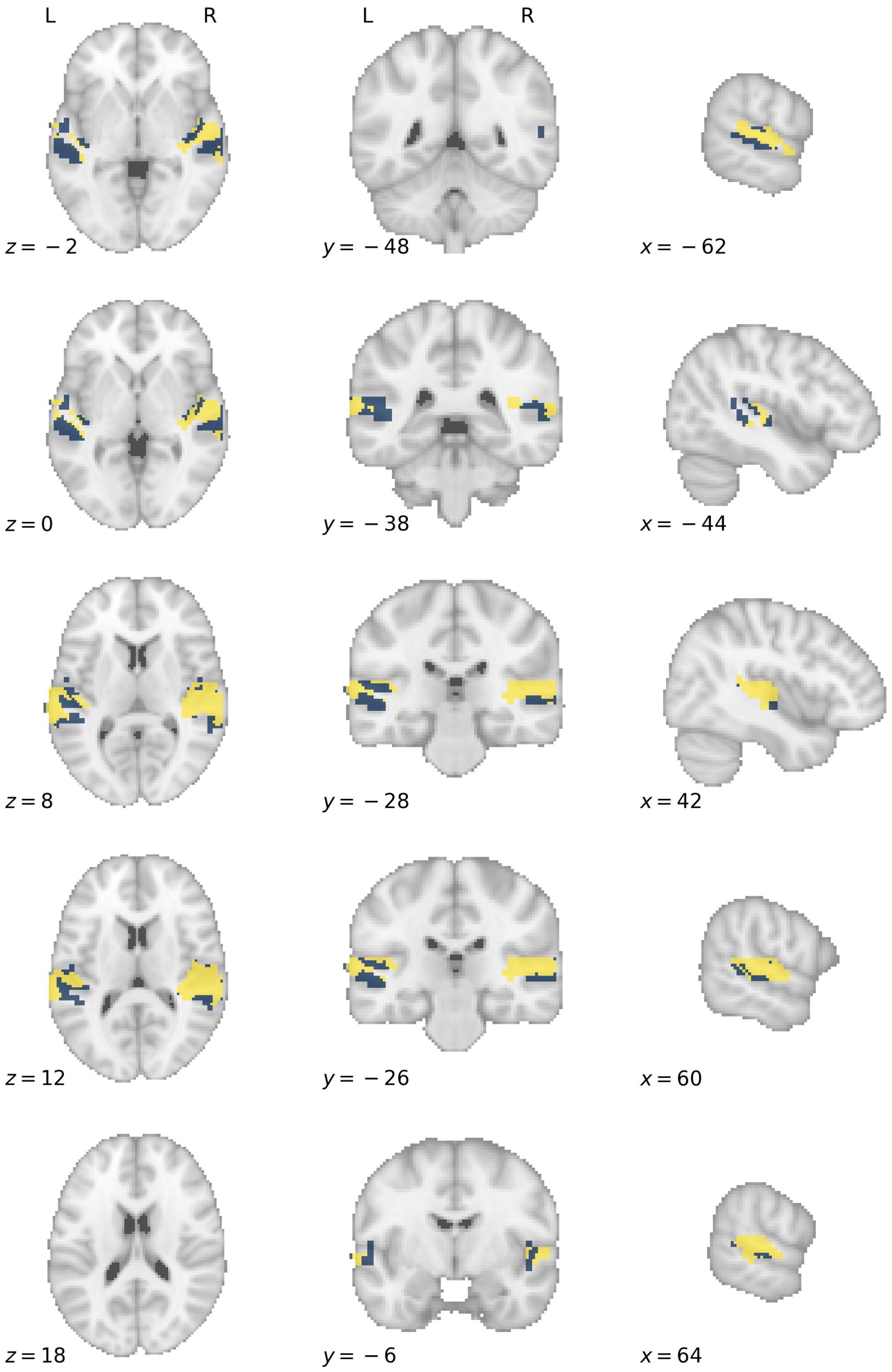

Slice visualization of the functional grey matter network representing *hearing*, overlaid onto the standard MNI152 template. The labelled co-ordinates map to MNI space. The colours label functional subnetworks separated by neurotransmitter receptor distribution preponderance, here noradrenaline in yellow and glutamate in blue. This forms the basis upon which treatment effect heterogeneity is simulated, with hypothetical treatments selectively effective for lesions disrupting defined receptor territories.

Supplementary Figure 13: *Language* subnetwork by receptome render

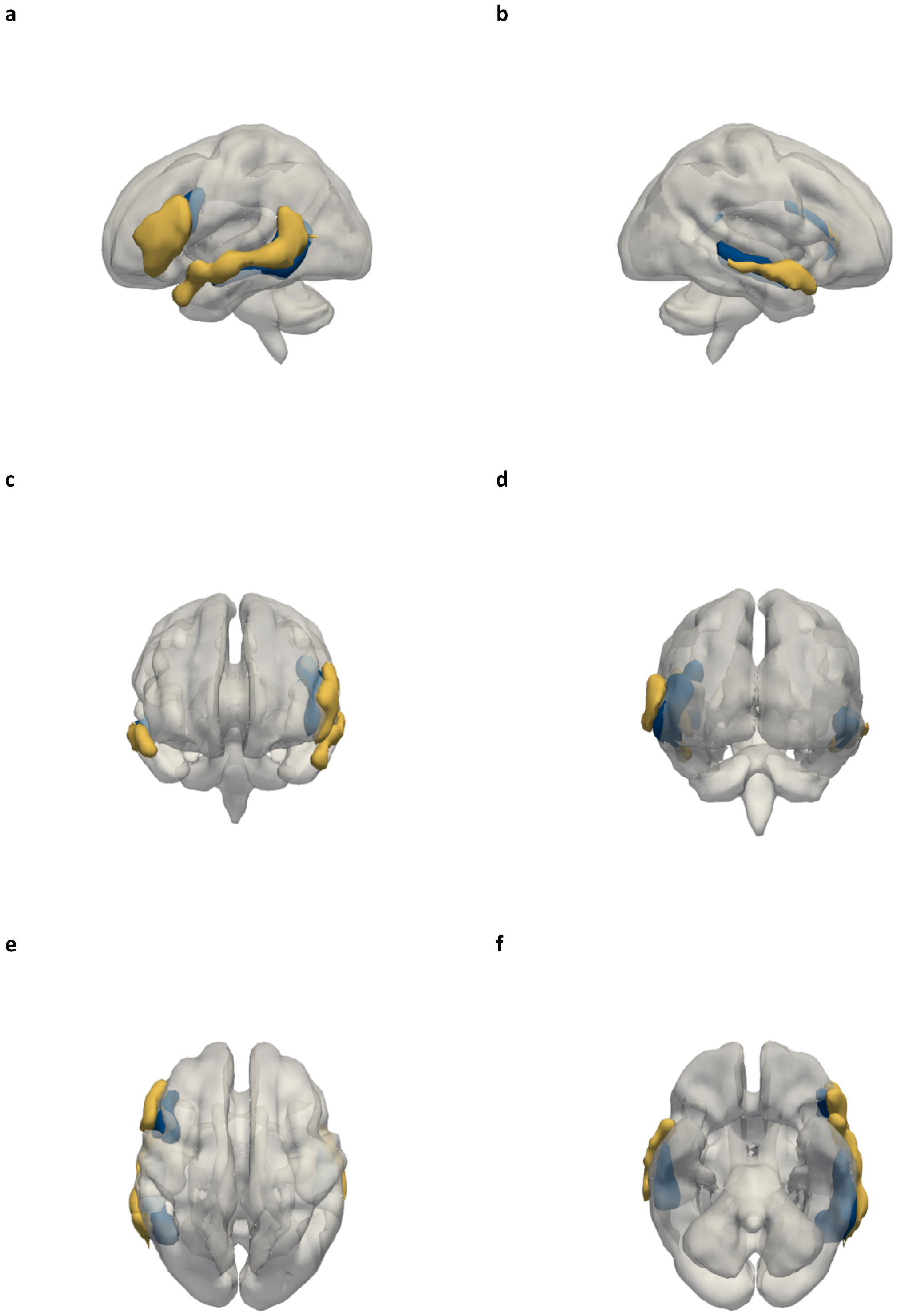

Three-dimensional rendering of the functional grey matter network representing *language*. The colours label functional subnetworks separated by neurotransmitter receptor distribution preponderance, here cannabinoid in yellow and glutamate in blue. This forms the basis upon which treatment effect heterogeneity is simulated, with hypothetical treatments selectively effective for lesions disrupting defined receptor territories. Each panel shows the same render from a different spatial perspective: **a**, left; **b**, right; **c**, anterior; **d**, posterior, **e**, superior; **f**, inferior. The underlay is a thresholded white matter template surface in MNI.

Supplementary Figure 14: *Language* subnetwork by receptome slices

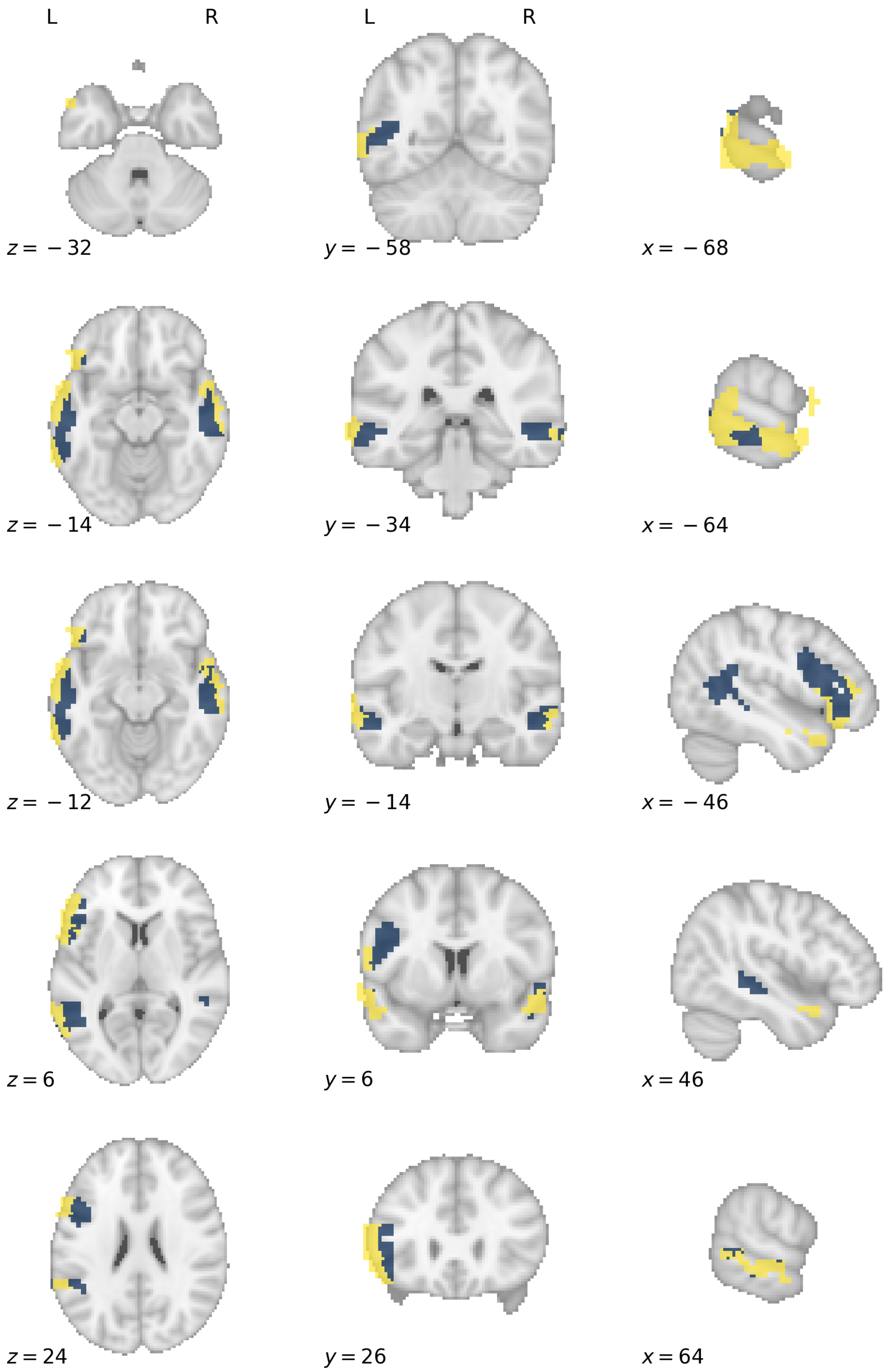

Slice visualization of the functional grey matter network representing *language*, overlaid onto the standard MNI152 template. The labelled co-ordinates map to MNI space. The colours label functional subnetworks separated by neurotransmitter receptor distribution preponderance, here cannabinoid in yellow and glutamate in blue. This forms the basis upon which treatment effect heterogeneity is simulated, with hypothetical treatments selectively effective for lesions disrupting defined receptor territories.

Supplementary Figure 15: *Introspection* subnetwork by receptome render

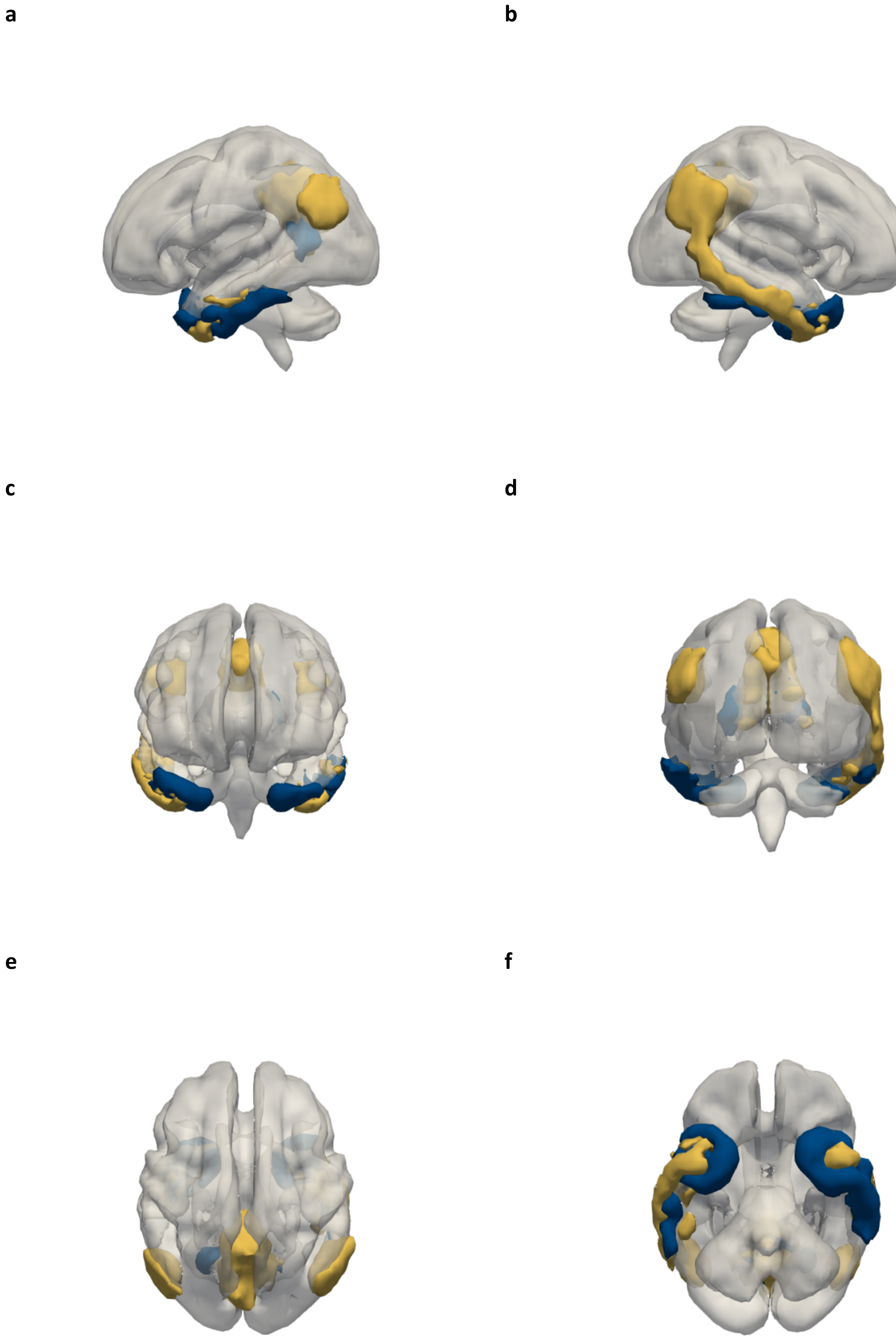

Three-dimensional rendering of the functional grey matter network representing *introspection*. The colours label functional subnetworks separated by neurotransmitter receptor distribution preponderance, here glutamate in yellow and 5HT in blue. This forms the basis upon which treatment effect heterogeneity is simulated, with hypothetical treatments selectively effective for lesions disrupting defined receptor territories. Each panel shows the same render from a different spatial perspective: **a**, left; **b**, right; **c**, anterior; **d**, posterior, **e**, superior; **f**, inferior. The underlay is a thresholded white matter template surface in MNI.

Supplementary Figure 16: *Introspection* subnetwork by receptome slices

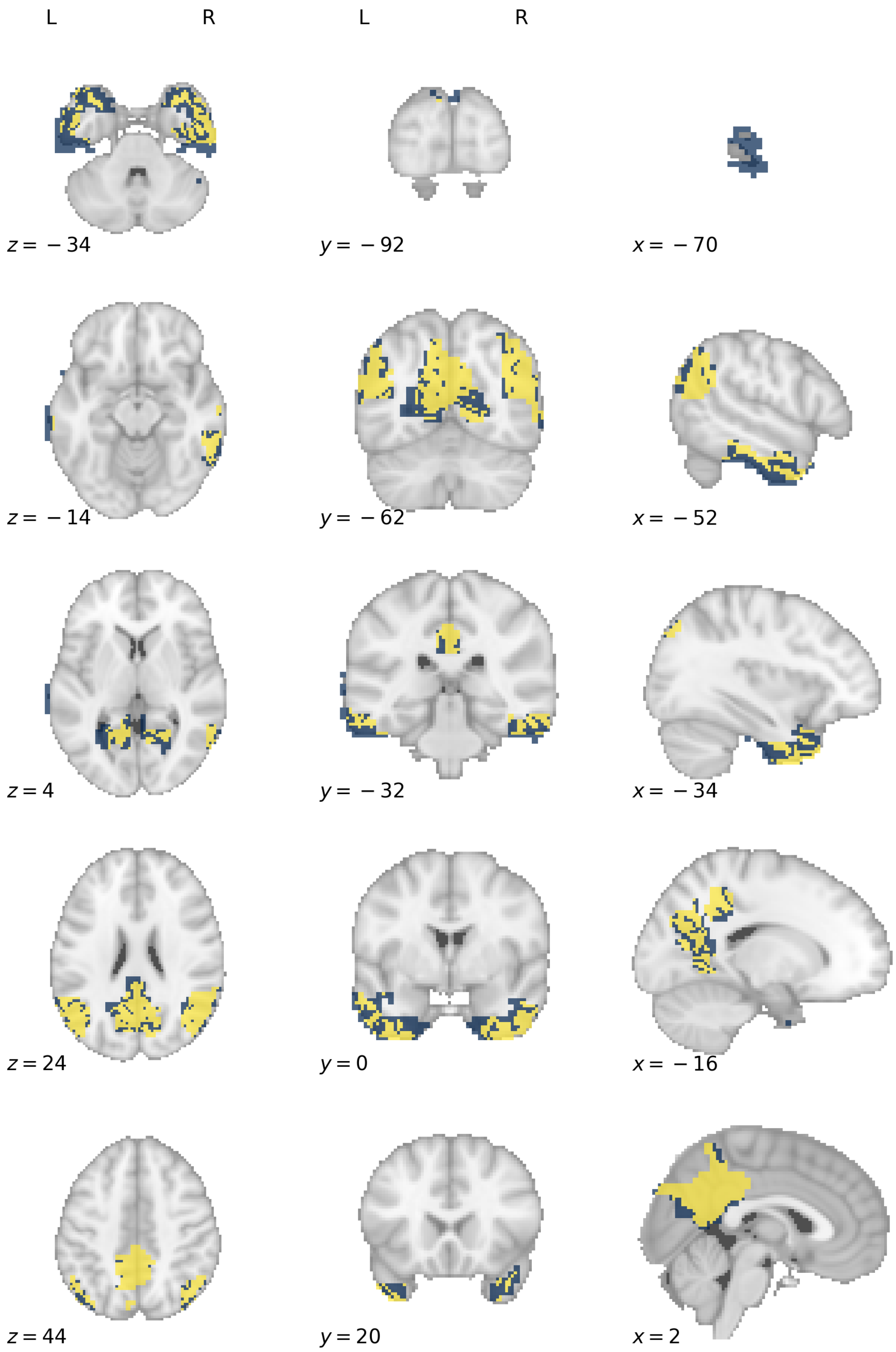

Slice visualization of the functional grey matter network representing *introspection*, overlaid onto the standard MNI152 template. The labelled co-ordinates map to MNI space. The colours label functional subnetworks separated by neurotransmitter receptor distribution preponderance, here glutamate in yellow and 5HT in blue. This forms the basis upon which treatment effect heterogeneity is simulated, with hypothetical treatments selectively effective for lesions disrupting defined receptor territories.

Supplementary Figure 17: *Cognition* subnetwork by receptome render

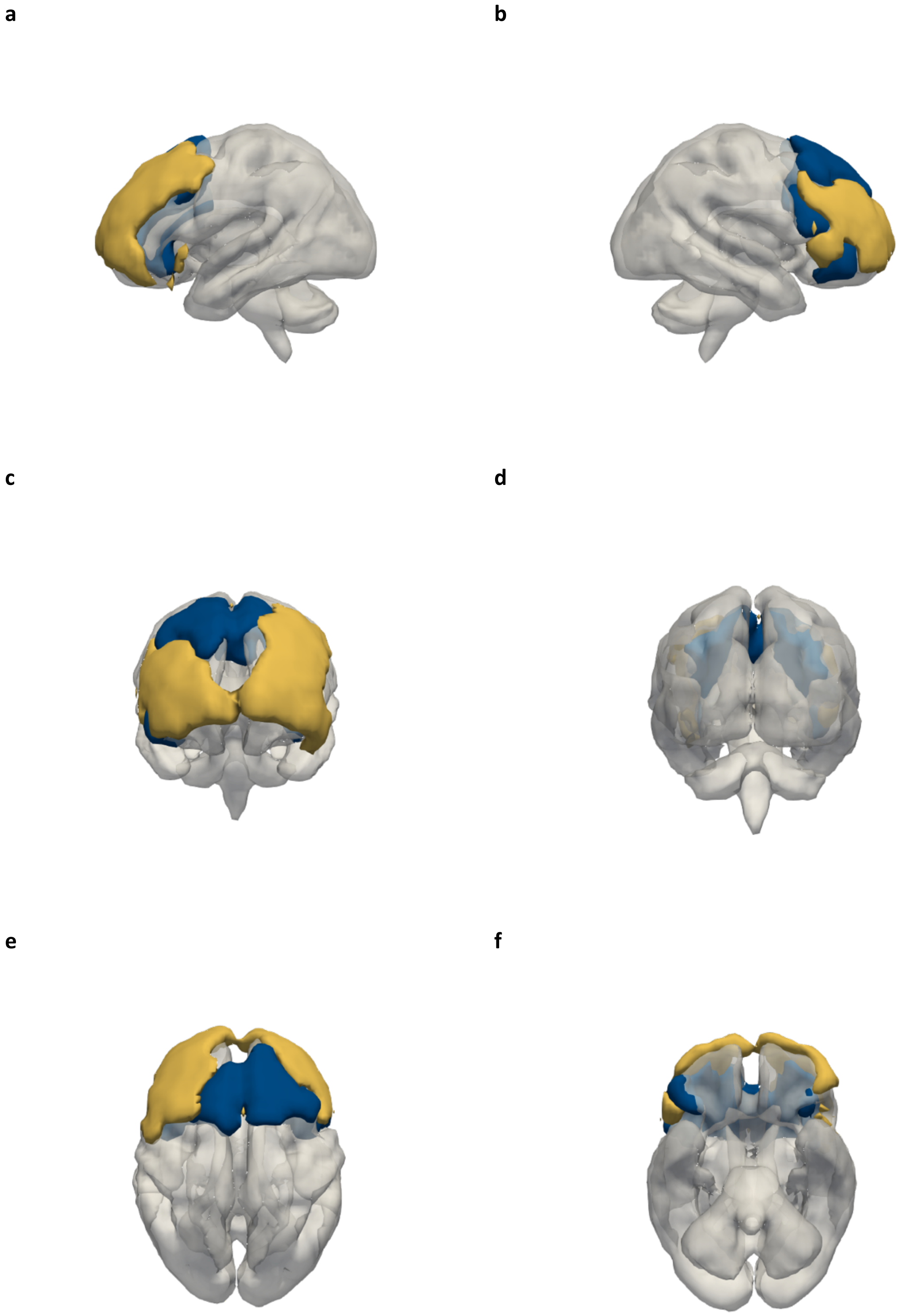

Three-dimensional rendering of the functional grey matter network representing *cognition*. The colours label functional subnetworks separated by neurotransmitter receptor distribution preponderance, here cannabinoid in yellow and opioid in blue. This forms the basis upon which treatment effect heterogeneity is simulated, with hypothetical treatments selectively effective for lesions disrupting defined receptor territories. Each panel shows the same render from a different spatial perspective: **a**, left; **b**, right; **c**, anterior; **d**, posterior, **e**, superior; **f**, inferior. The underlay is a thresholded white matter template surface in MNI.

Supplementary Figure 18: *Cognition* subnetwork by receptome slices

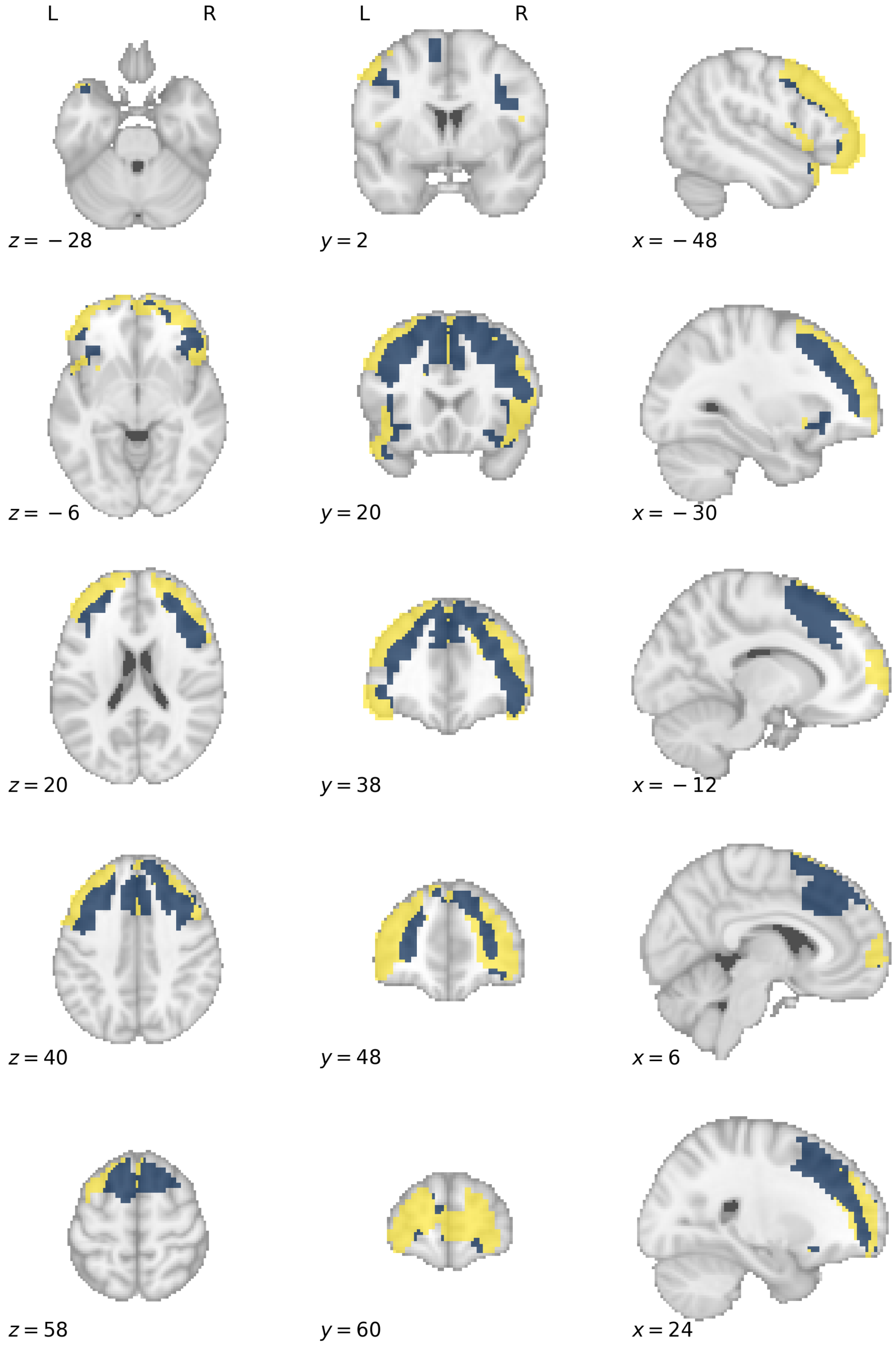

Slice visualization of the functional grey matter network representing *cognition*, overlaid onto the standard MNI152 template. The labelled co-ordinates map to MNI space. The colours label functional subnetworks separated by neurotransmitter receptor distribution preponderance, here cannabinoid in yellow and opioid in blue. This forms the basis upon which treatment effect heterogeneity is simulated, with hypothetical treatments selectively effective for lesions disrupting defined receptor territories.

Supplementary Figure 19: *Mood* subnetwork by receptome render

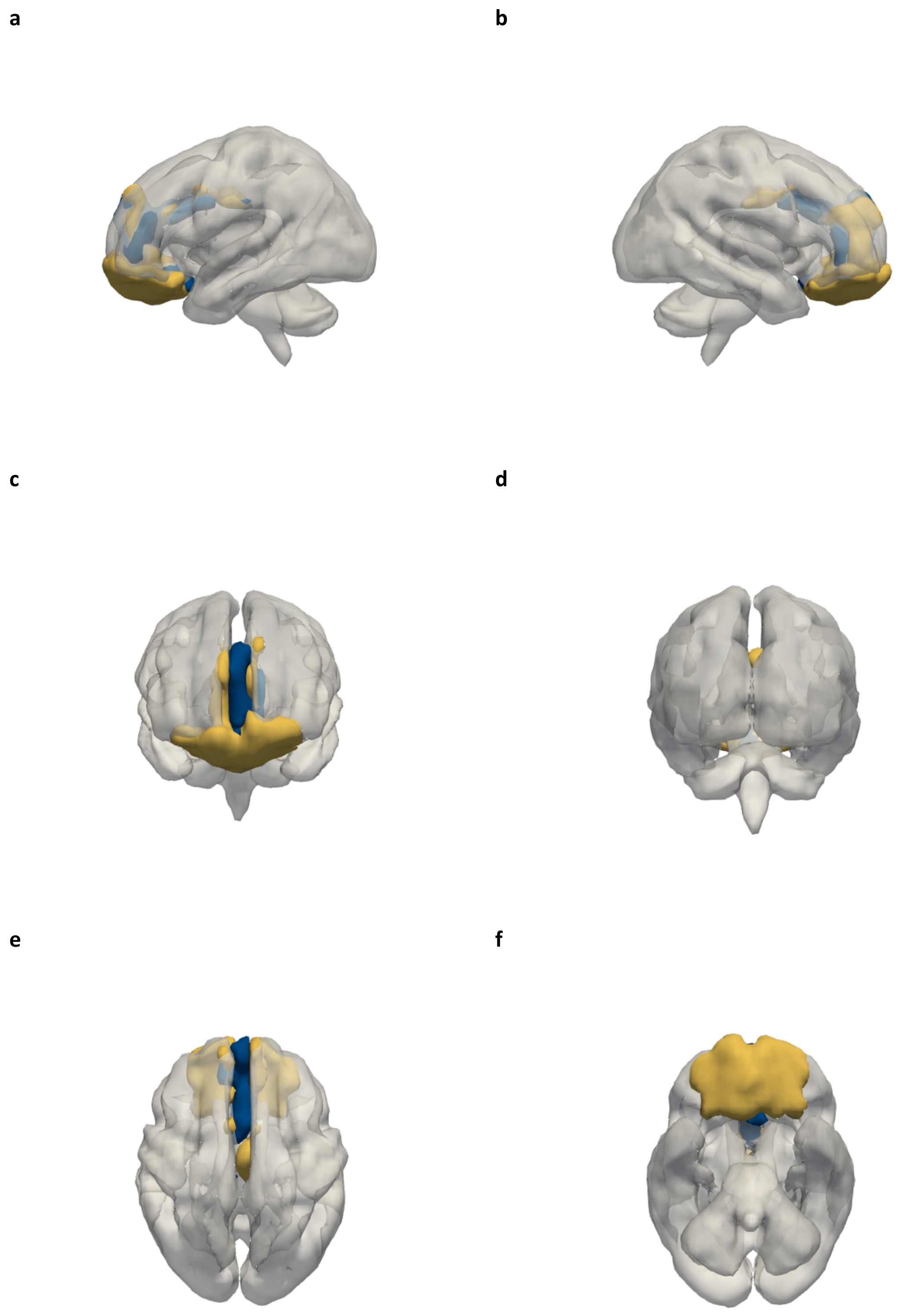

Three-dimensional rendering of the functional grey matter network representing *mood*. The colours label functional subnetworks separated by neurotransmitter receptor distribution preponderance, here opioid in yellow and histamine in blue. This forms the basis upon which treatment effect heterogeneity is simulated, with hypothetical treatments selectively effective for lesions disrupting defined receptor territories. Each panel shows the same render from a different spatial perspective: **a**, left; **b**, right; **c**, anterior; **d**, posterior, **e**, superior; **f**, inferior. The underlay is a thresholded white matter template surface in MNI.

Supplementary Figure 20: *Mood* subnetwork by receptome slices

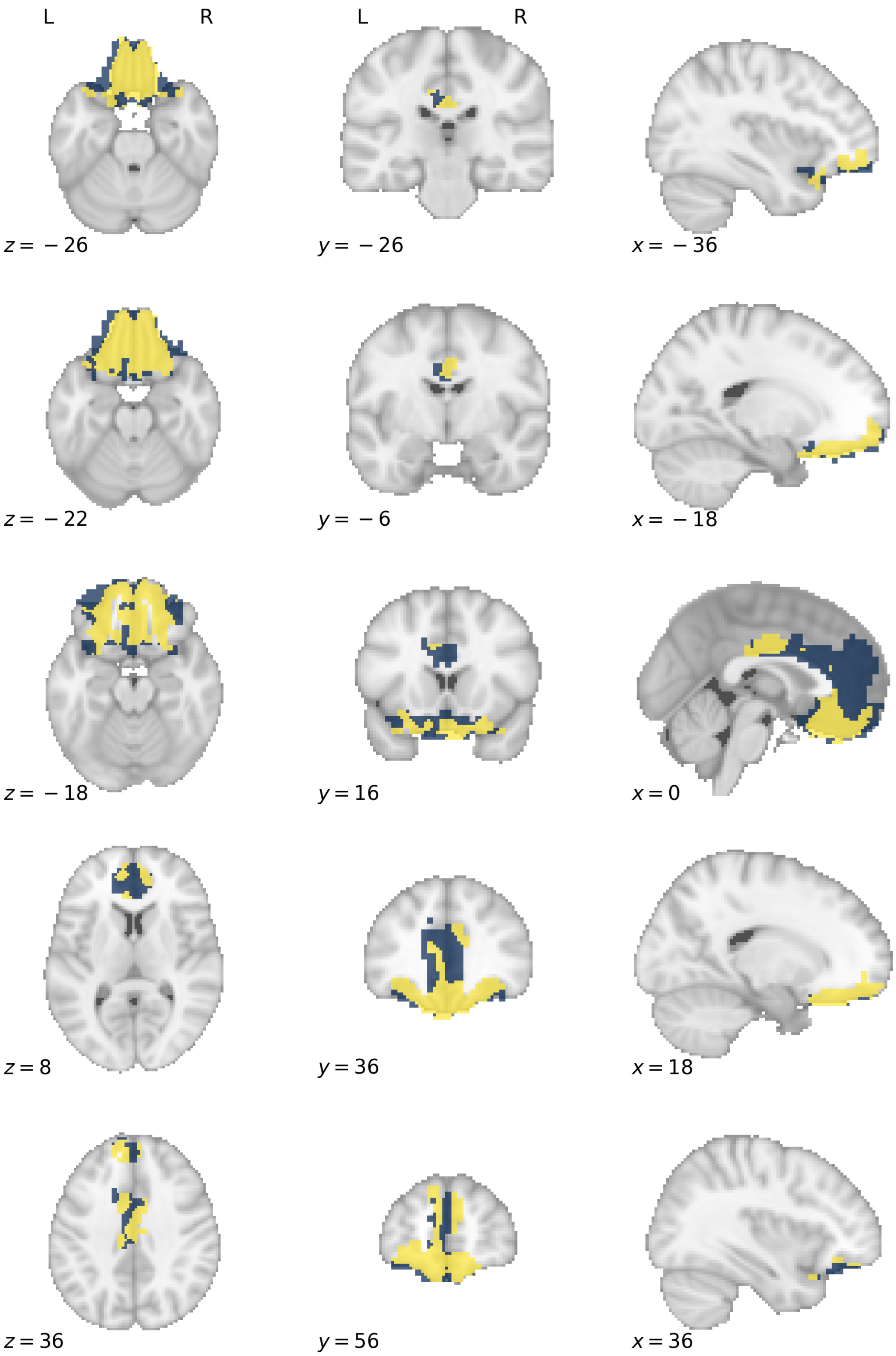

Slice visualization of the functional grey matter network representing *mood*, overlaid onto the standard MNI152 template. The labelled co-ordinates map to MNI space. The colours label functional subnetworks separated by neurotransmitter receptor distribution preponderance, here opioid in yellow and histamine in blue. This forms the basis upon which treatment effect heterogeneity is simulated, with hypothetical treatments selectively effective for lesions disrupting defined receptor territories.

Supplementary Figure 21: *Memory* subnetwork by receptome render

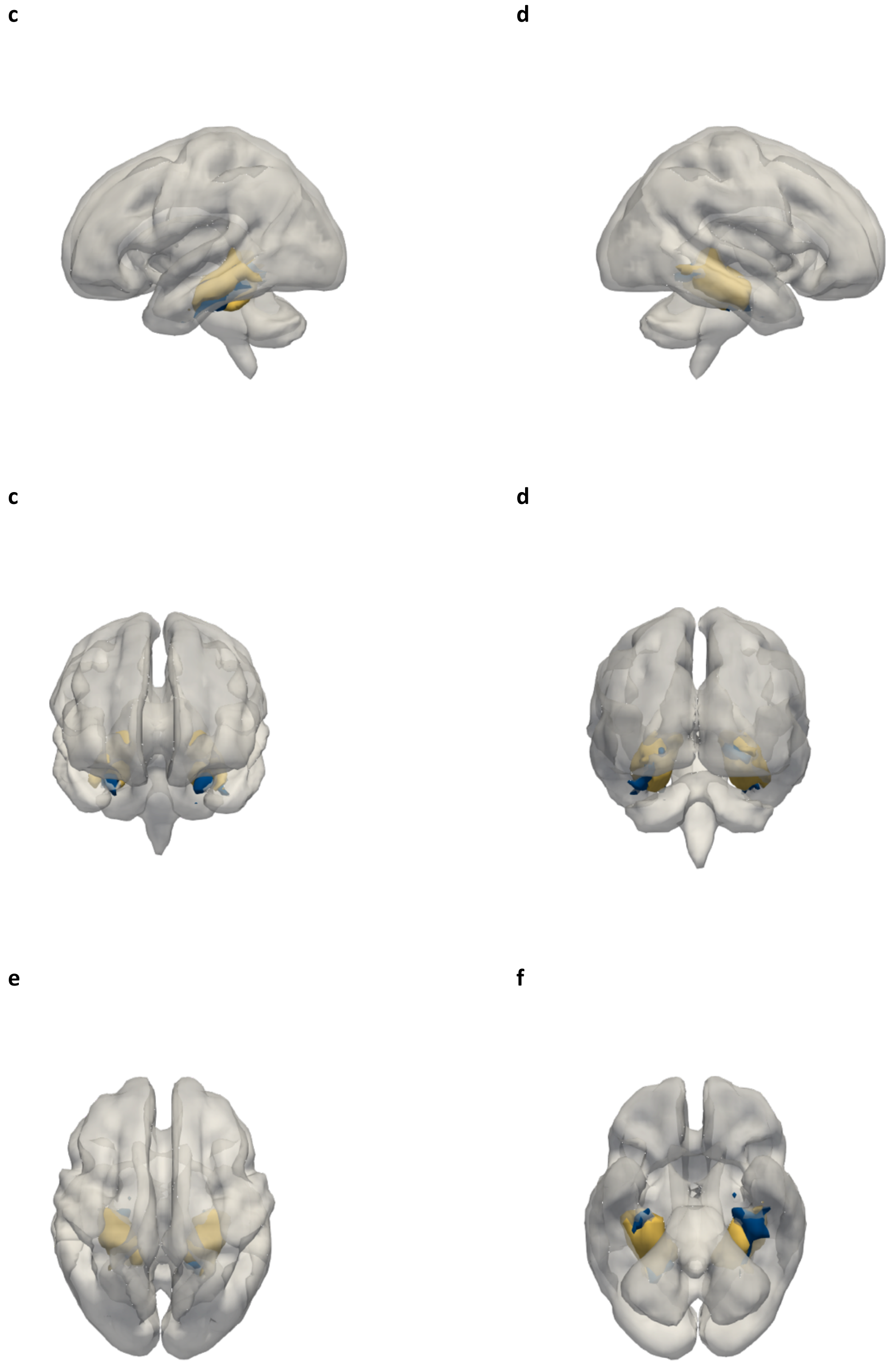

Three-dimensional rendering of the functional grey matter network representing *memory*. The colours label functional subnetworks separated by neurotransmitter receptor distribution preponderance, here dopamine in yellow and 5HT in blue. This forms the basis upon which treatment effect heterogeneity is simulated, with hypothetical treatments selectively effective for lesions disrupting defined receptor territories. Each panel shows the same render from a different spatial perspective: **a**, left; **b**, right; **c**, anterior; **d**, posterior, **e**, superior; **f**, inferior. The underlay is a thresholded white matter template surface in MNI.

Supplementary Figure 22: *Memory* subnetwork by receptome slices

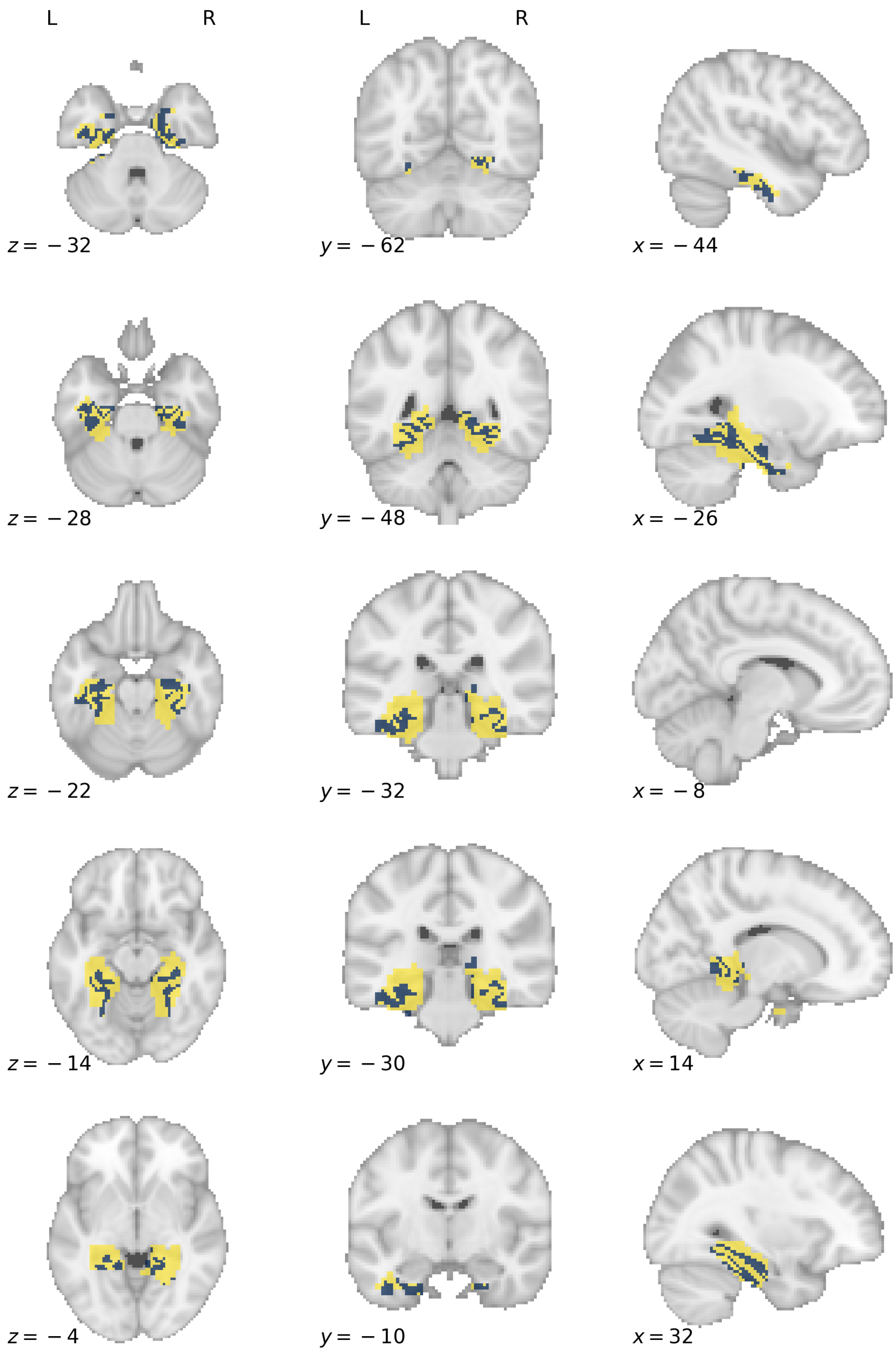

Slice visualization of the functional grey matter network representing *memory*, overlaid onto the standard MNI152 template. The labelled co-ordinates map to MNI space. The colours label functional subnetworks separated by neurotransmitter receptor distribution preponderance, here dopamine in yellow and 5HT in blue. This forms the basis upon which treatment effect heterogeneity is simulated, with hypothetical treatments selectively effective for lesions disrupting defined receptor territories.

Supplementary Figure 23 *Aversion* subnetwork by receptome render

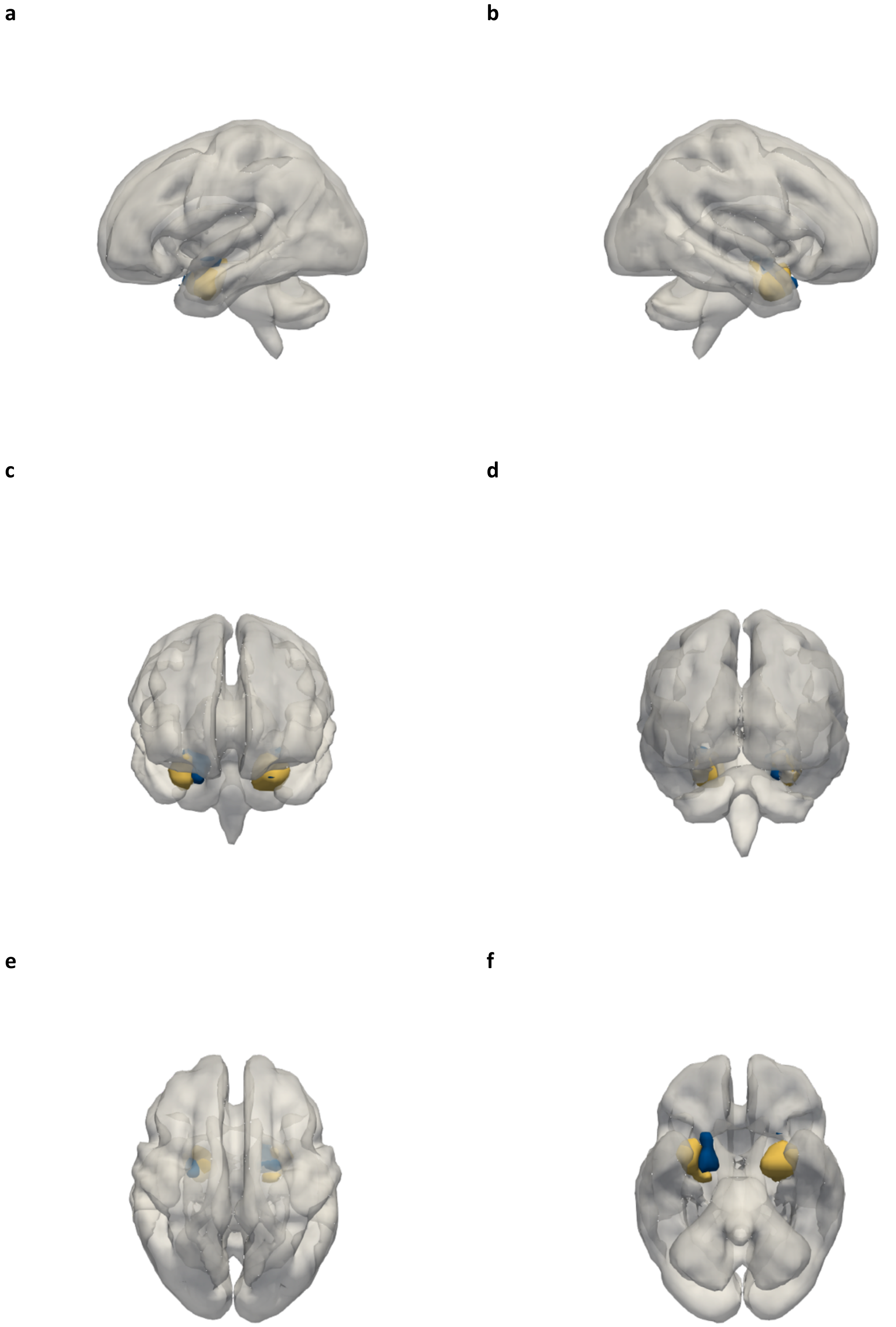

Three-dimensional rendering of the functional grey matter network representing *aversion.* The colours label functional subnetworks separated by neurotransmitter receptor distribution preponderance, here dopamine in yellow and histamine in blue. This forms the basis upon which treatment effect heterogeneity is simulated, with hypothetical treatments selectively effective for lesions disrupting defined receptor territories. Each panel shows the same render from a different spatial perspective: **a**, left; **b**, right; **c**, anterior; **d**, posterior, **e**, superior; **f**, inferior. The underlay is a thresholded white matter template surface in MNI.

Supplementary Figure 24: *Aversion* subnetwork by receptome slices

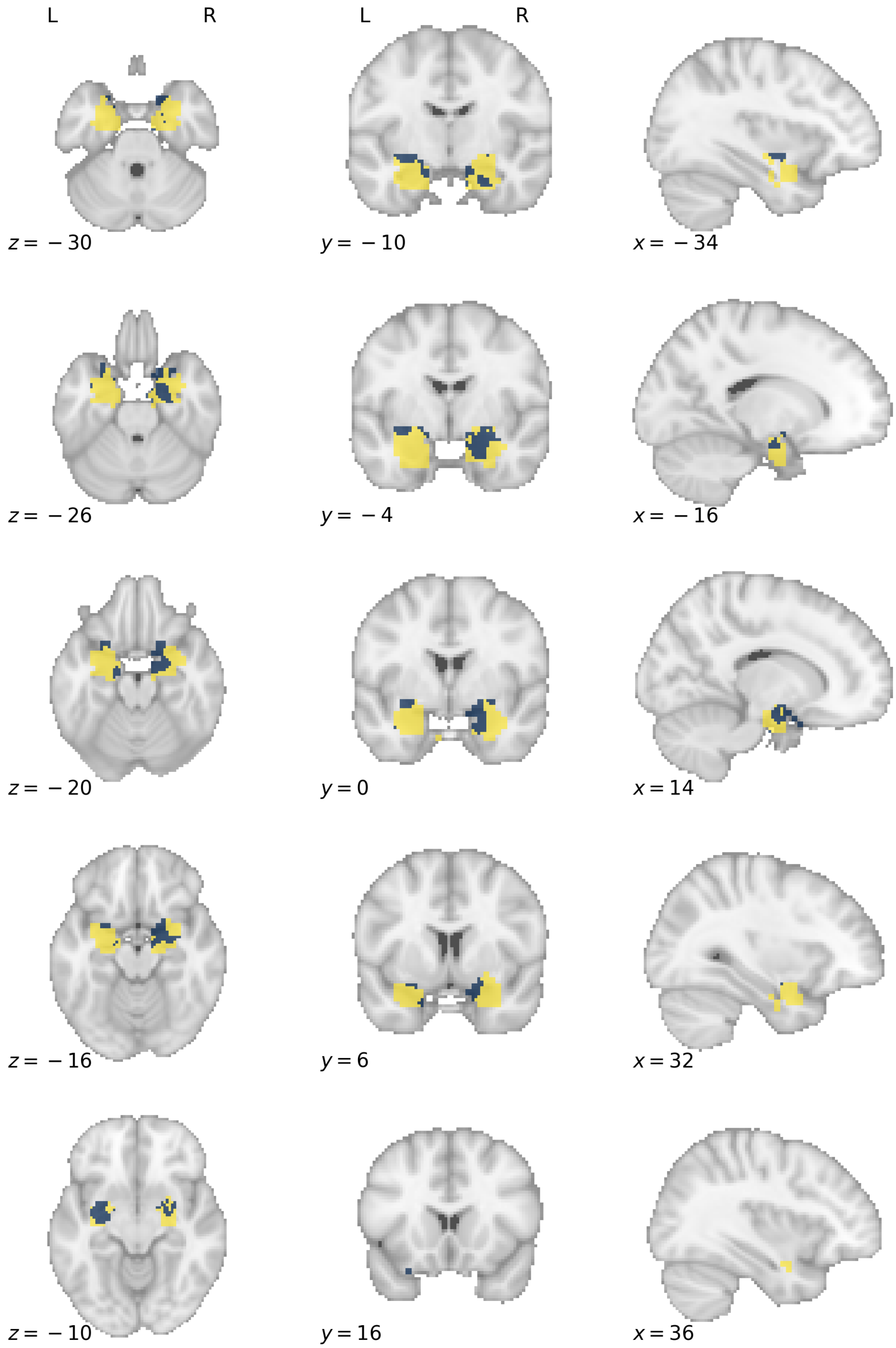

Slice visualization of the functional grey matter network representing *aversion*, overlaid onto the standard MNI152 template. The labelled co-ordinates map to MNI space. The colours label functional subnetworks separated by neurotransmitter receptor distribution preponderance, here dopamine in yellow and histamine in blue. This forms the basis upon which treatment effect heterogeneity is simulated, with hypothetical treatments selectively effective for lesions disrupting defined receptor territories.

Supplementary Figure 25: *Coordination* subnetwork by receptome render

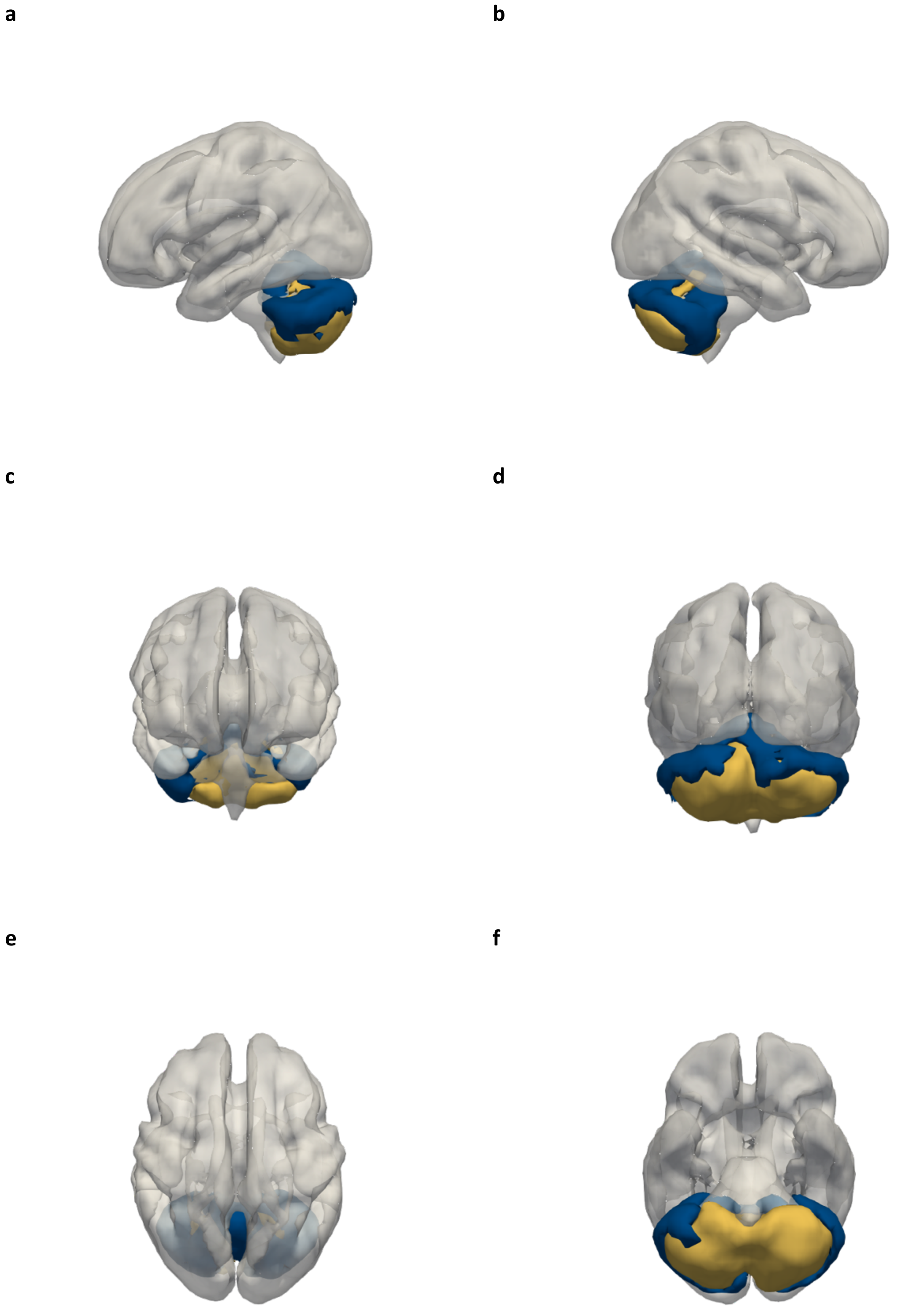

Three-dimensional rendering of the functional grey matter network representing *coordination*. The colours label functional subnetworks separated by neurotransmitter receptor distribution preponderance, here opioid in yellow and acetylcholine in blue. This forms the basis upon which treatment effect heterogeneity is simulated, with hypothetical treatments selectively effective for lesions disrupting defined receptor territories. Each panel shows the same render from a different spatial perspective: **a**, left; **b**, right; **c**, anterior; **d**, posterior, **e**, superior; **f**, inferior. The underlay is a thresholded white matter template surface in MNI.

Supplementary Figure 26: *Coordination* subnetwork by receptome slices

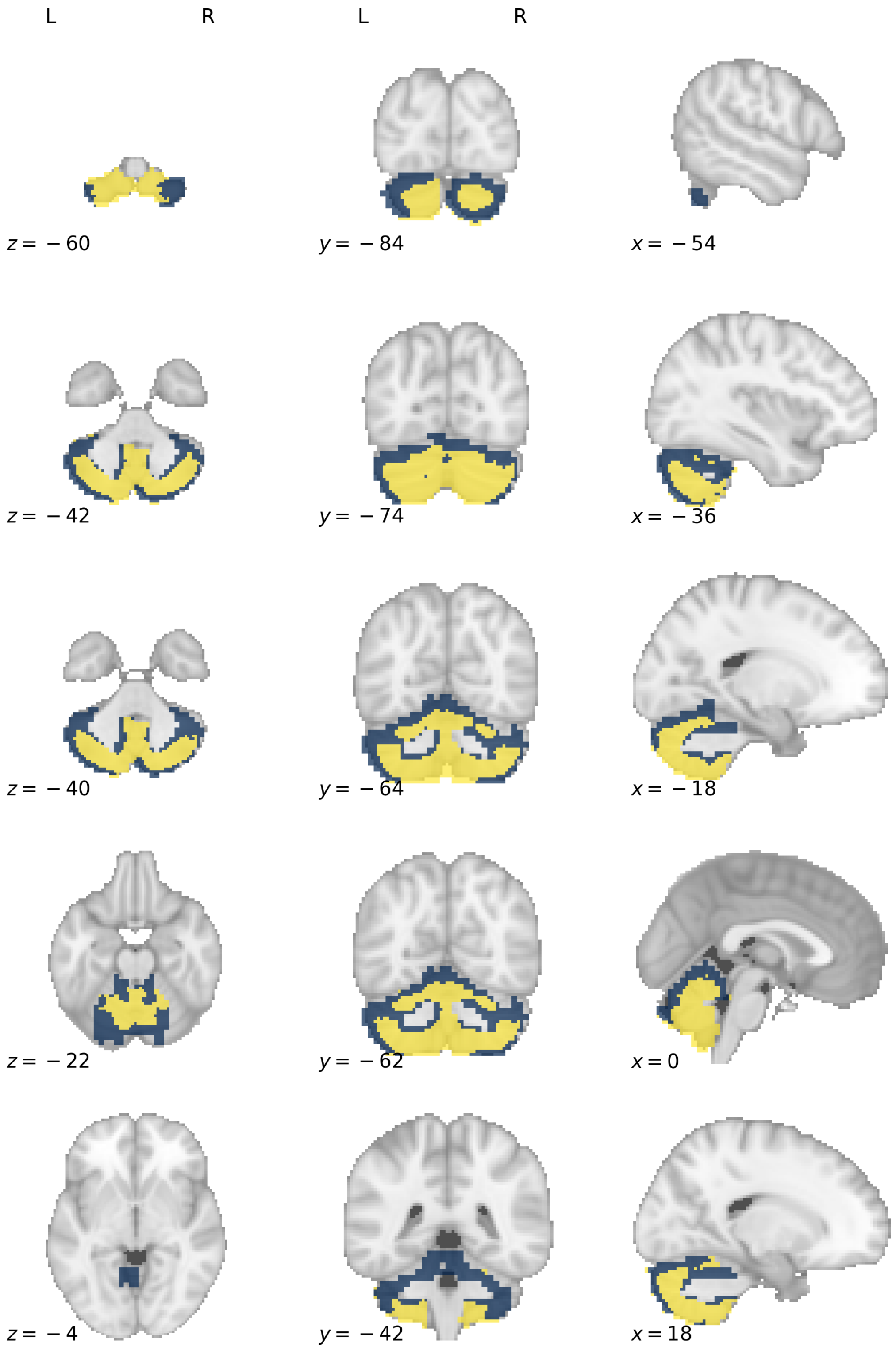

Slice visualization of the functional grey matter network representing *coordination*, overlaid onto the standard MNI152 template. The labelled co-ordinates map to MNI space. The colours label functional subnetworks separated by neurotransmitter receptor distribution preponderance, here opioid in yellow and acetylcholine in blue. This forms the basis upon which treatment effect heterogeneity is simulated, with hypothetical treatments selectively effective for lesions disrupting defined receptor territories.

Supplementary Figure 27: *Interoception* subnetwork by receptome render

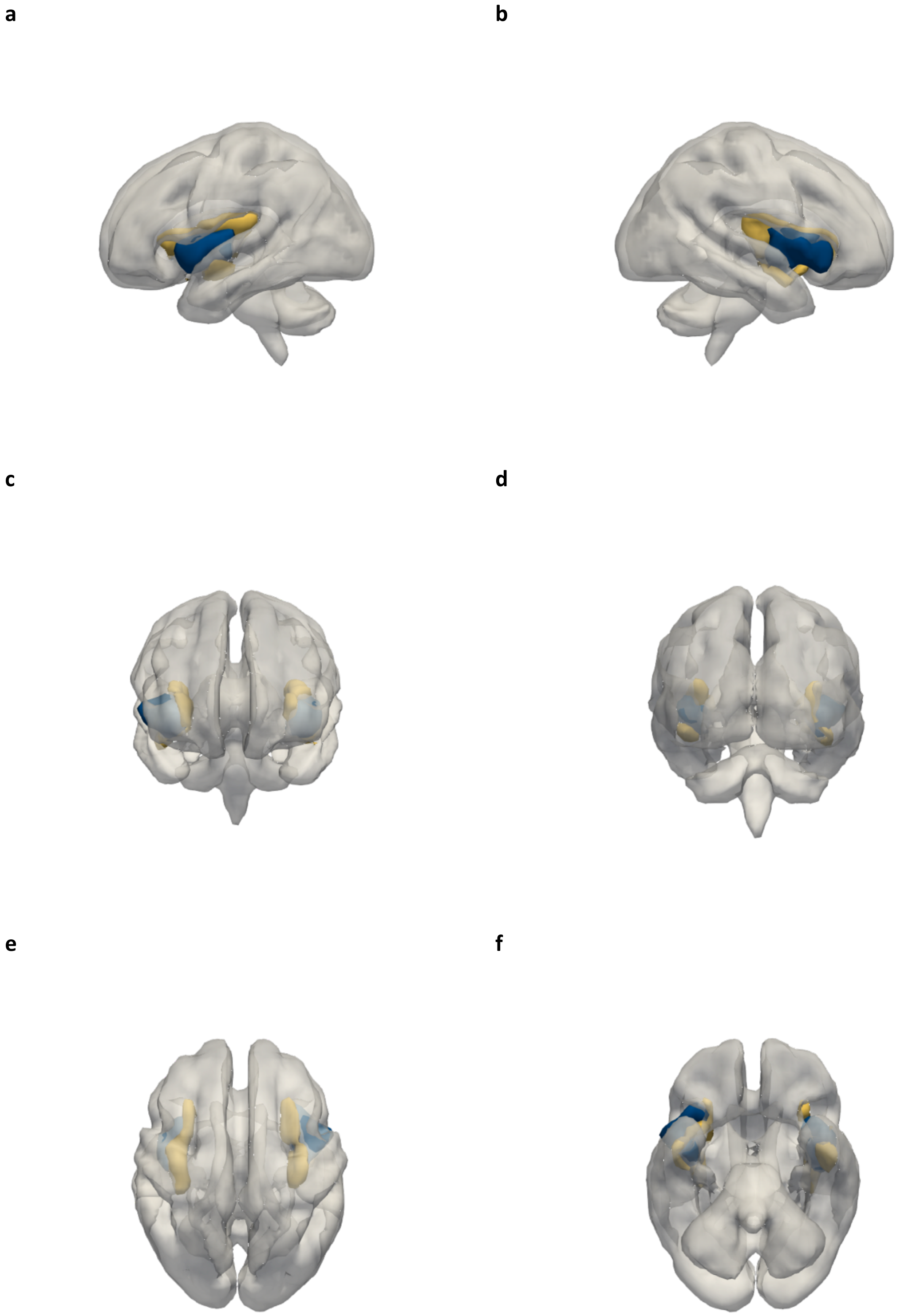

Three-dimensional rendering of the functional grey matter network representing *interoception.* The colours label functional subnetworks separated by neurotransmitter receptor distribution preponderance, here dopamine in yellow and histamine in blue. This forms the basis upon which treatment effect heterogeneity is simulated, with hypothetical treatments selectively effective for lesions disrupting defined receptor territories. Each panel shows the same render from a different spatial perspective: **a**, left; **b**, right; **c**, anterior; **d**, posterior, **e**, superior; **f**, inferior. The underlay is a thresholded white matter template surface in MNI.

Supplementary Figure 28: *Interoception* subnetwork by receptome slices

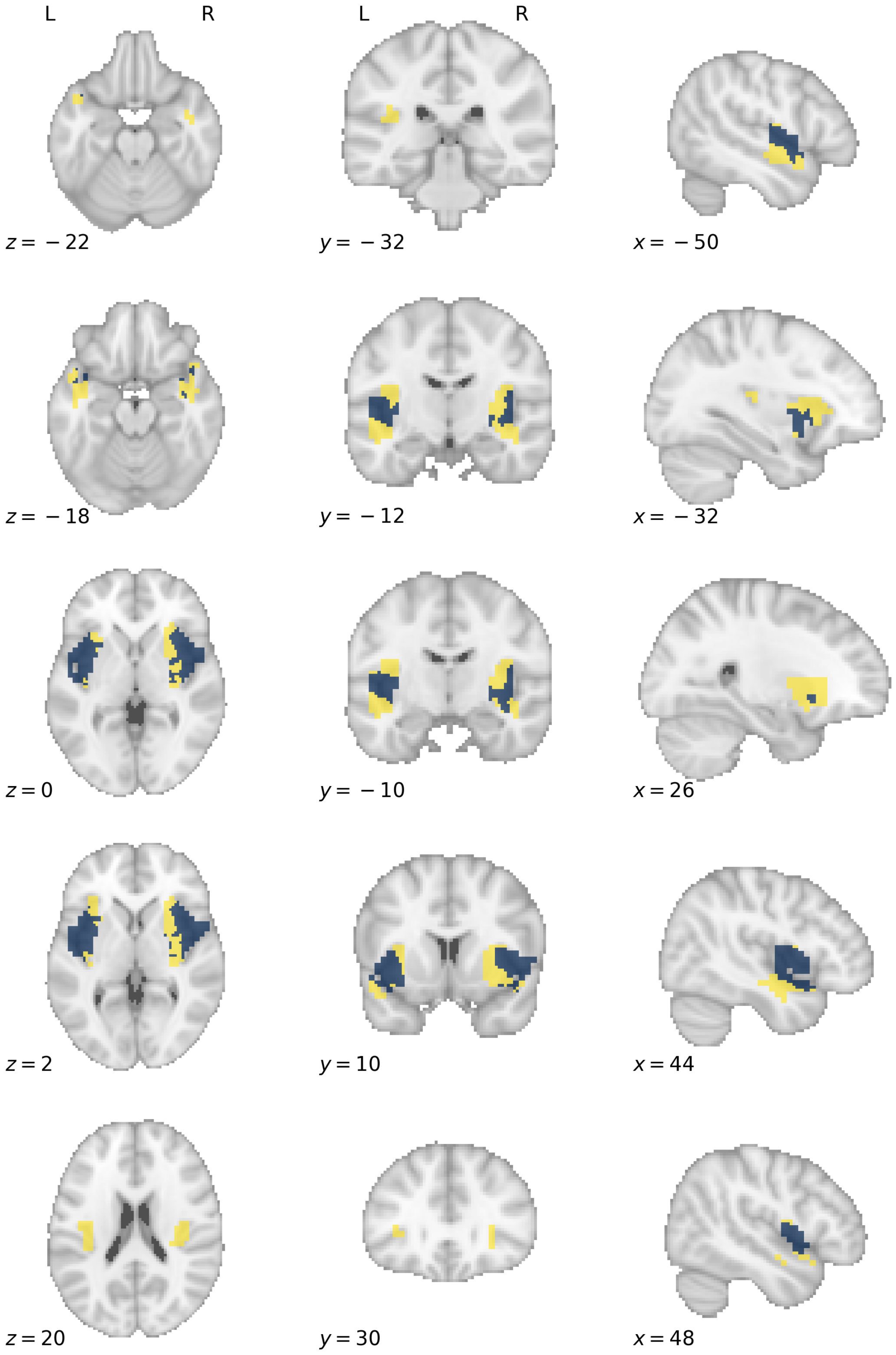

Slice visualization of the functional grey matter network representing *interoception*, overlaid onto the standard MNI152 template. The labelled co-ordinates map to MNI space. The colours label functional subnetworks separated by neurotransmitter receptor distribution preponderance, here dopamine in yellow and histamine in blue. This forms the basis upon which treatment effect heterogeneity is simulated, with hypothetical treatments selectively effective for lesions disrupting defined receptor territories.

Supplementary Figure 29: *Sleep* subnetwork by receptome render

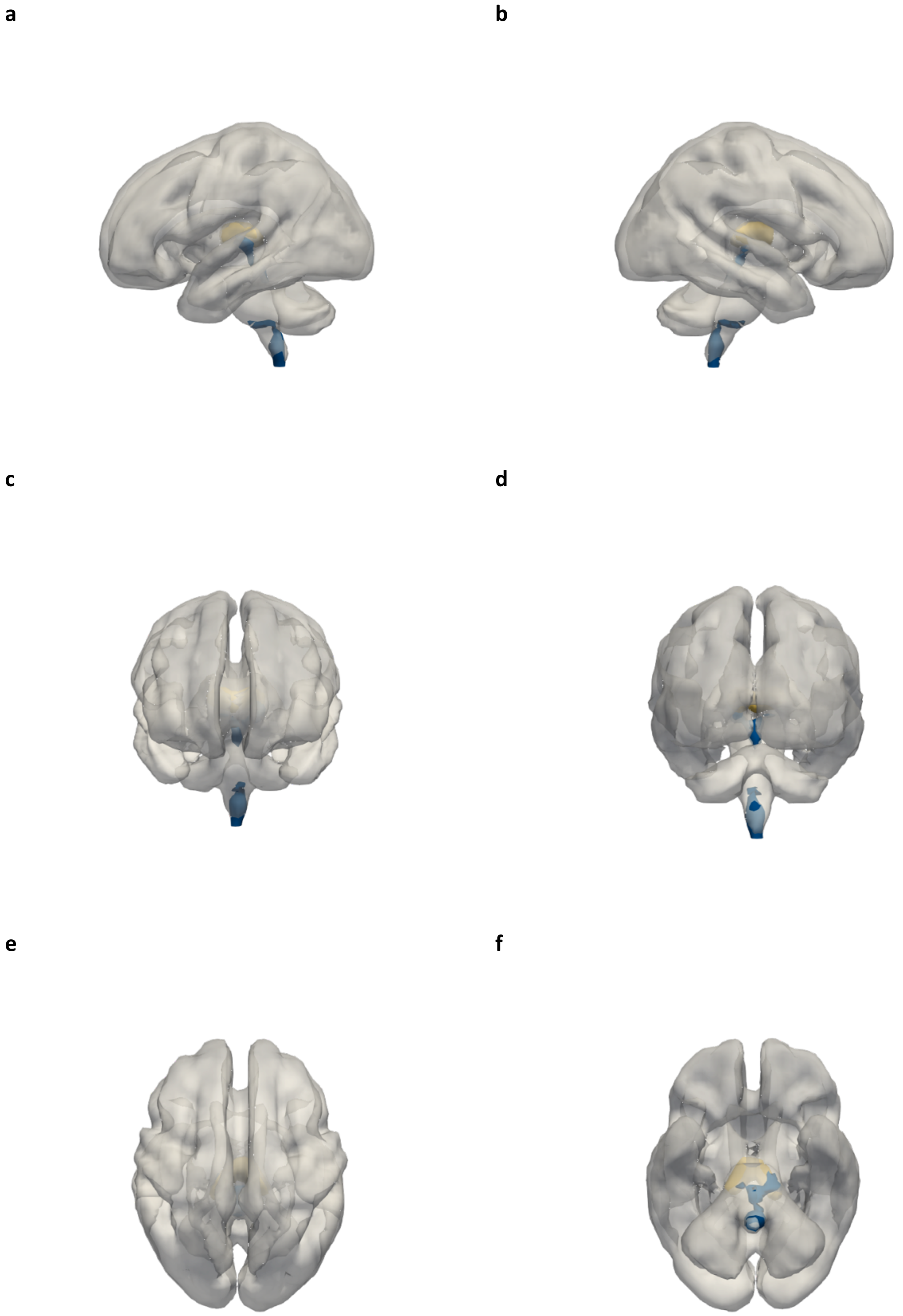

Three-dimensional rendering of the functional grey matter network representing *sleep*. The colours label functional subnetworks separated by neurotransmitter receptor distribution preponderance, here opioid in yellow and noradrenaline in blue. This forms the basis upon which treatment effect heterogeneity is simulated, with hypothetical treatments selectively effective for lesions disrupting defined receptor territories. Each panel shows the same render from a different spatial perspective: **a**, left; **b**, right; **c**, anterior; **d**, posterior, **e**, superior; **f**, inferior. The underlay is a thresholded white matter template surface in MNI.

Supplementary Figure 30: *Sleep* subnetwork by receptome slices

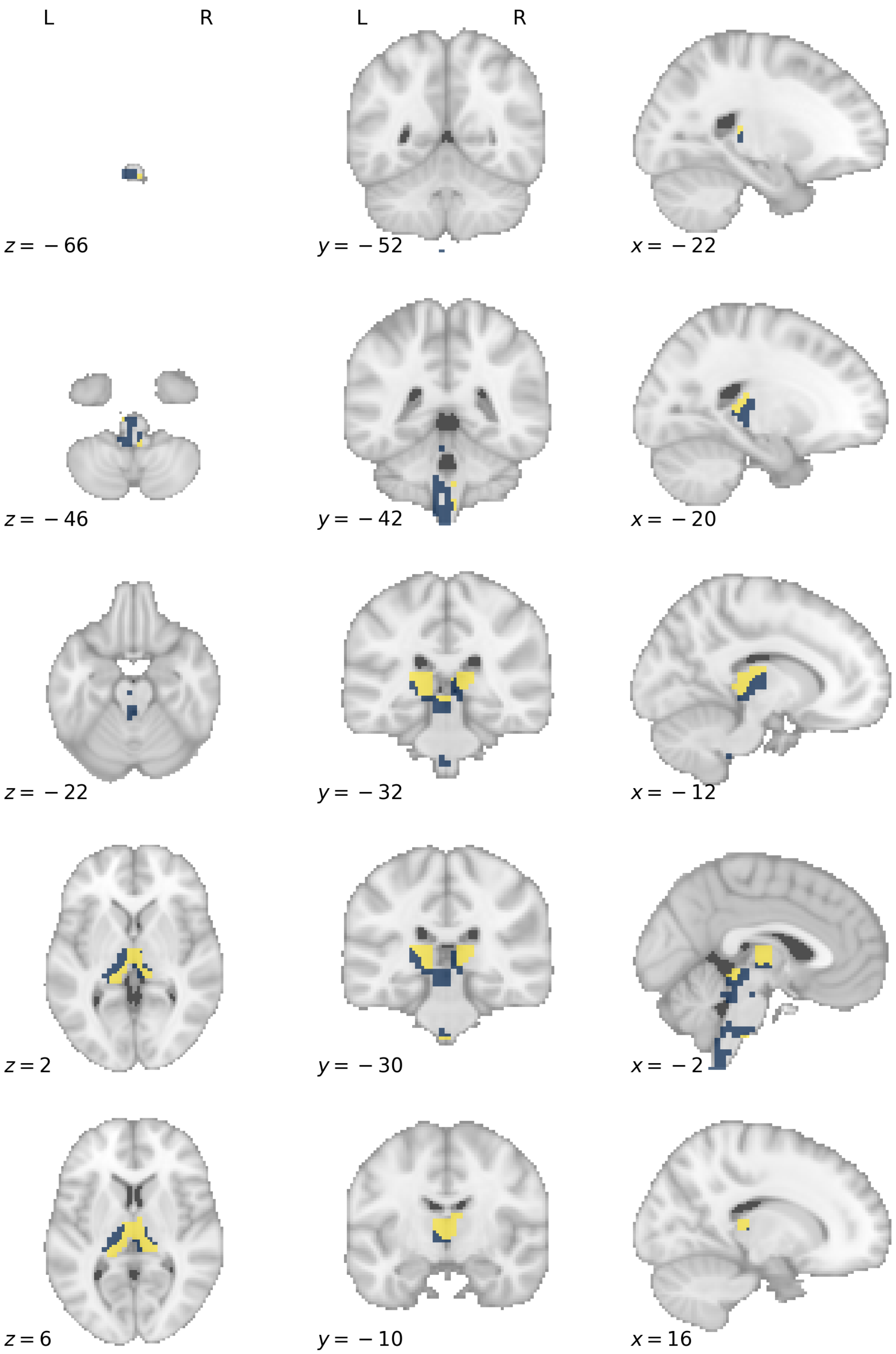

Slice visualization of the functional grey matter network representing *sleep*, overlaid onto the standard MNI152 template. The labelled co-ordinates map to MNI space. The colours label functional subnetworks separated by neurotransmitter receptor distribution preponderance, here opioid in yellow and noradrenaline in blue. This forms the basis upon which treatment effect heterogeneity is simulated, with hypothetical treatments selectively effective for lesions disrupting defined receptor territories.

Supplementary Figure 31: *Reward* subnetwork by receptome render

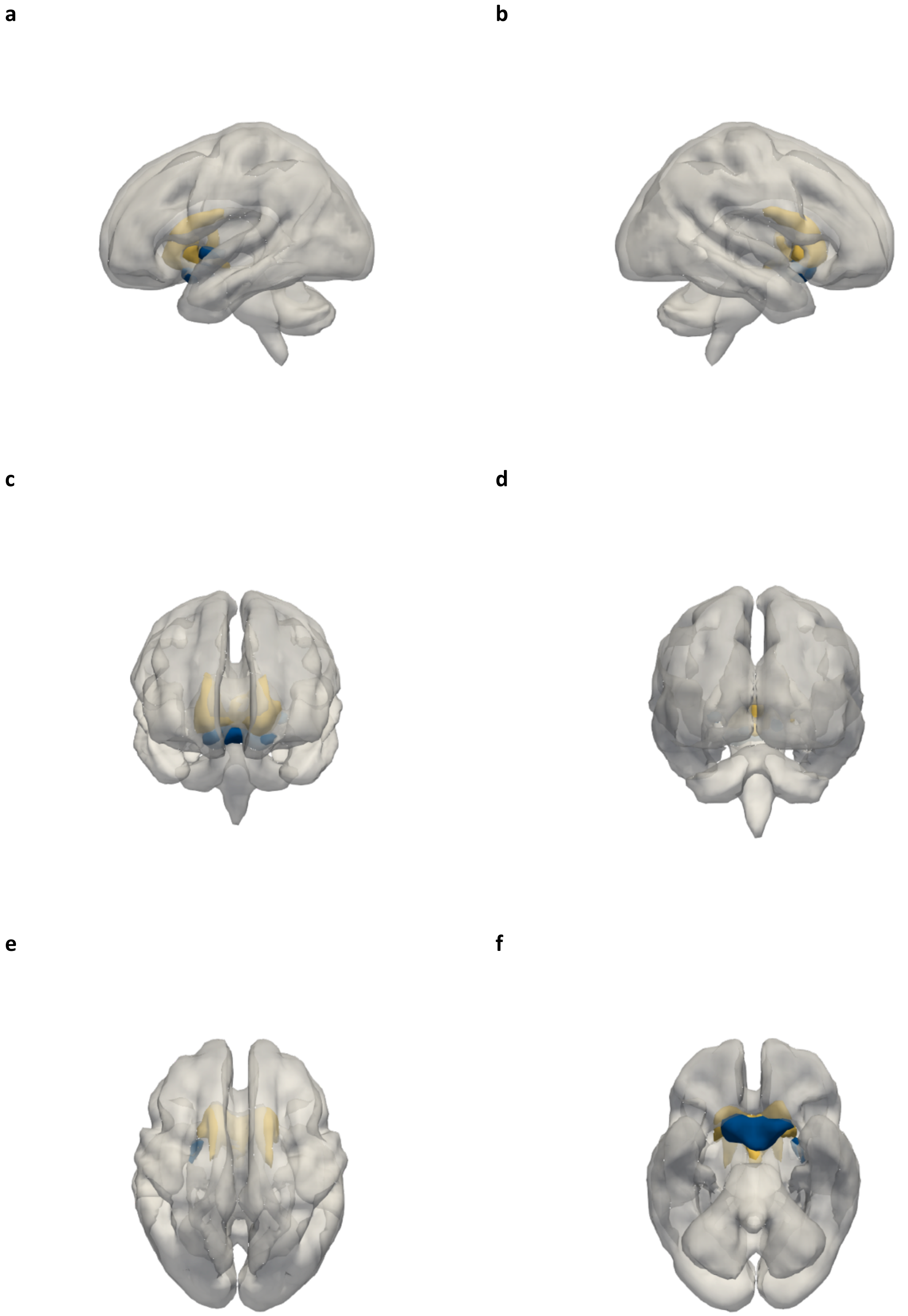

Three-dimensional rendering of the functional grey matter network representing *reward*. The colours label functional subnetworks separated by neurotransmitter receptor distribution preponderance, here dopamine in yellow and histamine in blue. This forms the basis upon which treatment effect heterogeneity is simulated, with hypothetical treatments selectively effective for lesions disrupting defined receptor territories. Each panel shows the same render from a different spatial perspective: **a**, left; **b**, right; **c**, anterior; **d**, posterior, **e**, superior; **f**, inferior. The underlay is a thresholded white matter template surface in MNI.

Supplementary Figure 32: *Reward* subnetwork by receptome slices

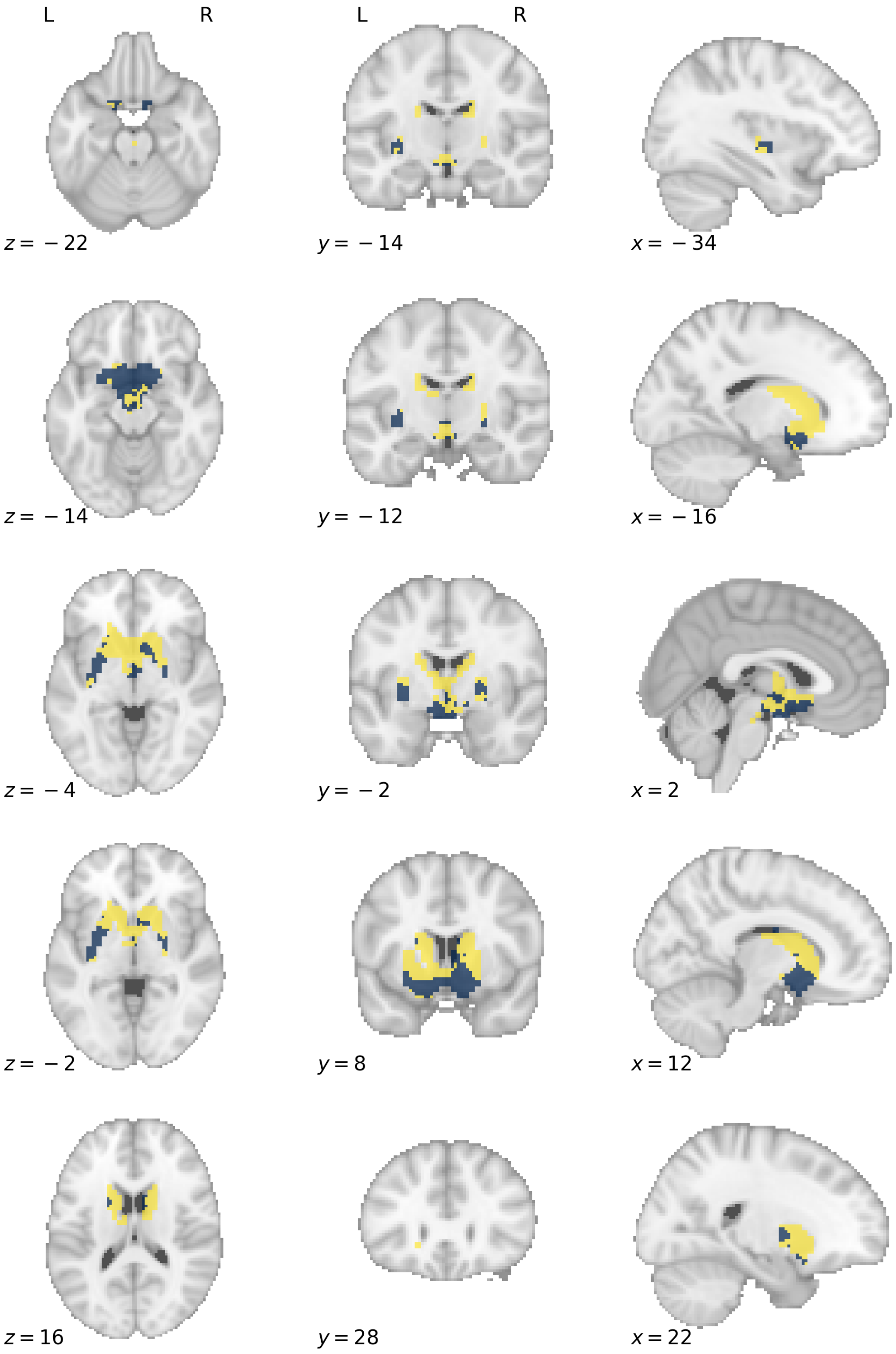

Slice visualization of the functional grey matter network representing *reward*, overlaid onto the standard MNI152 template. The labelled co-ordinates map to MNI space. The colours label functional subnetworks separated by neurotransmitter receptor distribution preponderance, here dopamine in yellow and histamine in blue. This forms the basis upon which treatment effect heterogeneity is simulated, with hypothetical treatments selectively effective for lesions disrupting defined receptor territories.

Supplementary Figure 33: *Visual Recognition* subnetwork by receptome render

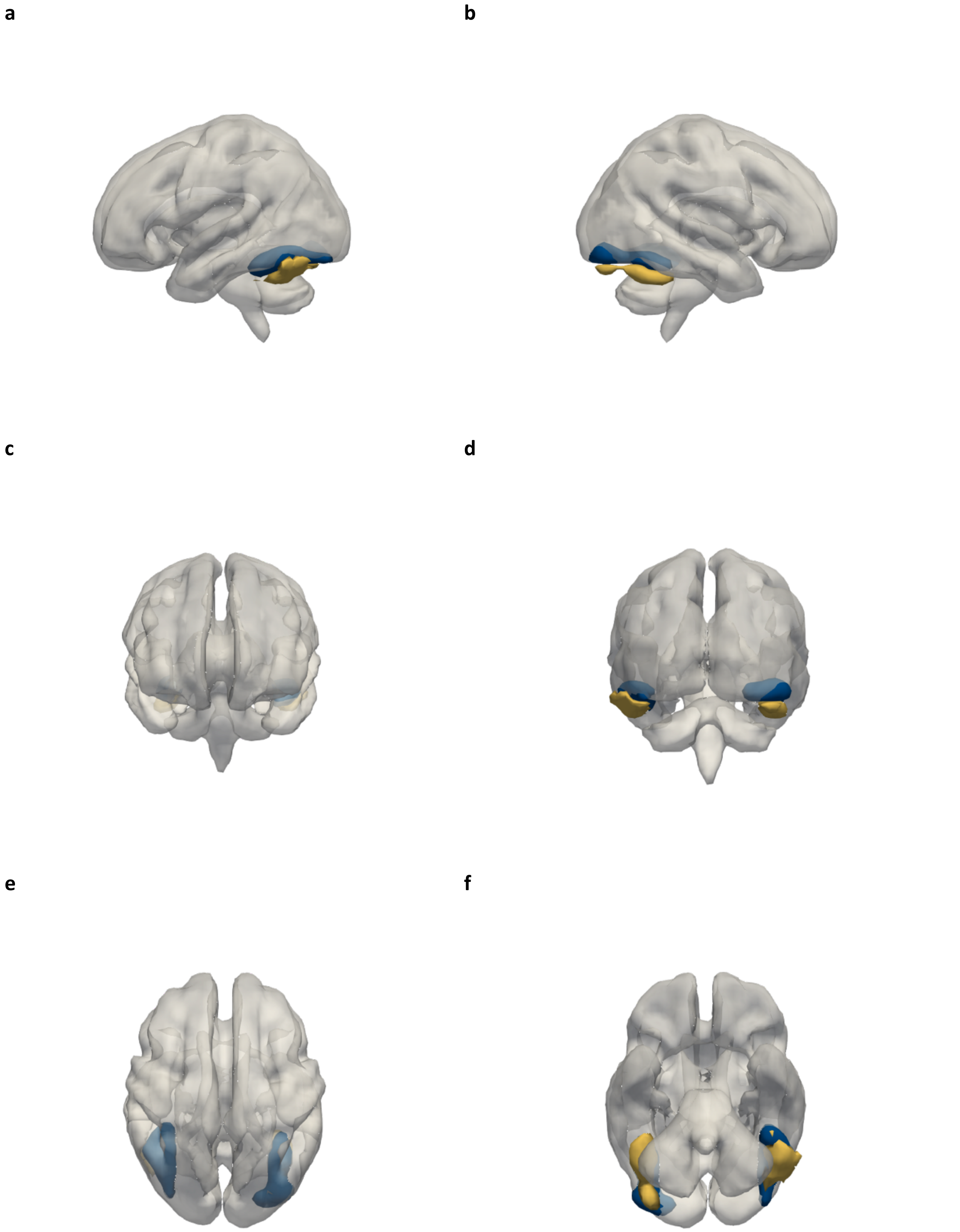

Three-dimensional rendering of the functional grey matter network representing *visual recognition*. The colours label functional subnetworks separated by neurotransmitter receptor distribution preponderance, here cannabinoid in yellow and GABA in blue. This forms the basis upon which treatment effect heterogeneity is simulated, with hypothetical treatments selectively effective for lesions disrupting defined receptor territories. Each panel shows the same render from a different spatial perspective: **a**, left; **b**, right; **c**, anterior; **d**, posterior, **e**, superior; **f**, inferior. The underlay is a thresholded white matter template surface in MNI.

Supplementary Figure 34: *Visual Recognition* subnetwork by receptome slices

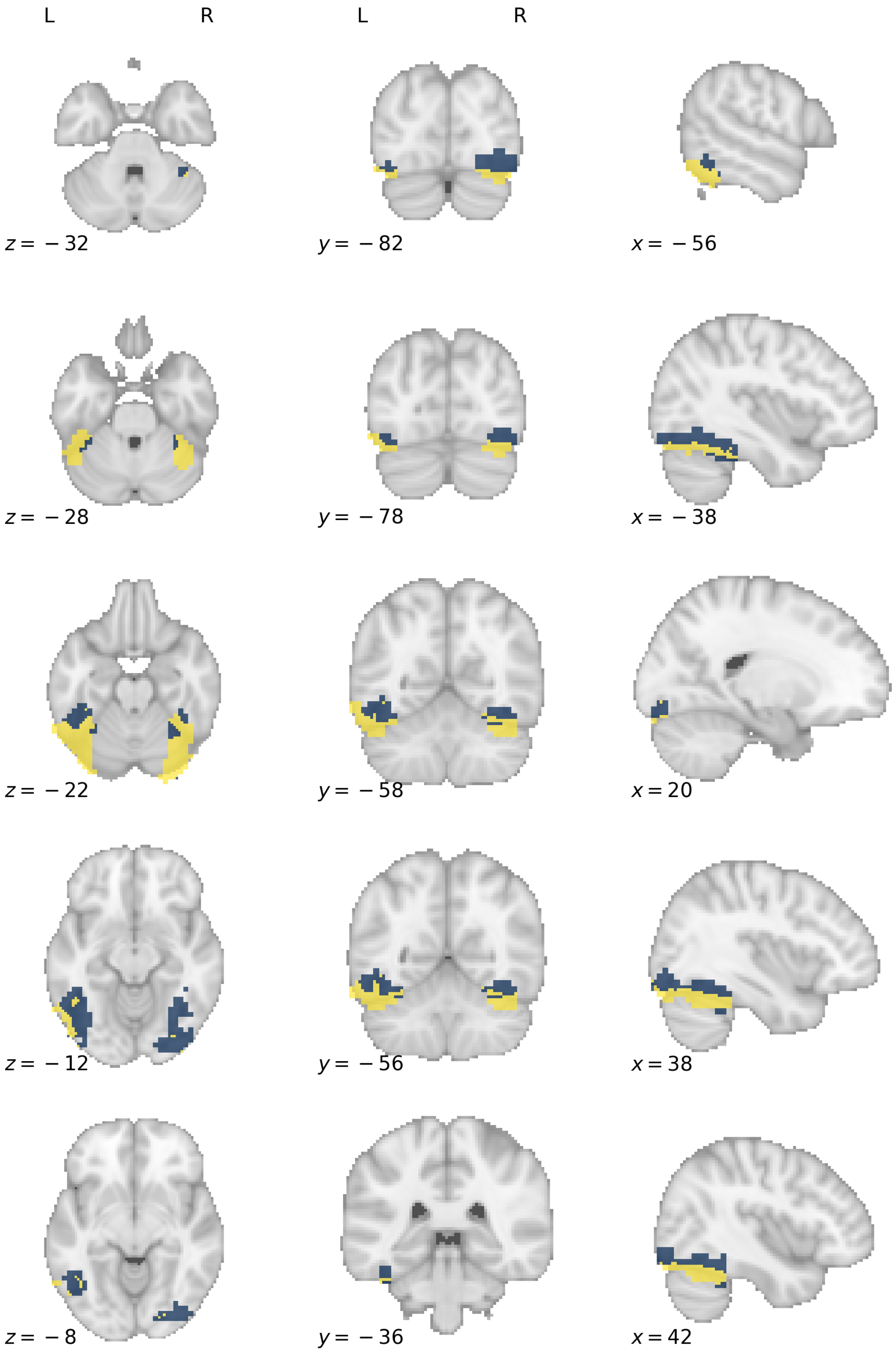

Slice visualization of the functional grey matter network representing *visual recognition*, overlaid onto the standard MNI152 template. The labelled co-ordinates map to MNI space. The colours label functional subnetworks separated by neurotransmitter receptor distribution preponderance, here cannabinoid in yellow and GABA in blue. This forms the basis upon which treatment effect heterogeneity is simulated, with hypothetical treatments selectively effective for lesions disrupting defined receptor territories.

Supplementary Figure 35: *Visual Perception* subnetwork by receptome render

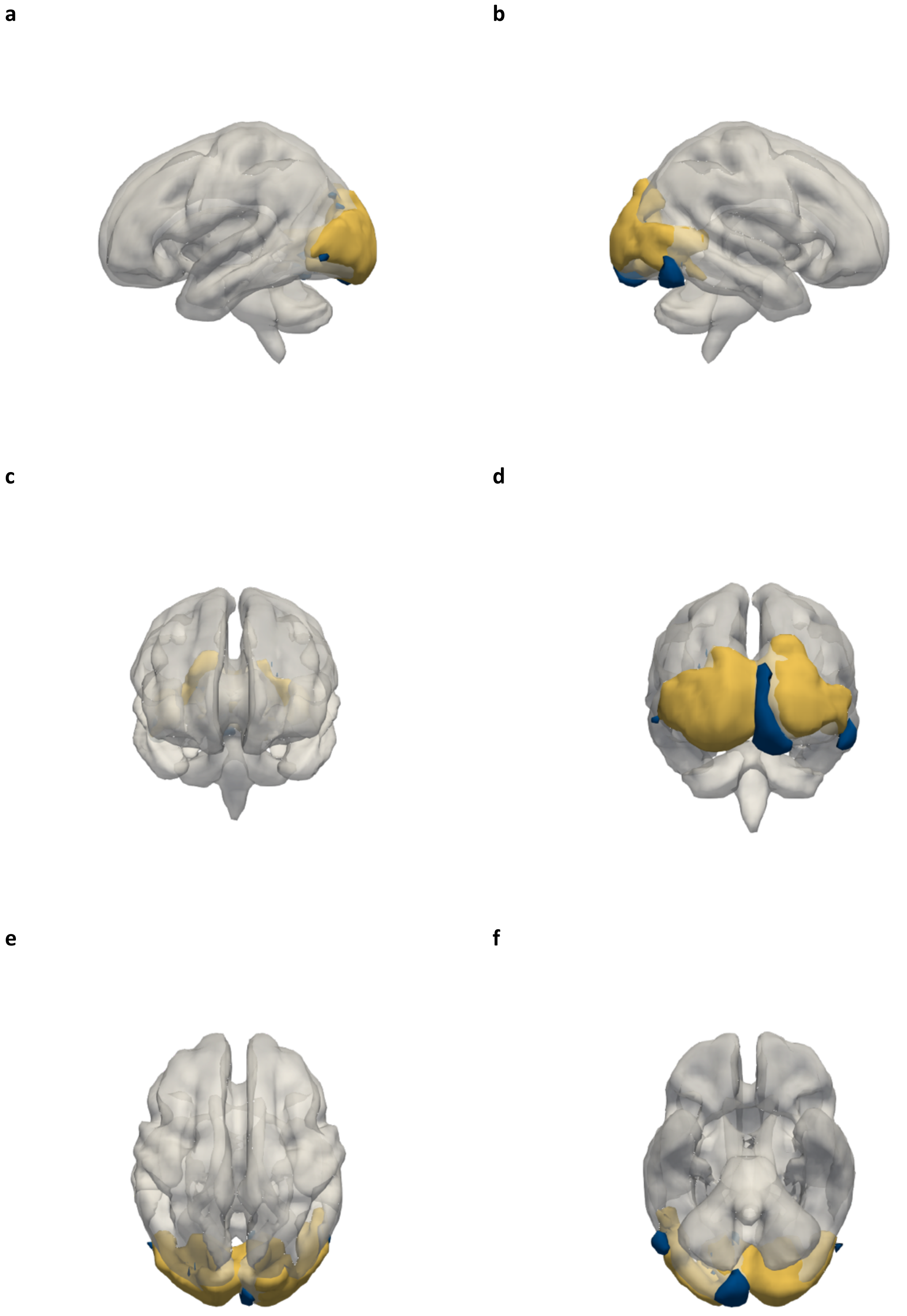

Three-dimensional rendering of the functional grey matter network representing *visual perception*. The colours label functional subnetworks separated by neurotransmitter receptor distribution preponderance, here GABA in yellow and 5HT in blue. This forms the basis upon which treatment effect heterogeneity is simulated, with hypothetical treatments selectively effective for lesions disrupting defined receptor territories. Each panel shows the same render from a different spatial perspective: **a**, left; **b**, right; **c**, anterior; **d**, posterior, **e**, superior; **f**, inferior. The underlay is a thresholded white matter template surface in MNI.

Supplementary Figure 36: *Visual Perception* subnetwork by receptome slices

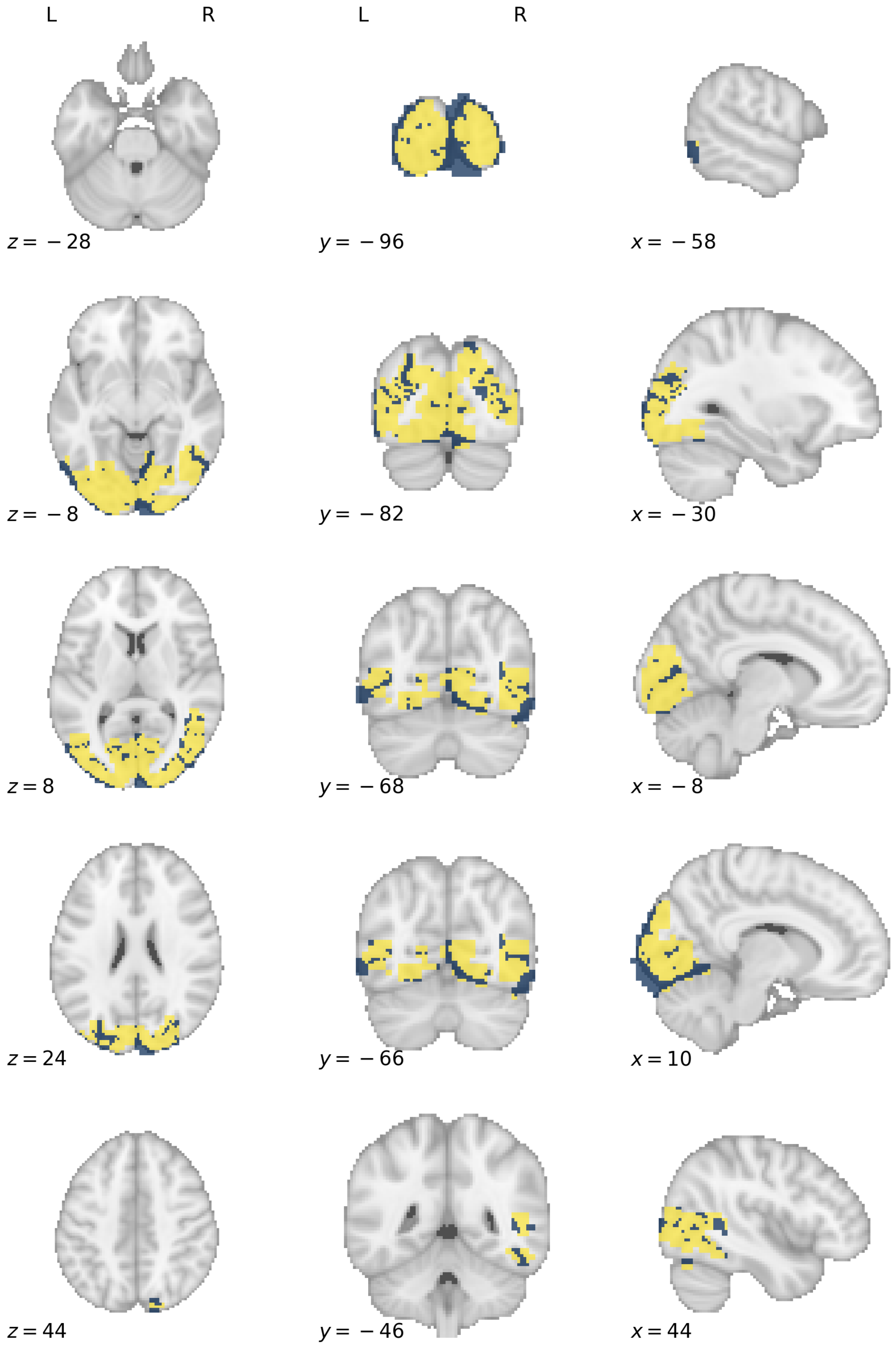

Slice visualization of the functional grey matter network representing *visual perception*, overlaid onto the standard MNI152 template. The labelled co-ordinates map to MNI space. The colours label functional subnetworks separated by neurotransmitter receptor distribution preponderance, here GABA in yellow and 5HT in blue. This forms the basis upon which treatment effect heterogeneity is simulated, with hypothetical treatments selectively effective for lesions disrupting defined receptor territories.

Supplementary Figure 37: *Spatial Reasoning* subnetwork by receptome render

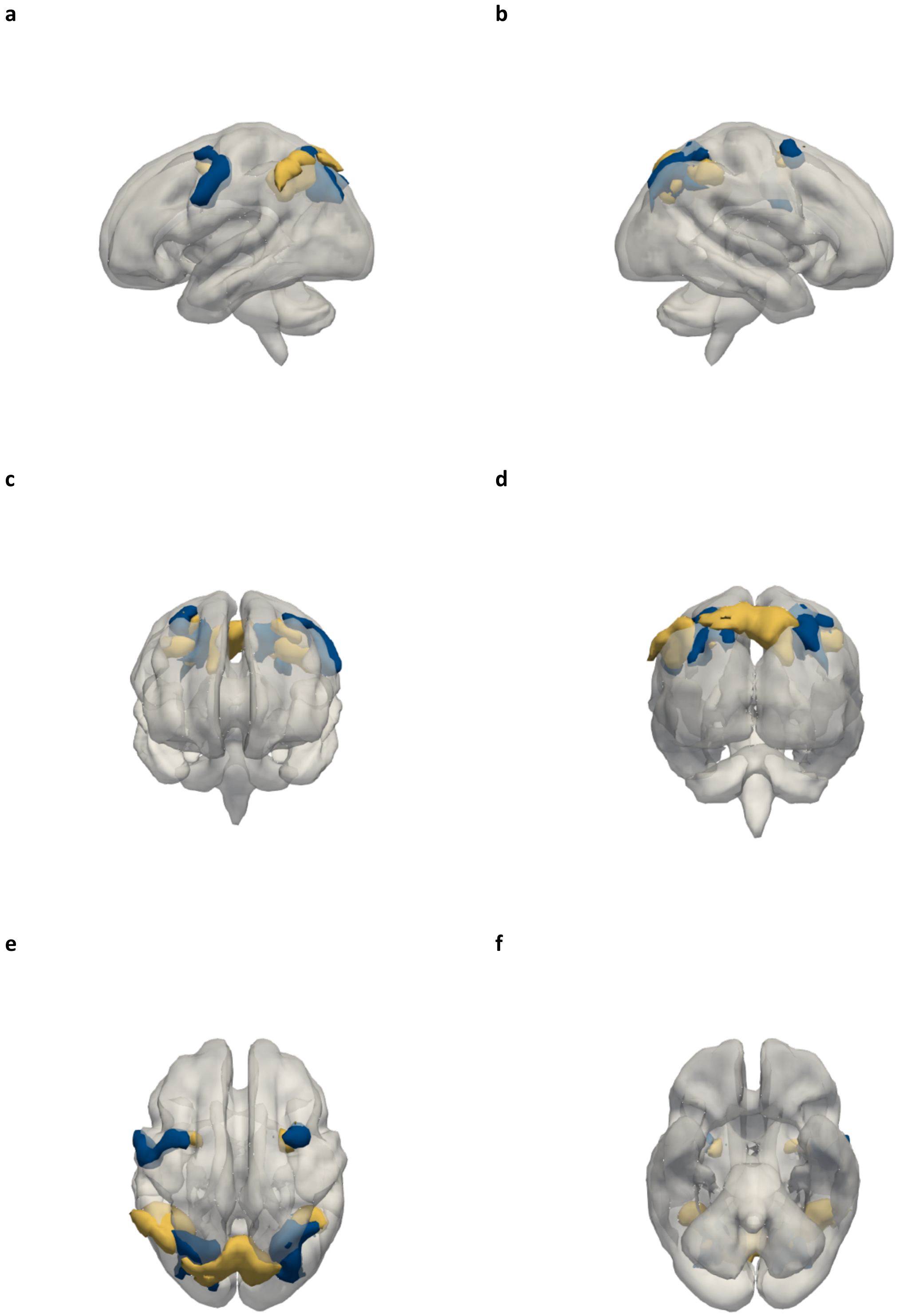

Three-dimensional rendering of the functional grey matter network representing *spatial reasoning.* The colours label functional subnetworks separated by neurotransmitter receptor distribution preponderance, here glutamate in yellow and noradrenaline in blue. This forms the basis upon which treatment effect heterogeneity is simulated, with hypothetical treatments selectively effective for lesions disrupting defined receptor territories. Each panel shows the same render from a different spatial perspective: **a**, left; **b**, right; **c**, anterior; **d**, posterior, **e**, superior; **f**, inferior. The underlay is a thresholded white matter template surface in MNI.

Supplementary Figure 38: *Spatial Reasoning* subnetwork by receptome slices

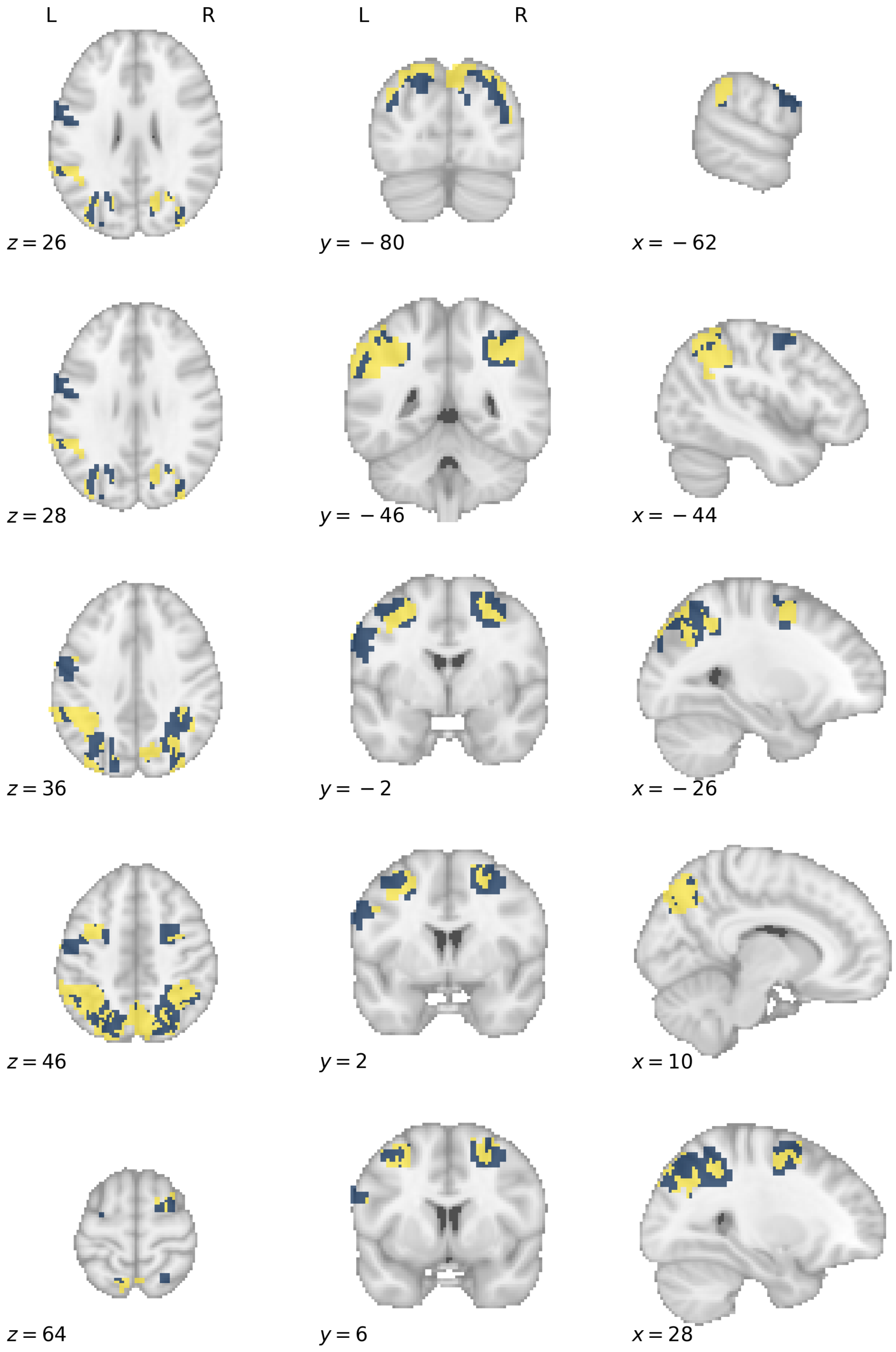

Slice visualization of the functional grey matter network representing *spatial reasoning*, overlaid onto the standard MNI152 template. The labelled co-ordinates map to MNI space. The colours label functional subnetworks separated by neurotransmitter receptor distribution preponderance, here glutamate in yellow and noradrenaline in blue. This forms the basis upon which treatment effect heterogeneity is simulated, with hypothetical treatments selectively effective for lesions disrupting defined receptor territories.

Supplementary Figure 39: *Motor* subnetwork by receptome render

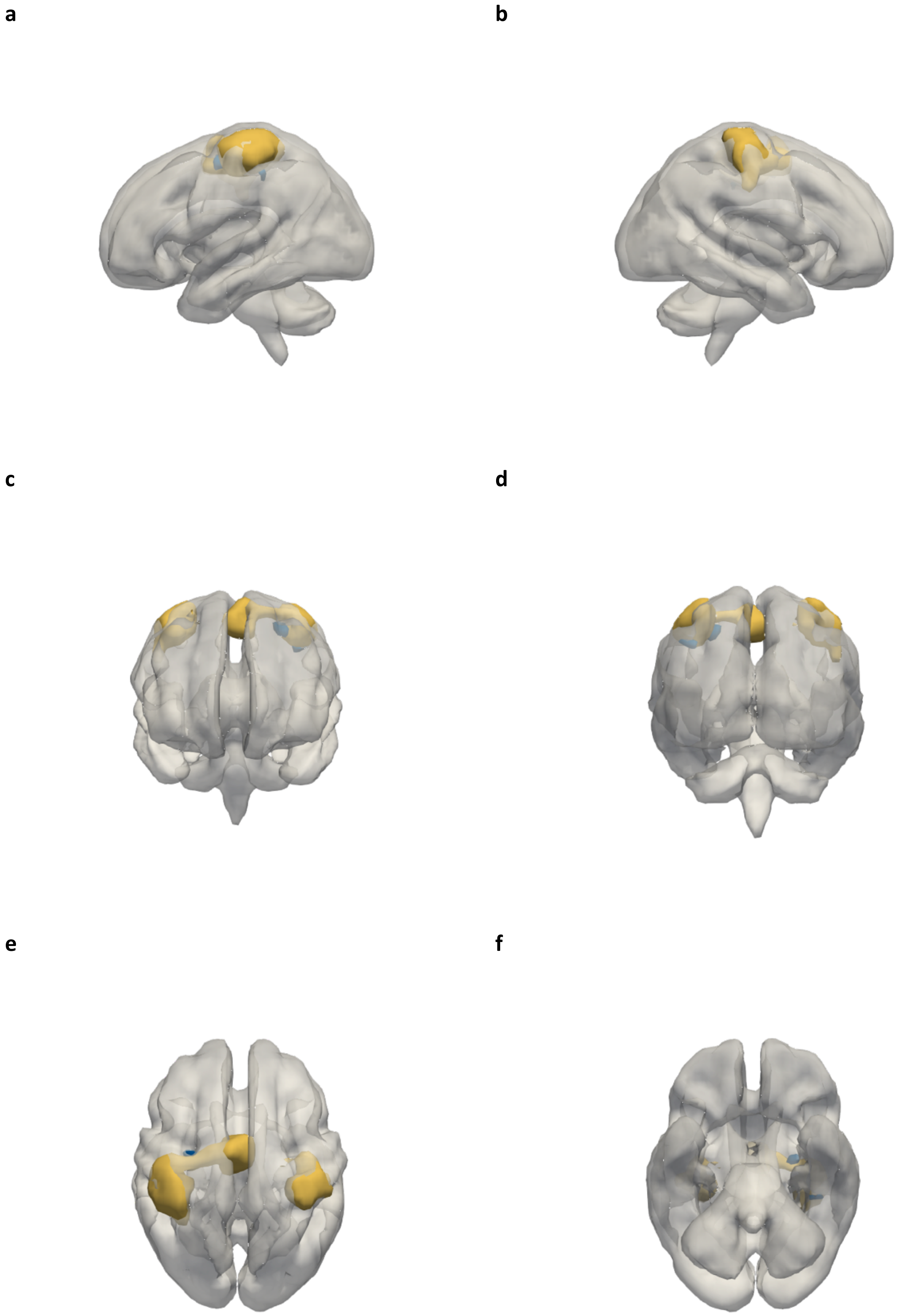

Three-dimensional rendering of the functional grey matter network representing *motor behaviour*. The colours label functional subnetworks separated by neurotransmitter receptor distribution preponderance, here noradrenaline in yellow and glutamate in blue. This forms the basis upon which treatment effect heterogeneity is simulated, with hypothetical treatments selectively effective for lesions disrupting defined receptor territories. Each panel shows the same render from a different spatial perspective: **a**, left; **b**, right; **c**, anterior; **d**, posterior, **e**, superior; **f**, inferior. The underlay is a thresholded white matter template surface in MNI.

Supplementary Figure 40: *Motor* subnetwork by receptome slices

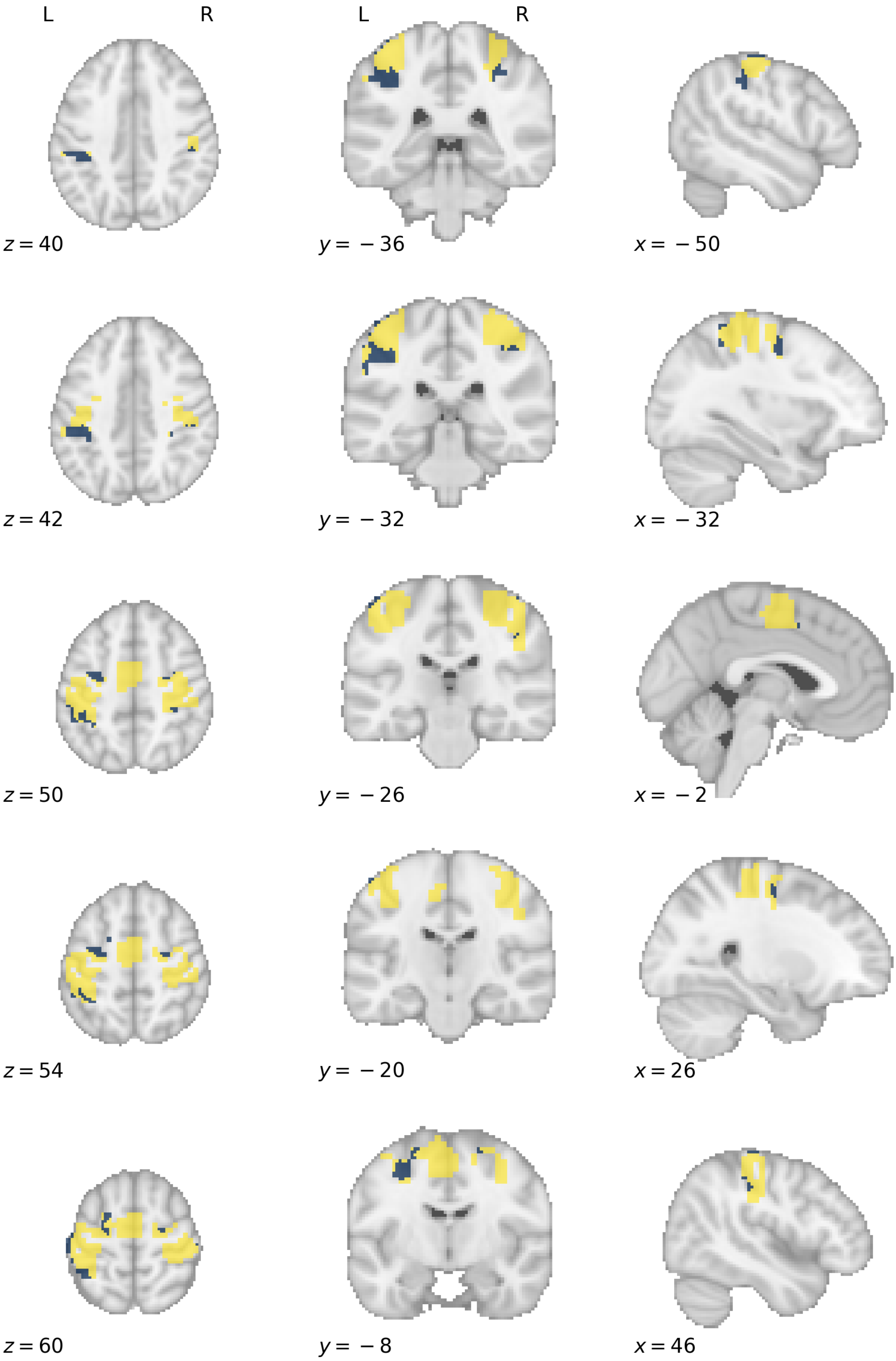

Slice visualization of the functional grey matter network representing *motor behaviour*, overlaid onto the standard MNI152 template. The labelled co-ordinates map to MNI space. The colours label functional subnetworks separated by neurotransmitter receptor distribution preponderance, here noradrenaline in yellow and glutamate in blue. This forms the basis upon which treatment effect heterogeneity is simulated, with hypothetical treatments selectively effective for lesions disrupting defined receptor territories.

Supplementary Figure 41: *Somatosensory* subnetwork by receptome render

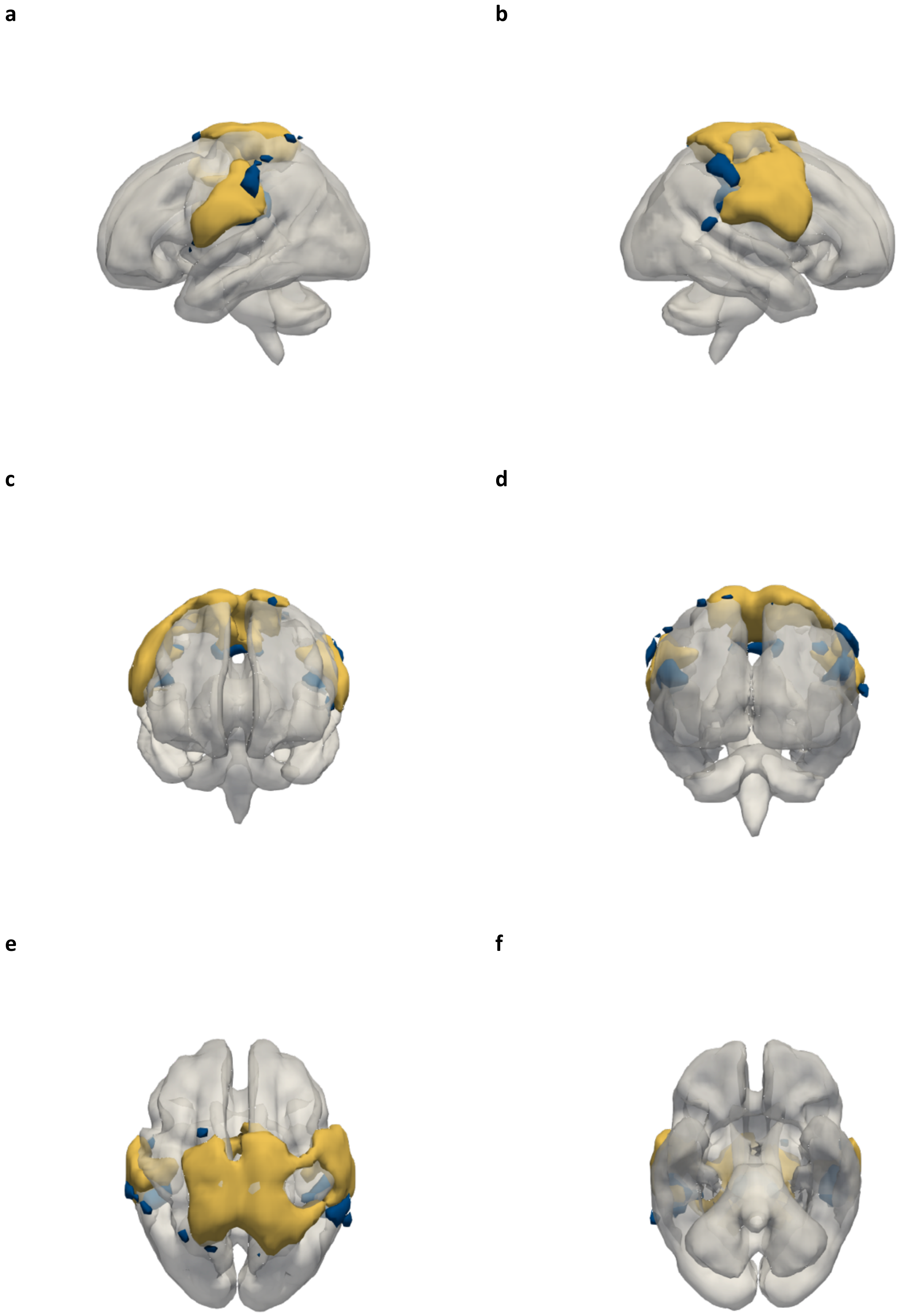

Three-dimensional rendering of the functional grey matter network representing *somatosensory function*. The colours label functional subnetworks separated by neurotransmitter receptor distribution preponderance, here noradrenaline in yellow and glutamate in blue. This forms the basis upon which treatment effect heterogeneity is simulated, with hypothetical treatments selectively effective for lesions disrupting defined receptor territories. Each panel shows the same render from a different spatial perspective: **a**, left; **b**, right; **c**, anterior; **d**, posterior, **e**, superior; **f**, inferior. The underlay is a thresholded white matter template surface in MNI.

Supplementary Figure 42: *Somatosensory* subnetwork by receptome slices

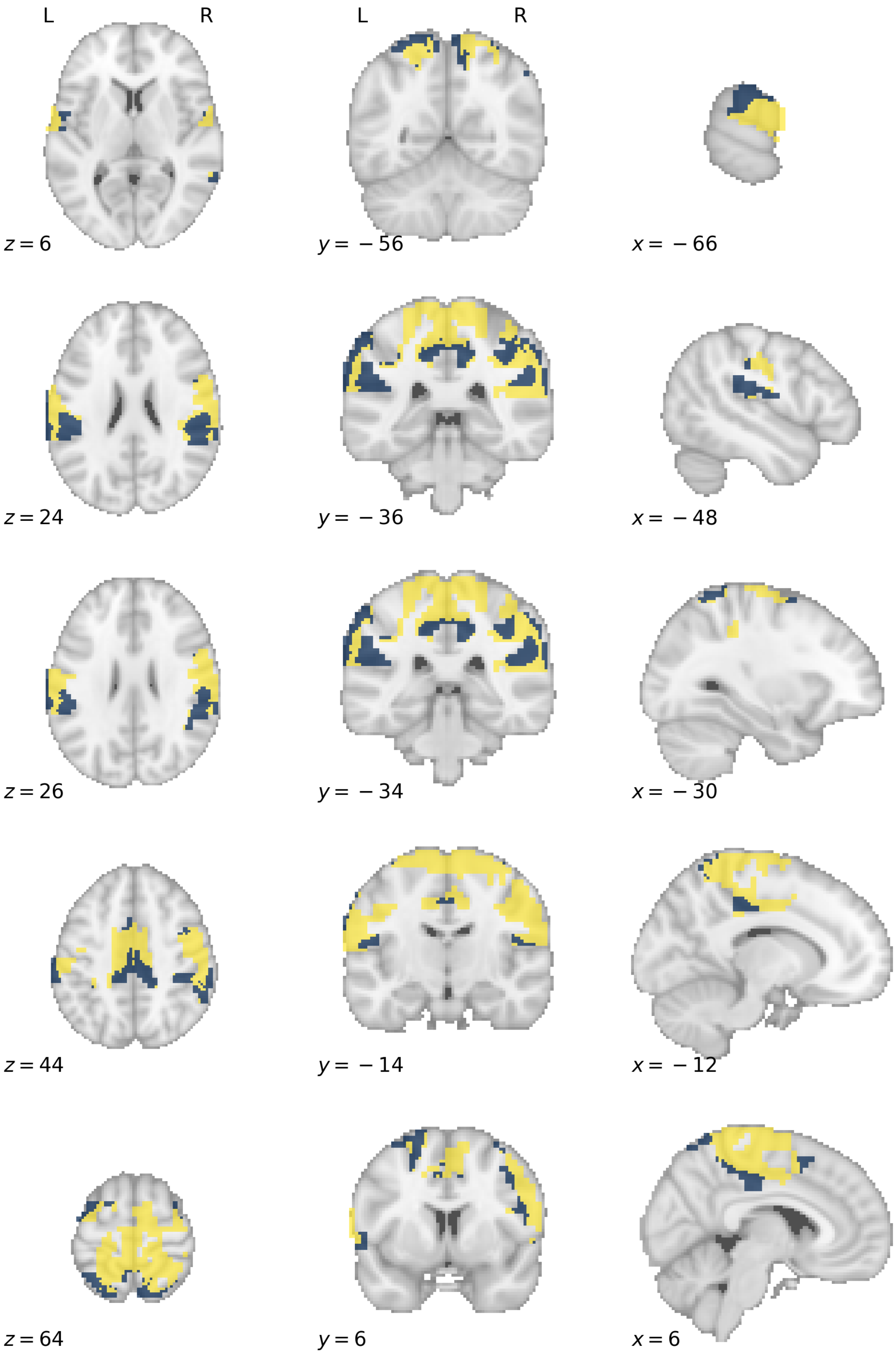

Slice visualization of the functional grey matter network representing *somatosensory function*, overlaid onto the standard MNI152 template. The labelled co-ordinates map to MNI space. The colours label functional subnetworks separated by neurotransmitter receptor distribution preponderance, here noradrenaline in yellow and glutamate in blue. This forms the basis upon which treatment effect heterogeneity is simulated, with hypothetical treatments selectively effective for lesions disrupting defined receptor territories.

Supplementary Figure 43: *Hearing* subnetwork by transcriptome render

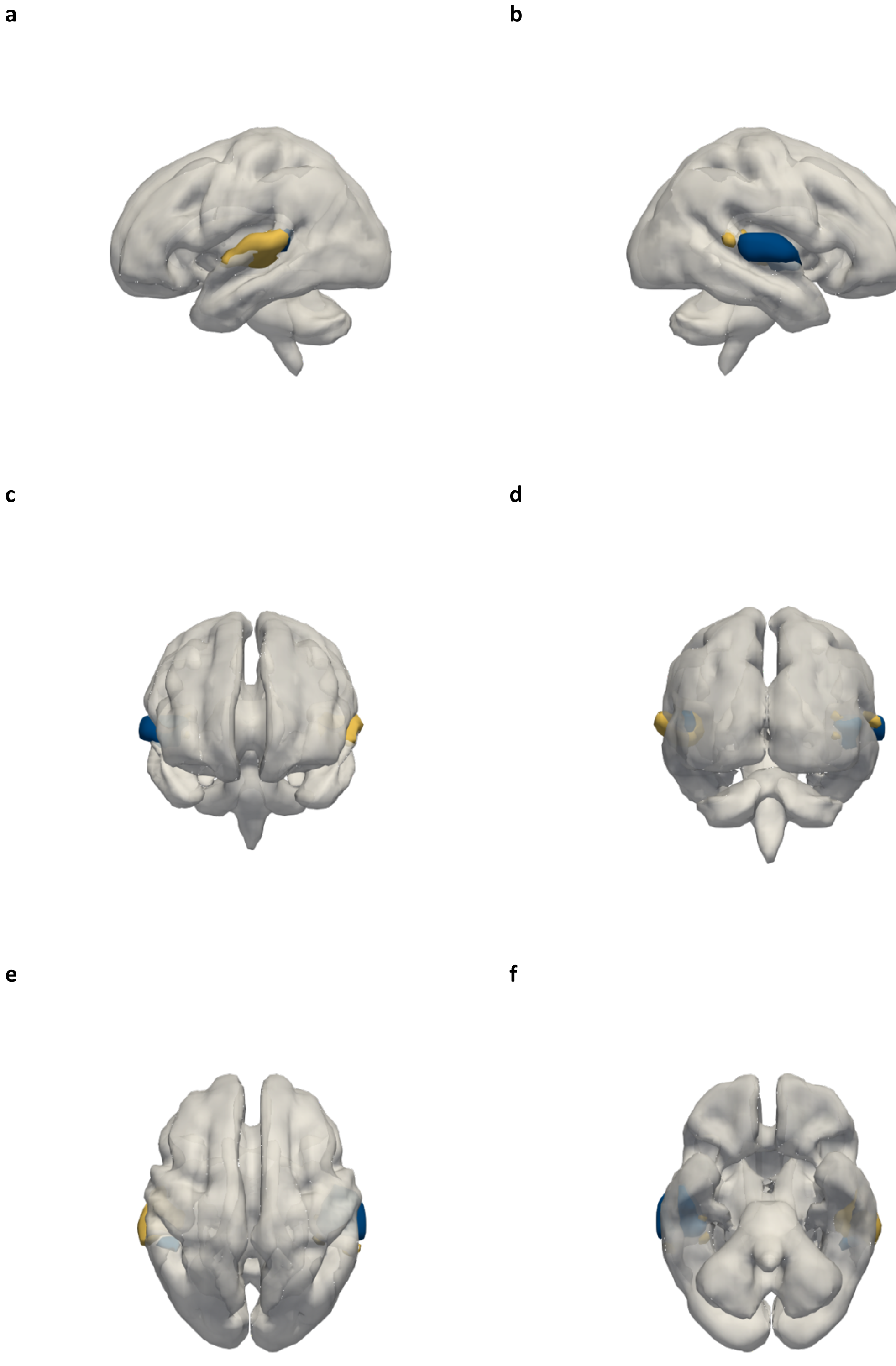

Three-dimensional rendering of the functional grey matter network representing *hearing*. The colours label functional subnetworks separated by microarray gene expression data, here represented in yellow and blue. This forms the basis upon which treatment effect heterogeneity is simulated, with hypothetical treatments selectively effective for lesions disrupting defined subnetworks. Each panel shows the same render from a different spatial perspective: **a**, left; **b**, right; **c**, anterior; **d**, posterior, **e**, superior; **f**, inferior.

Supplementary Figure 44: *Hearing* subnetwork by transcriptome slices

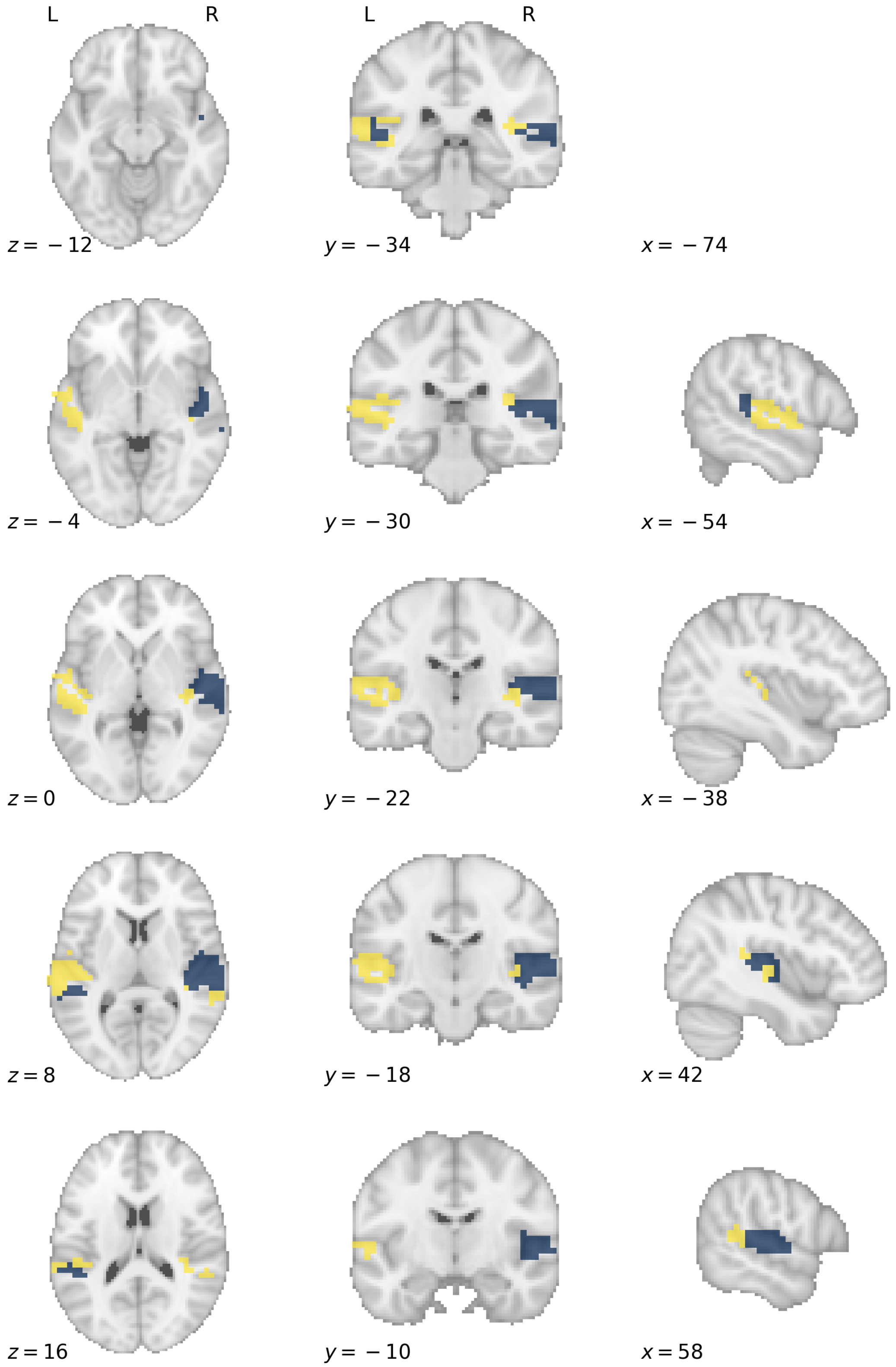

Slice visualization of the functional grey matter network representing *hearing*, overlaid onto the standard MNI152 template. The labelled co-ordinates map to MNI space. The colours label functional subnetworks separated by microarray gene expression, here represented in yellow and blue. This forms the basis upon which treatment effect heterogeneity is simulated, with hypothetical treatments selectively effective for lesions disrupting defined subnetworks.

Supplementary Figure 45: *Language* subnetwork by transcriptome render

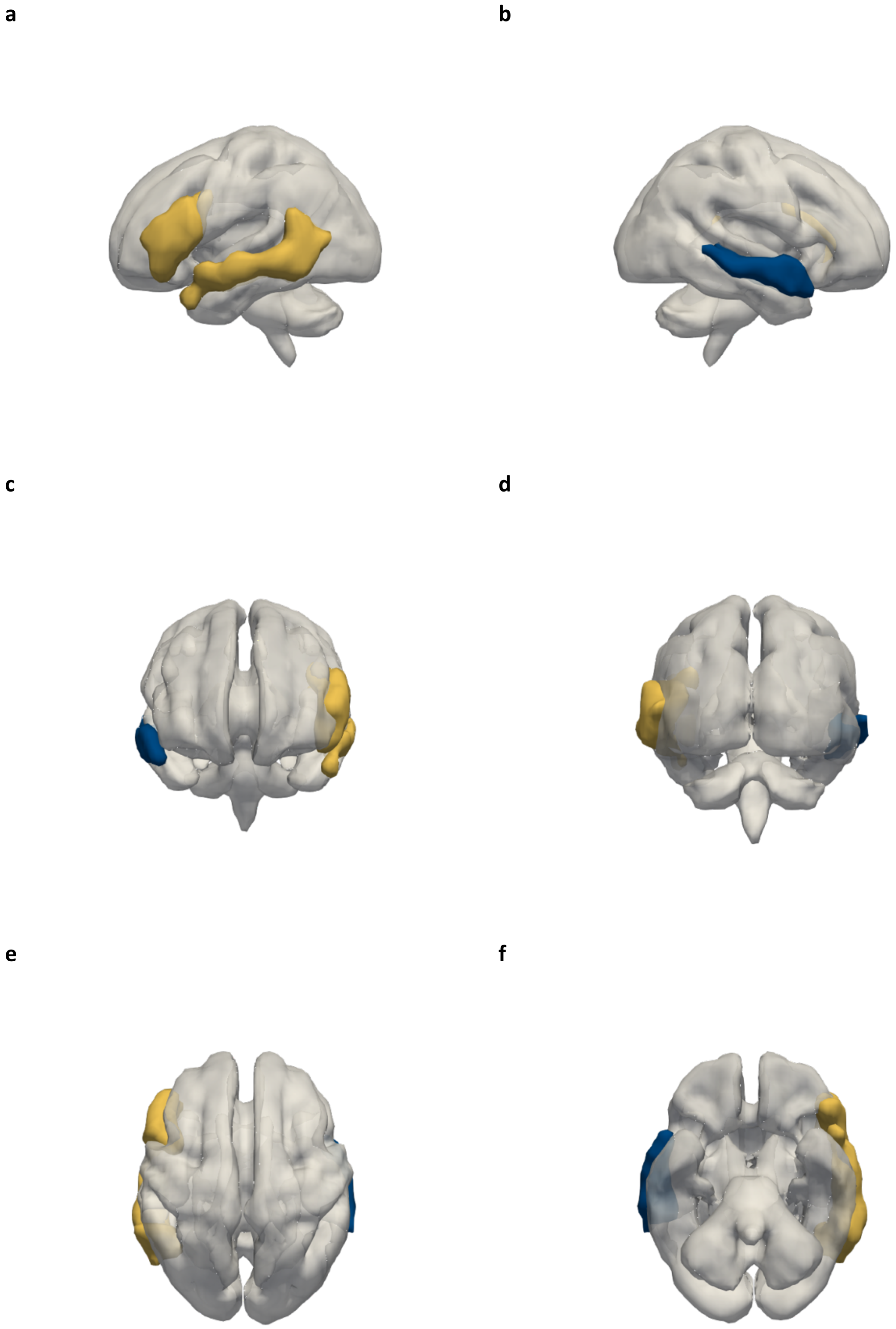

Three-dimensional rendering of the functional grey matter network representing *language*. The colours label functional subnetworks separated by microarray gene expression data, here represented in yellow and blue. This forms the basis upon which treatment effect heterogeneity is simulated, with hypothetical treatments selectively effective for lesions disrupting defined subnetworks. Each panel shows the same render from a different spatial perspective: **a**, left; **b**, right; **c**, anterior; **d**, posterior, **e**, superior; **f**, inferior.

Supplementary Figure 46: *Language* subnetwork by transcriptome slices

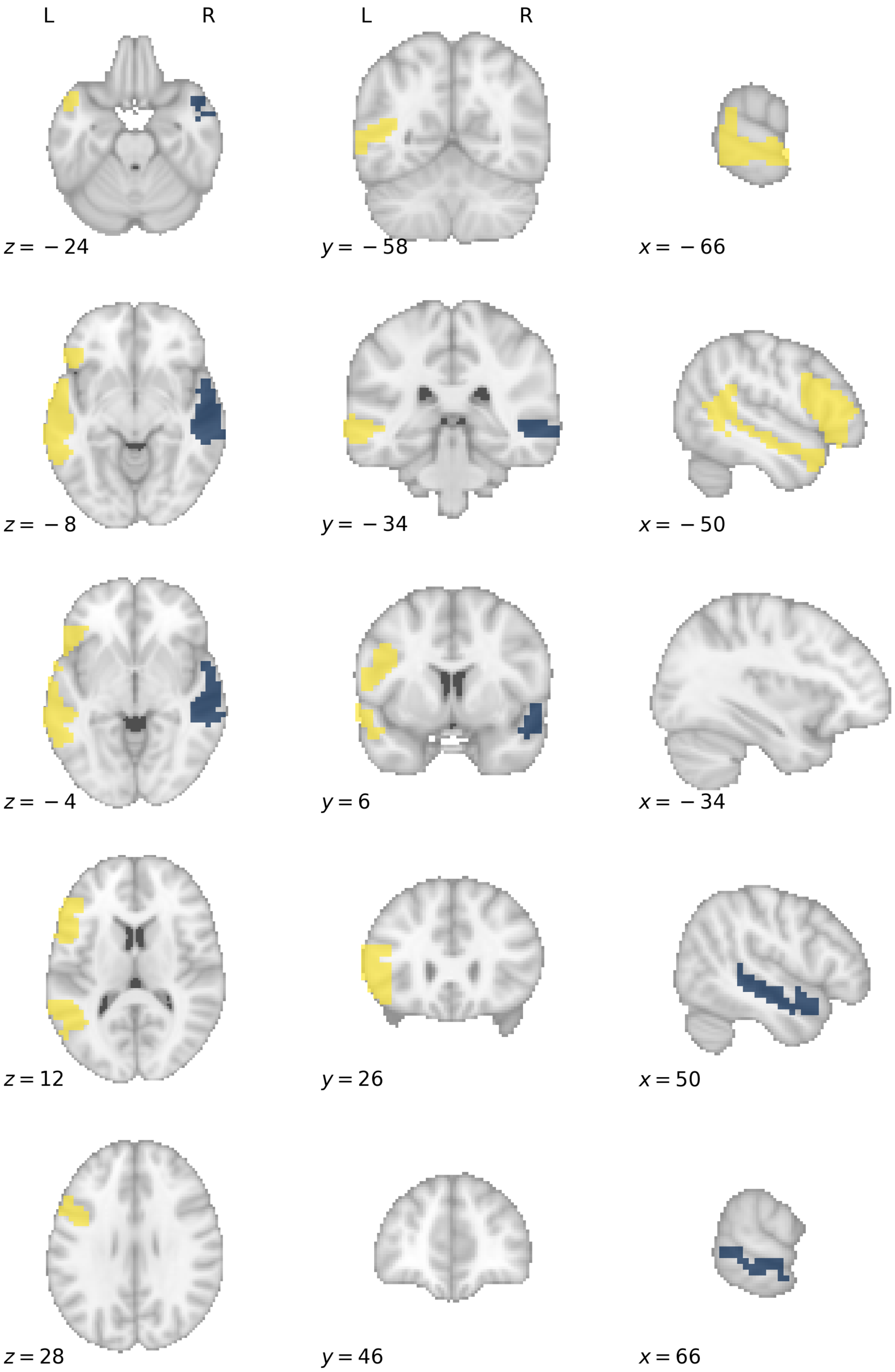

Slice visualization of the functional grey matter network representing *language*, overlaid onto the standard MNI152 template. The labelled co-ordinates map to MNI space. The colours label functional subnetworks separated by microarray gene expression, here represented in yellow and blue. This forms the basis upon which treatment effect heterogeneity is simulated, with hypothetical treatments selectively effective for lesions disrupting defined subnetworks.

Supplementary Figure 47: *Introspection* subnetwork by transcriptome render

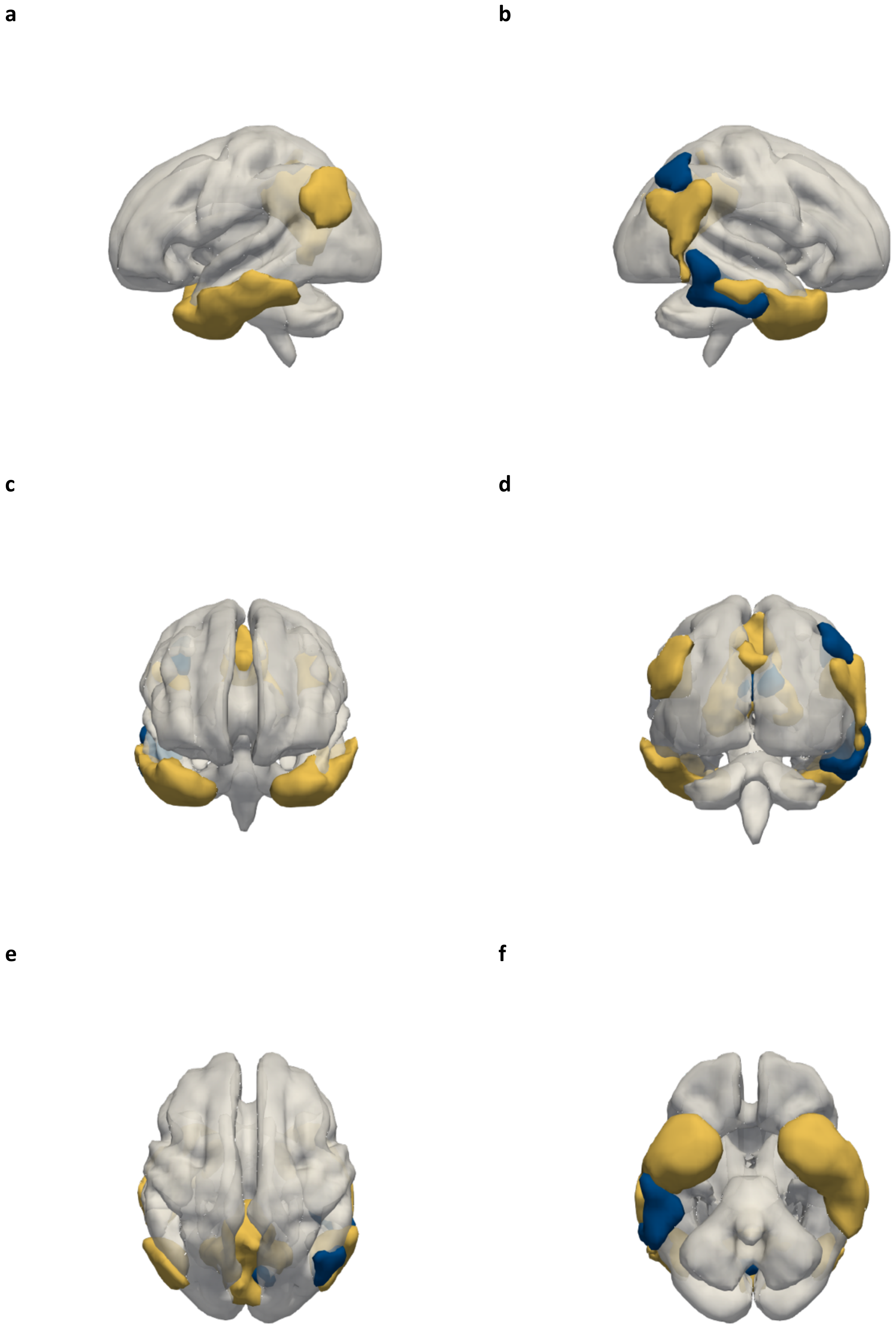

Three-dimensional rendering of the functional grey matter network representing *introspection*. The colours label functional subnetworks separated by microarray gene expression data, here represented in yellow and blue. This forms the basis upon which treatment effect heterogeneity is simulated, with hypothetical treatments selectively effective for lesions disrupting defined subnetworks. Each panel shows the same render from a different spatial perspective: **a**, left; **b**, right; **c**, anterior; **d**, posterior, **e**, superior; **f**, inferior.

Supplementary Figure 48: *Introspection* subnetwork by transcriptome slices

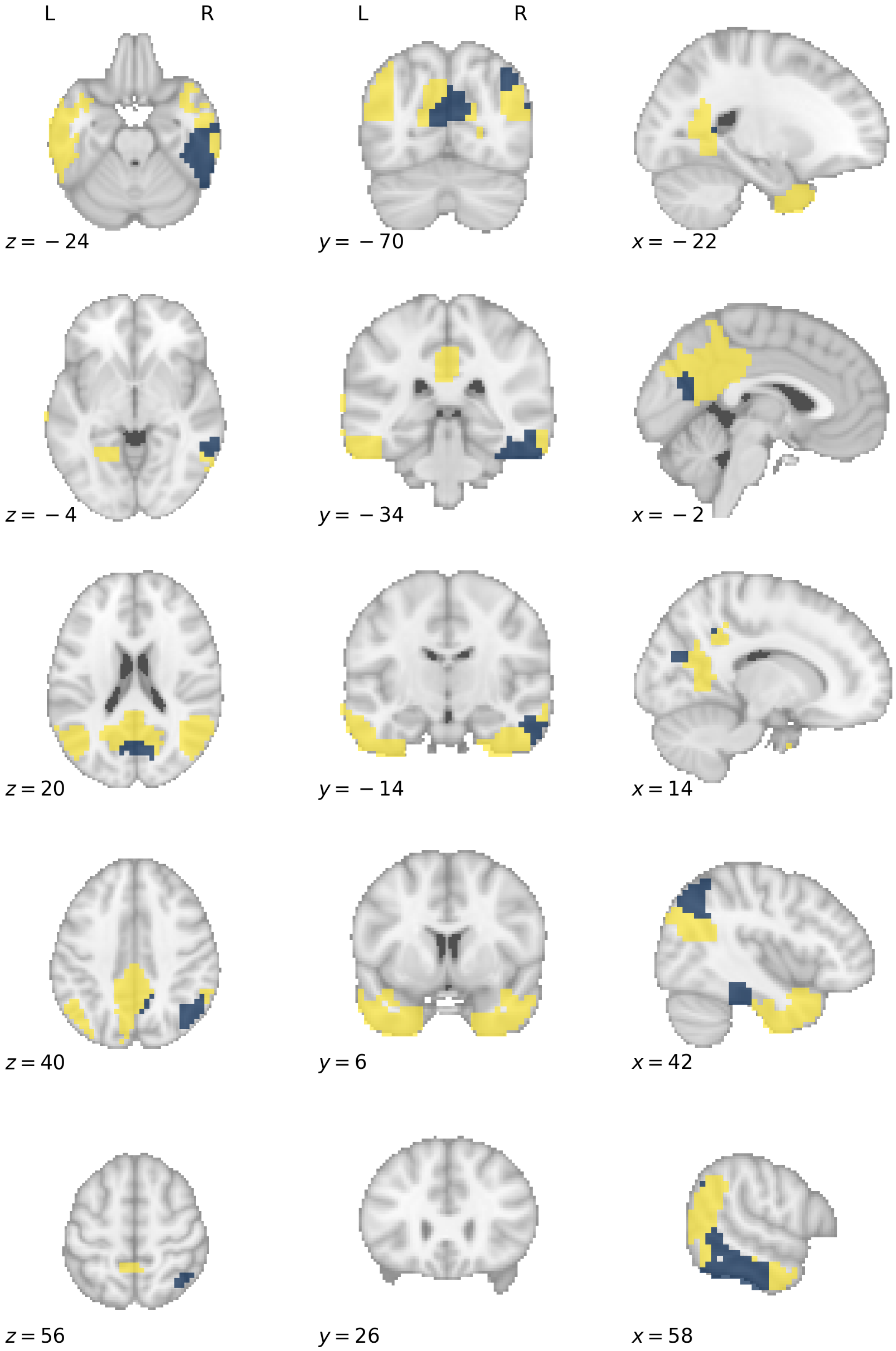

Slice visualization of the functional grey matter network representing *introspection*, overlaid onto the standard MNI152 template. The labelled co-ordinates map to MNI space. The colours label functional subnetworks separated by microarray gene expression, here represented in yellow and blue. This forms the basis upon which treatment effect heterogeneity is simulated, with hypothetical treatments selectively effective for lesions disrupting defined subnetworks.

Supplementary Figure 49: *Cognition* subnetwork by transcriptome render

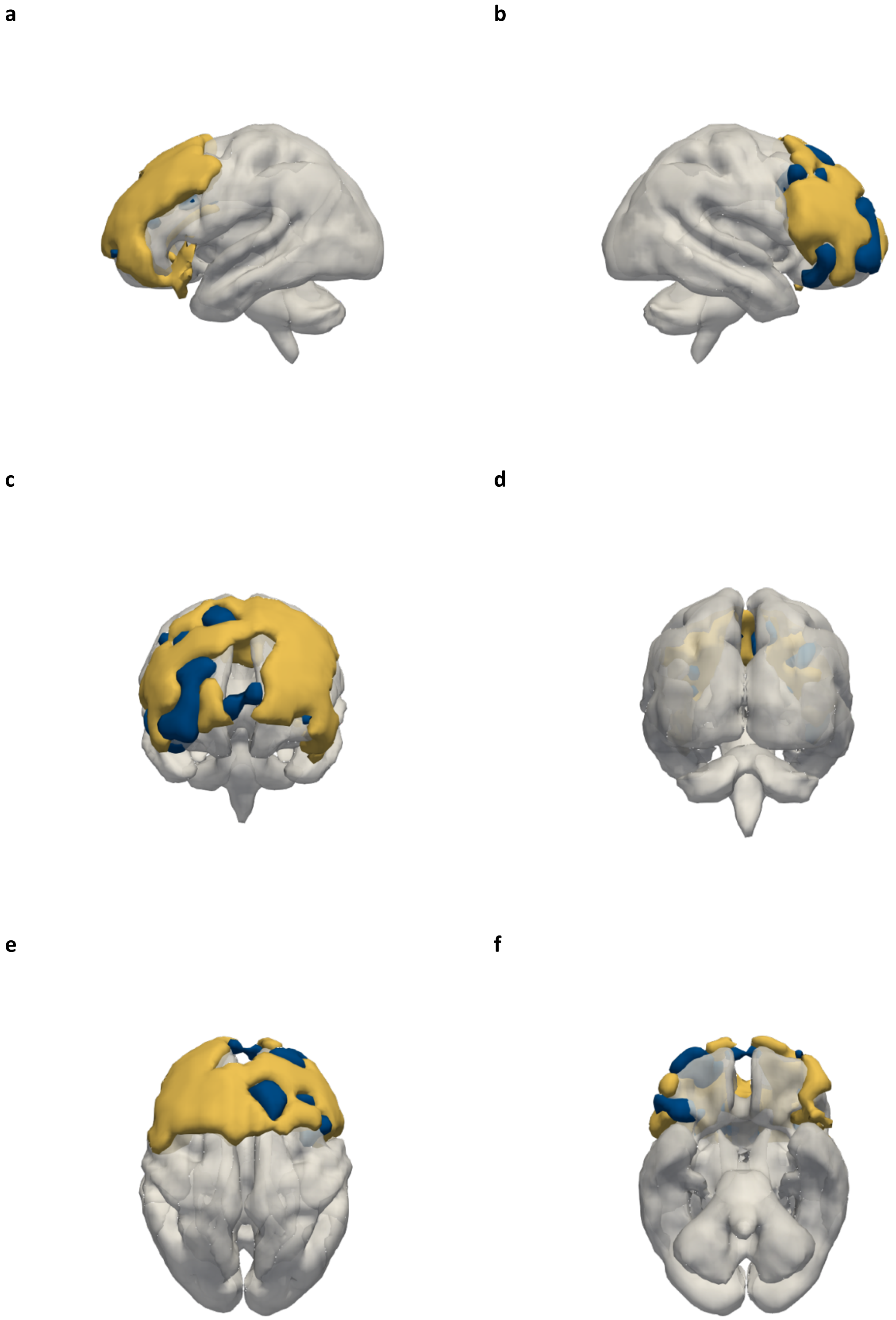

Three-dimensional rendering of the functional grey matter network representing *cognition*. The colours label functional subnetworks separated by microarray gene expression data, here represented in yellow and blue. This forms the basis upon which treatment effect heterogeneity is simulated, with hypothetical treatments selectively effective for lesions disrupting defined subnetworks. Each panel shows the same render from a different spatial perspective: **a**, left; **b**, right; **c**, anterior; **d**, posterior, **e**, superior; **f**, inferior.

Supplementary Figure 50: *Cognition* subnetwork by transcriptome slices

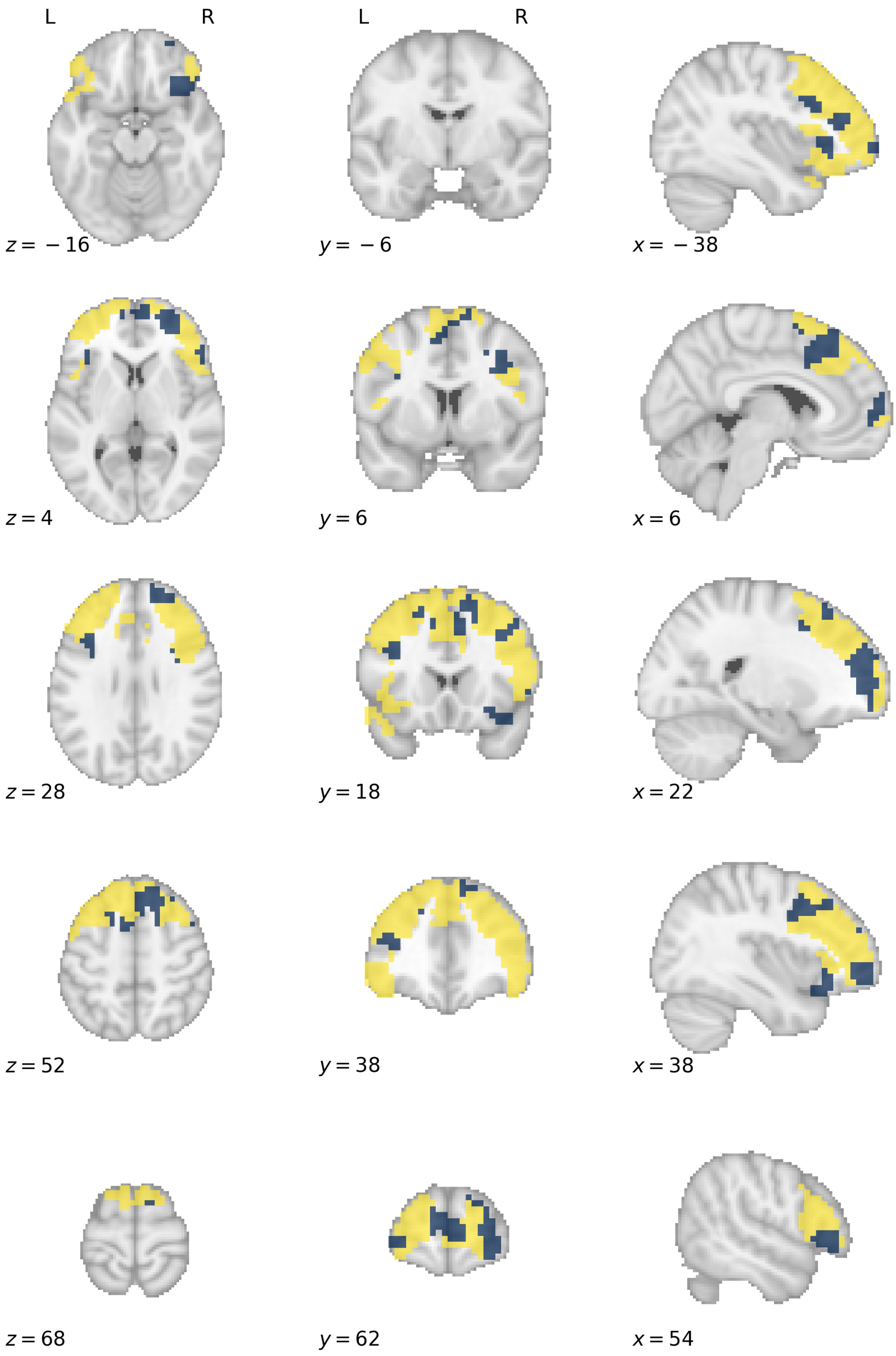

Slice visualization of the functional grey matter network representing *cognition*, overlaid onto the standard MNI152 template. The labelled co-ordinates map to MNI space. The colours label functional subnetworks separated by microarray gene expression, here represented in yellow and blue. This forms the basis upon which treatment effect heterogeneity is simulated, with hypothetical treatments selectively effective for lesions disrupting defined subnetworks.

Supplementary Figure 51: *Mood* subnetwork by transcriptome render

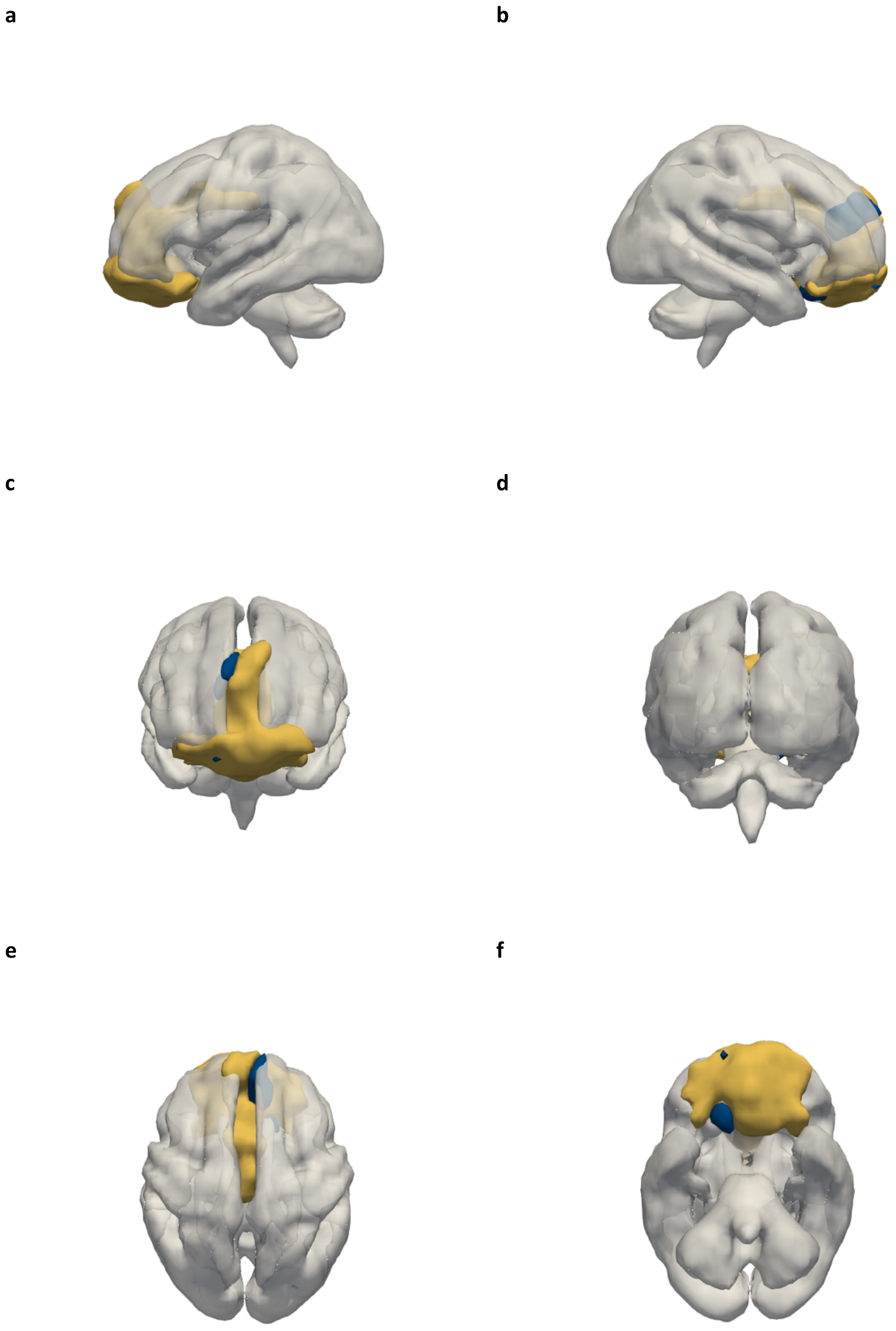

Three-dimensional rendering of the functional grey matter network representing *mood*. The colours label functional subnetworks separated by microarray gene expression data, here represented in yellow and blue. This forms the basis upon which treatment effect heterogeneity is simulated, with hypothetical treatments selectively effective for lesions disrupting defined subnetworks. Each panel shows the same render from a different spatial perspective: **a**, left; **b**, right; **c**, anterior; **d**, posterior, **e**, superior; **f**, inferior.

Supplementary Figure 52: *Mood* subnetwork by transcriptome slices

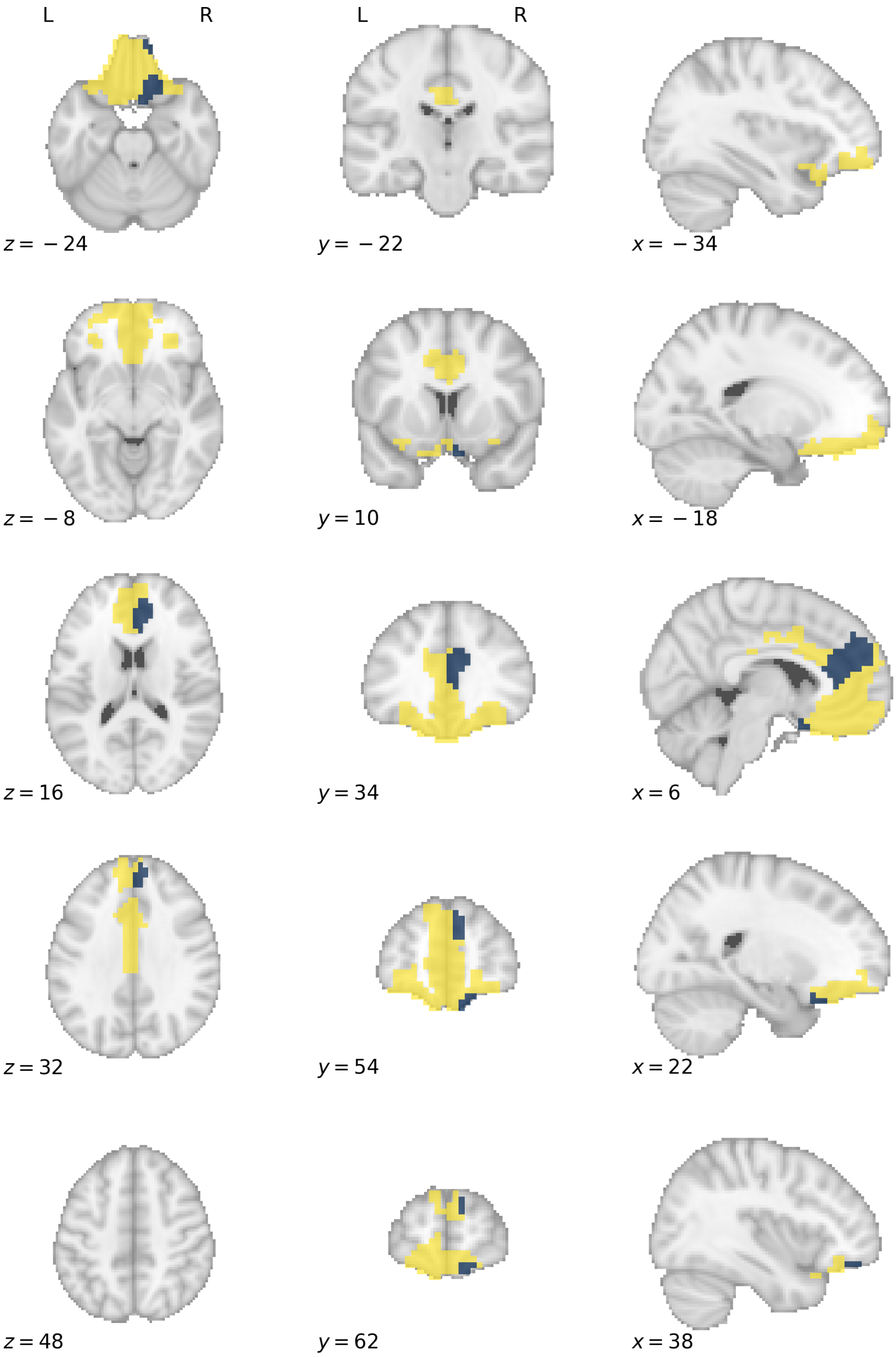

Slice visualization of the functional grey matter network representing *mood*, overlaid onto the standard MNI152 template. The labelled co-ordinates map to MNI space. The colours label functional subnetworks separated by microarray gene expression, here represented in yellow and blue. This forms the basis upon which treatment effect heterogeneity is simulated, with hypothetical treatments selectively effective for lesions disrupting defined subnetworks.

Supplementary Figure 53: *Memory* subnetwork by transcriptome render

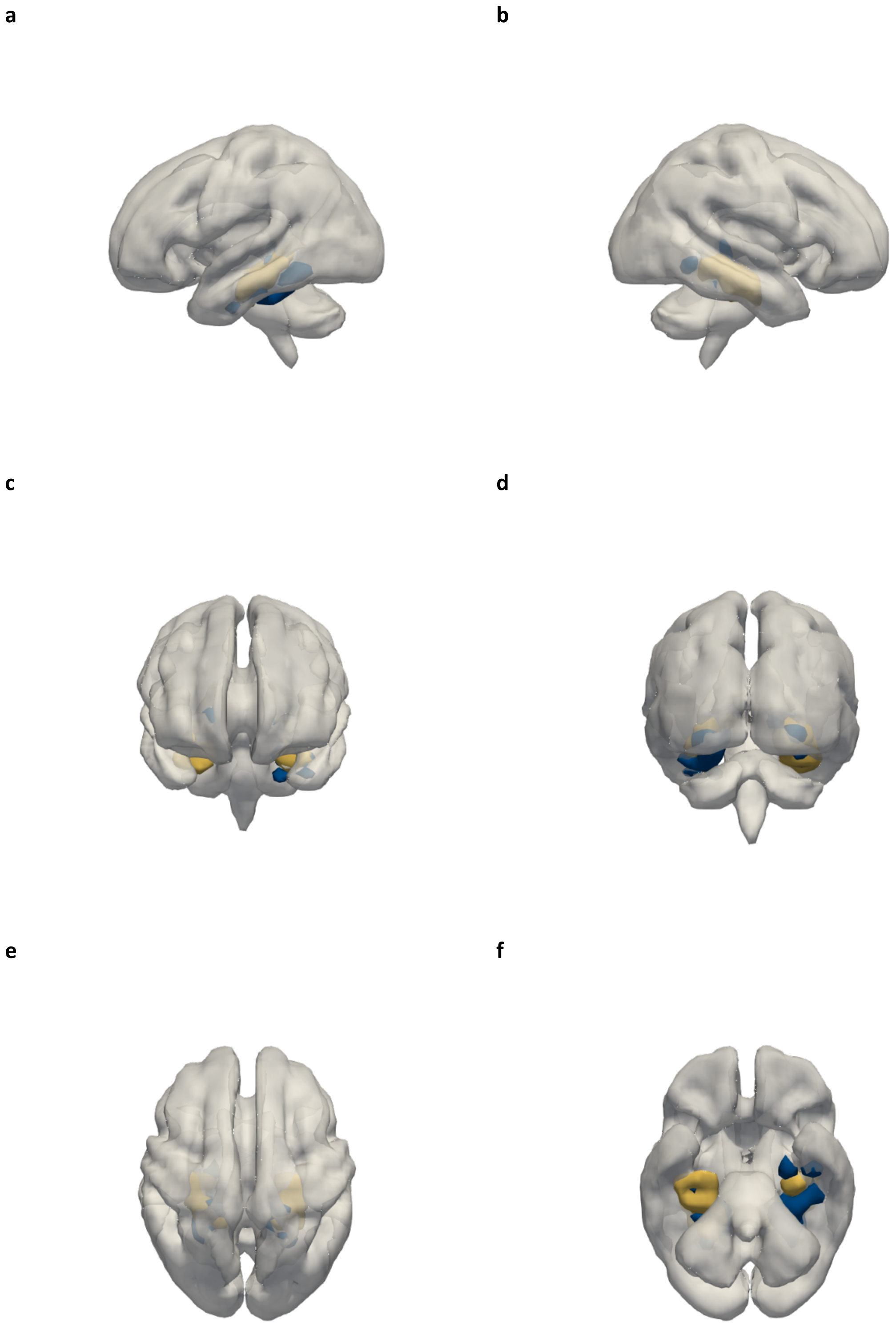

Three-dimensional rendering of the functional grey matter network representing *memory*. The colours label functional subnetworks separated by microarray gene expression data, here represented in yellow and blue. This forms the basis upon which treatment effect heterogeneity is simulated, with hypothetical treatments selectively effective for lesions disrupting defined subnetworks. Each panel shows the same render from a different spatial perspective: **a**, left; **b**, right; **c**, anterior; **d**, posterior, **e**, superior; **f**, inferior.

Supplementary Figure 54: *Memory* subnetwork by transcriptome slices

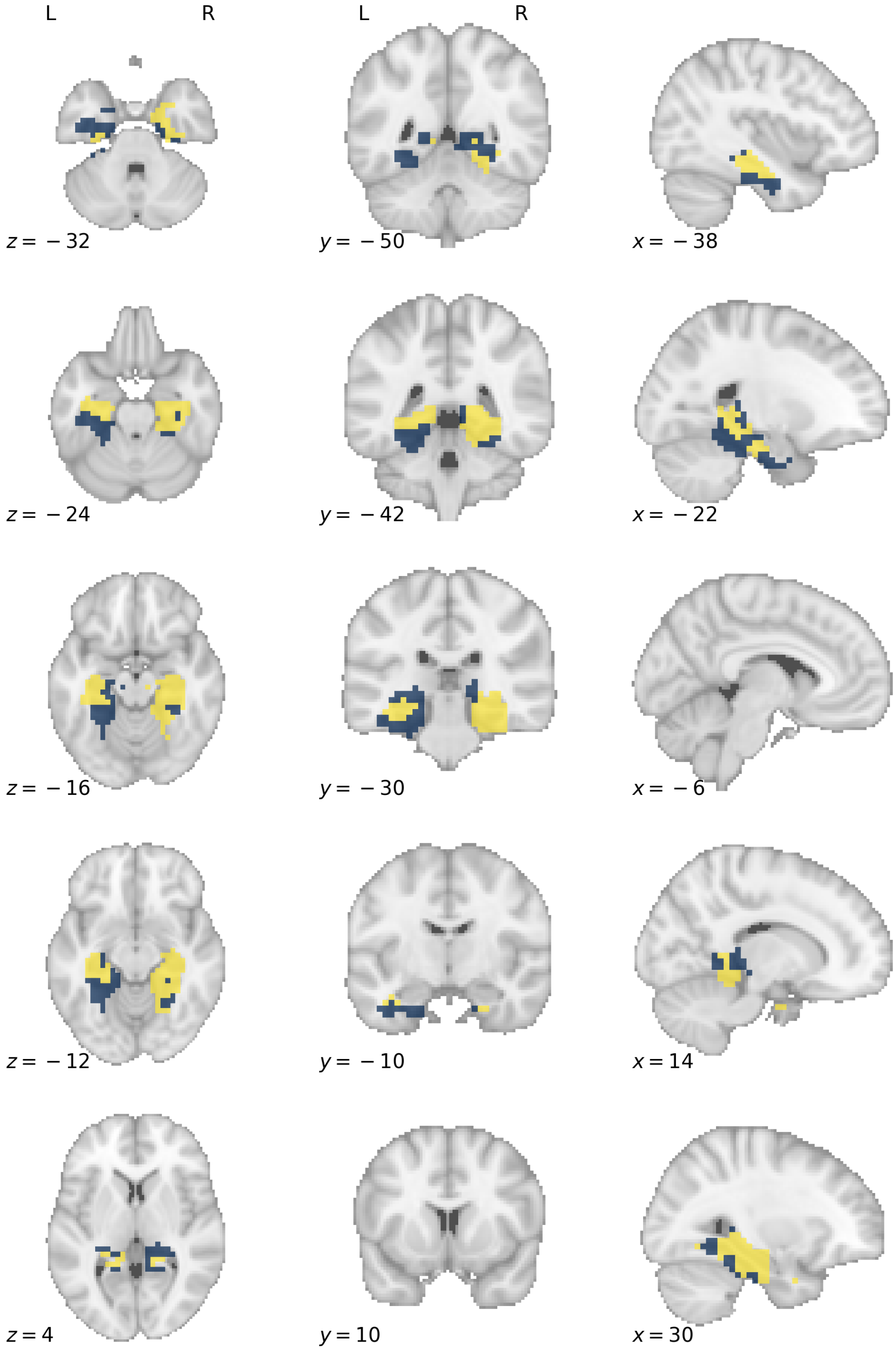

Slice visualization of the functional grey matter network representing *memory*, overlaid onto the standard MNI152 template. The labelled co-ordinates map to MNI space. The colours label functional subnetworks separated by microarray gene expression, here represented in yellow and blue. This forms the basis upon which treatment effect heterogeneity is simulated, with hypothetical treatments selectively effective for lesions disrupting defined subnetworks.

Supplementary Figure 55: *Aversion* subnetwork by transcriptome render

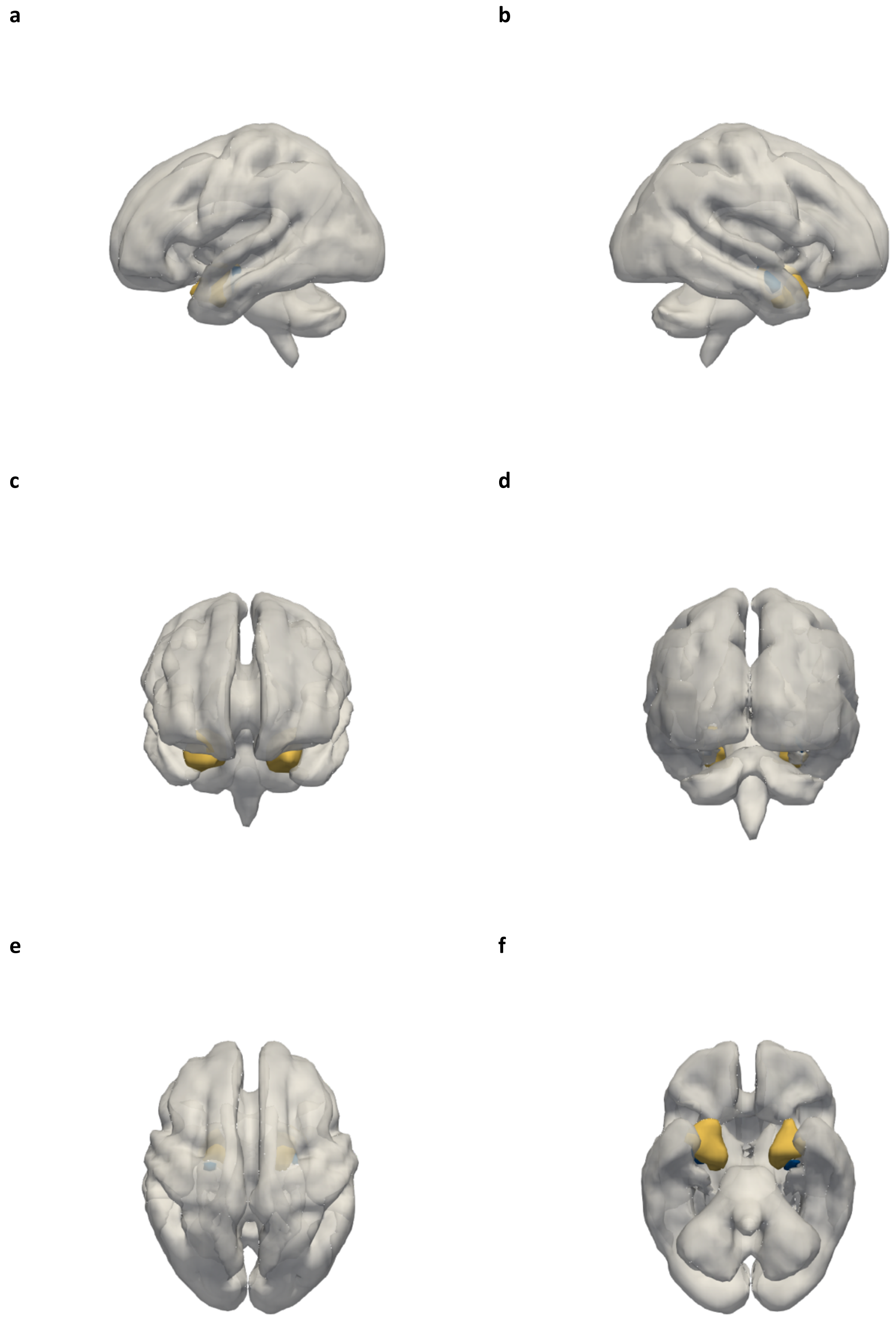

Three-dimensional rendering of the functional grey matter network representing *aversion*. The colours label functional subnetworks separated by microarray gene expression data, here represented in yellow and blue. This forms the basis upon which treatment effect heterogeneity is simulated, with hypothetical treatments selectively effective for lesions disrupting defined subnetworks. Each panel shows the same render from a different spatial perspective: **a**, left; **b**, right; **c**, anterior; **d**, posterior, **e**, superior; **f**, inferior.

Supplementary Figure 56: *Aversion* subnetwork by transcriptome slices

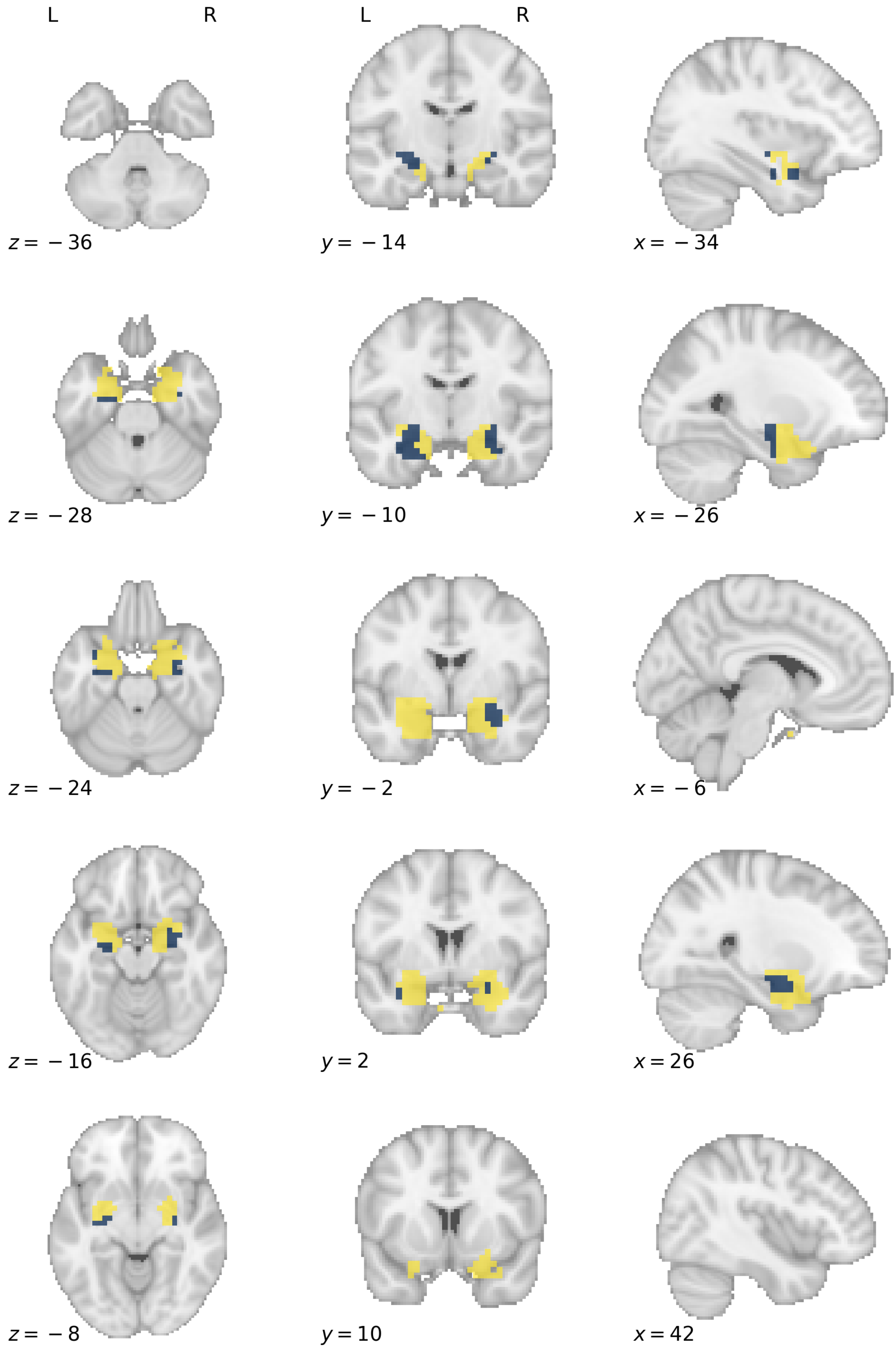

Slice visualization of the functional grey matter network representing *aversion*, overlaid onto the standard MNI152 template. The labelled co-ordinates map to MNI space. The colours label functional subnetworks separated by microarray gene expression, here represented in yellow and blue. This forms the basis upon which treatment effect heterogeneity is simulated, with hypothetical treatments selectively effective for lesions disrupting defined subnetworks.

Supplementary Figure 57: *Coordination* subnetwork by transcriptome render

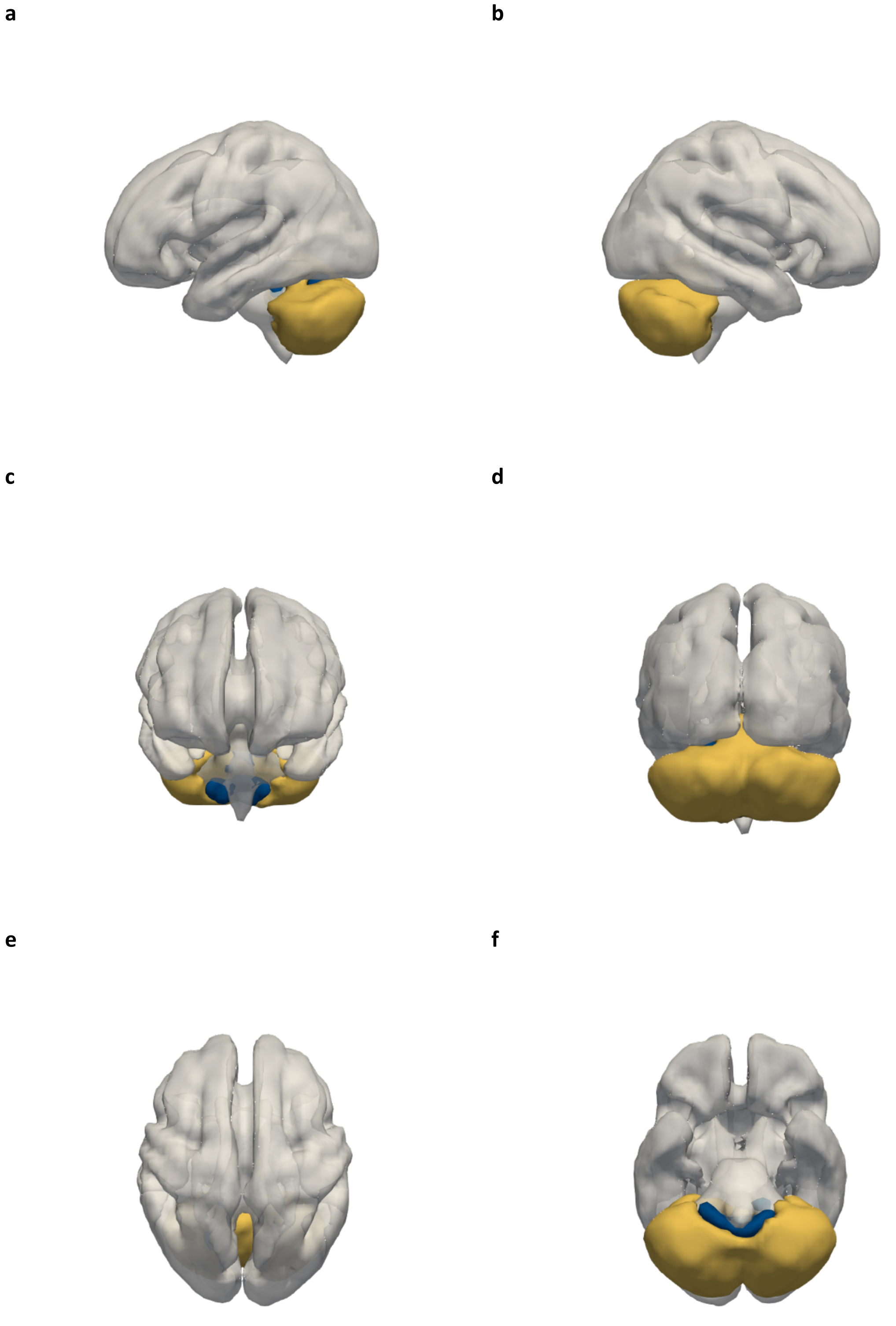

Three-dimensional rendering of the functional grey matter network representing *coordination*. The colours label functional subnetworks separated by microarray gene expression data, here represented in yellow and blue. This forms the basis upon which treatment effect heterogeneity is simulated, with hypothetical treatments selectively effective for lesions disrupting defined subnetworks. Each panel shows the same render from a different spatial perspective: **a**, left; **b**, right; **c**, anterior; **d**, posterior, **e**, superior; **f**, inferior.

Supplementary Figure 58: *Coordination* subnetwork by transcriptome slices

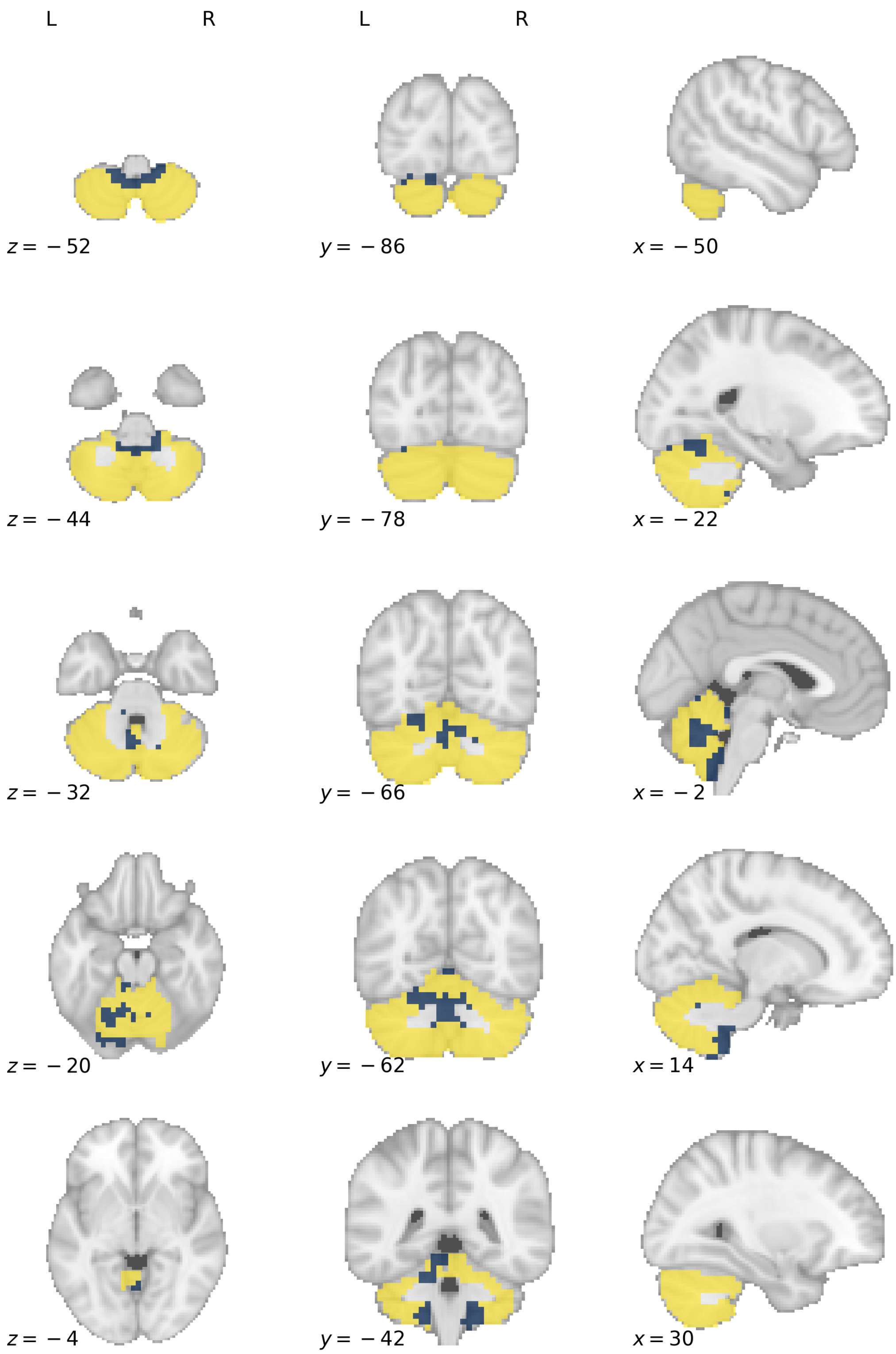

Slice visualization of the functional grey matter network representing *coordination*, overlaid onto the standard MNI152 template. The labelled co-ordinates map to MNI space. The colours label functional subnetworks separated by microarray gene expression, here represented in yellow and blue. This forms the basis upon which treatment effect heterogeneity is simulated, with hypothetical treatments selectively effective for lesions disrupting defined subnetworks.

Supplementary Figure 59: *Interoception* subnetwork by transcriptome render

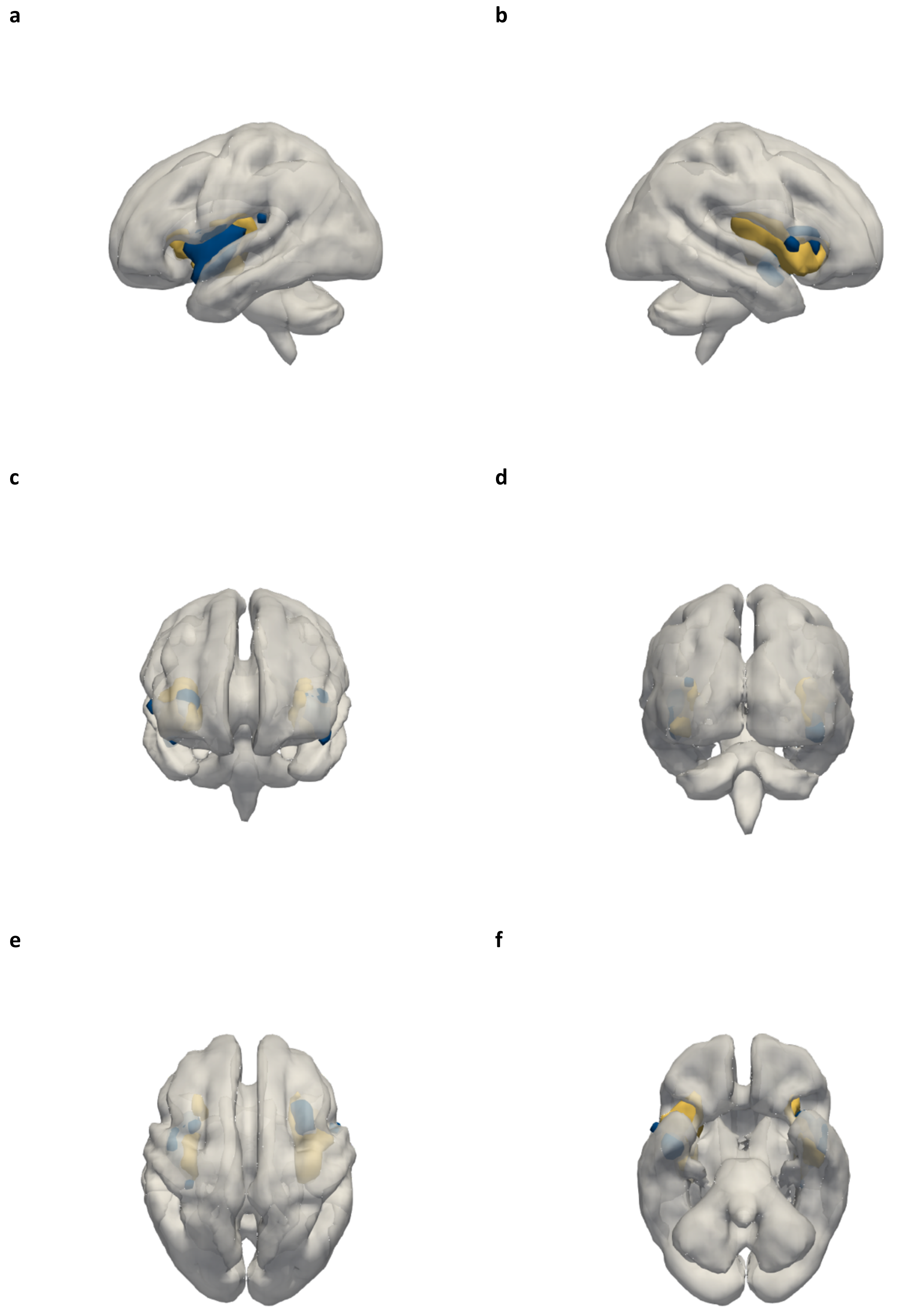

Three-dimensional rendering of the functional grey matter network representing *interoception*. The colours label functional subnetworks separated by microarray gene expression data, here represented in yellow and blue. This forms the basis upon which treatment effect heterogeneity is simulated, with hypothetical treatments selectively effective for lesions disrupting defined subnetworks. Each panel shows the same render from a different spatial perspective: **a**, left; **b**, right; **c**, anterior; **d**, posterior, **e**, superior; **f**, inferior.

Supplementary Figure 60: *Interoception* subnetwork by transcriptome slices

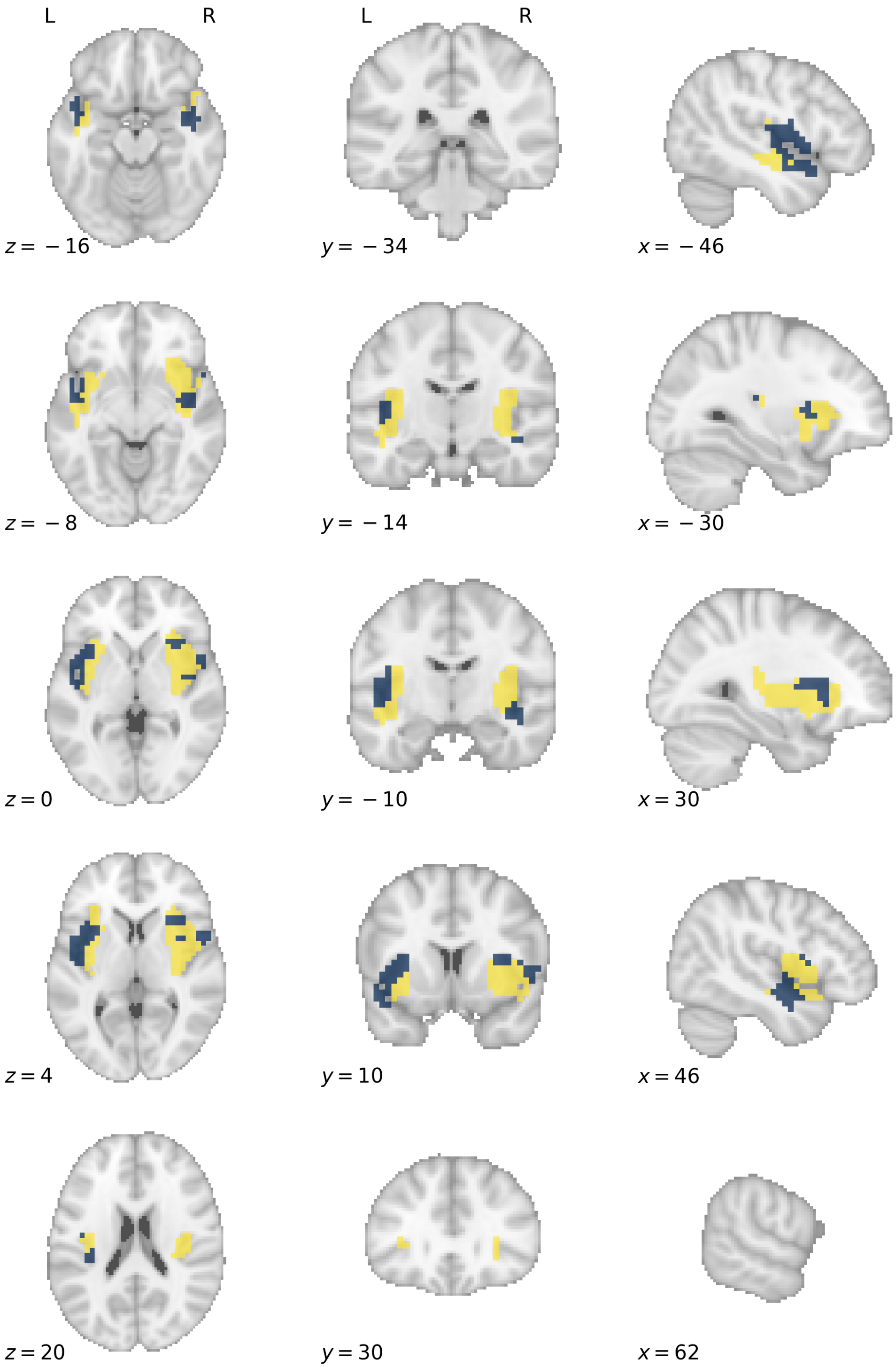

Slice visualization of the functional grey matter network representing *interoception*, overlaid onto the standard MNI152 template. The labelled co-ordinates map to MNI space. The colours label functional subnetworks separated by microarray gene expression, here represented in yellow and blue. This forms the basis upon which treatment effect heterogeneity is simulated, with hypothetical treatments selectively effective for lesions disrupting defined subnetworks.

Supplementary Figure 61: *Sleep* subnetwork by transcriptome render

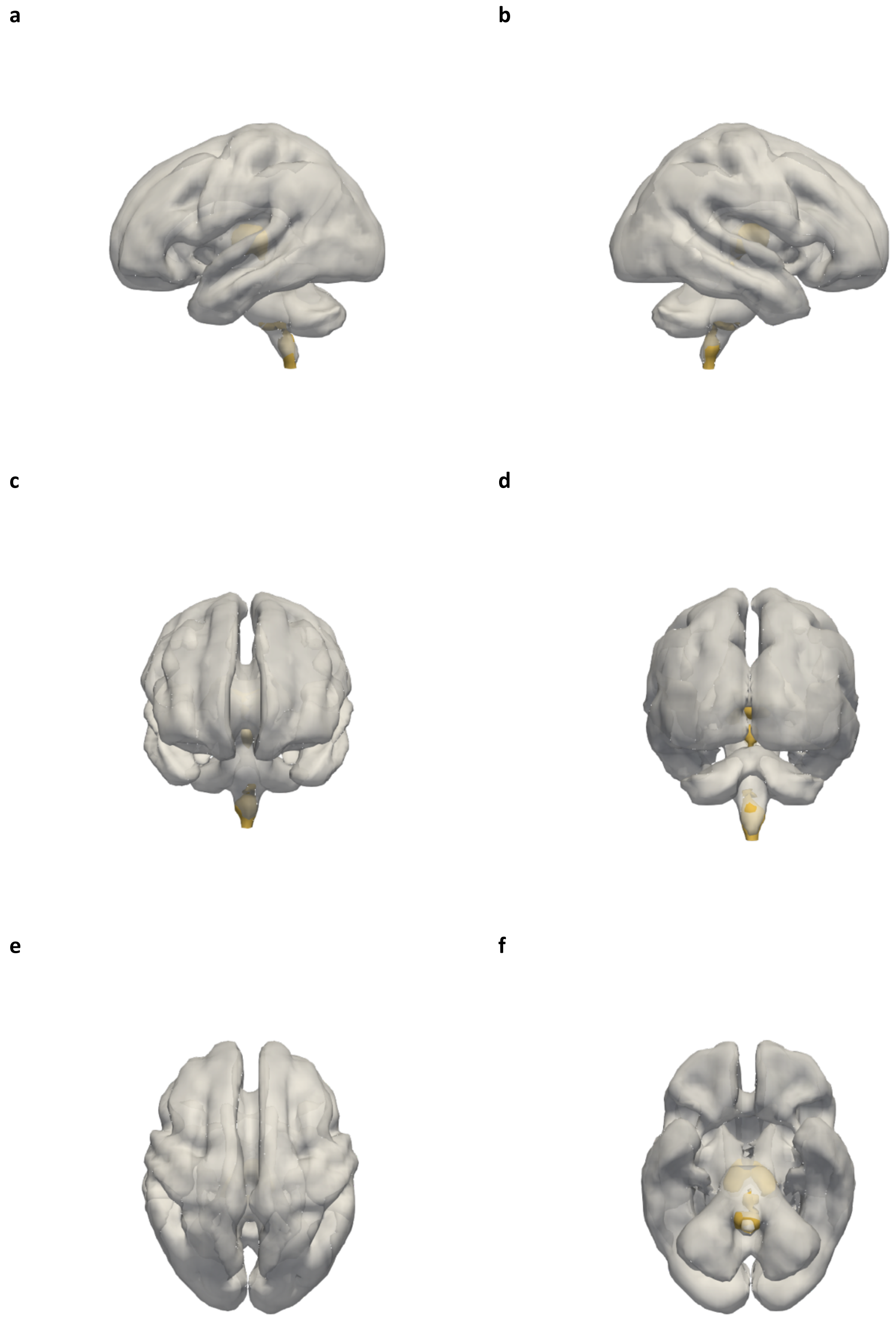

Three-dimensional rendering of the functional grey matter network representing *sleep*. The colours label functional subnetworks separated by microarray gene expression data, here represented in yellow and blue. This forms the basis upon which treatment effect heterogeneity is simulated, with hypothetical treatments selectively effective for lesions disrupting defined subnetworks. Each panel shows the same render from a different spatial perspective: **a**, left; **b**, right; **c**, anterior; **d**, posterior, **e**, superior; **f**, inferior.

Supplementary Figure 62: *Sleep* subnetwork by transcriptome slices

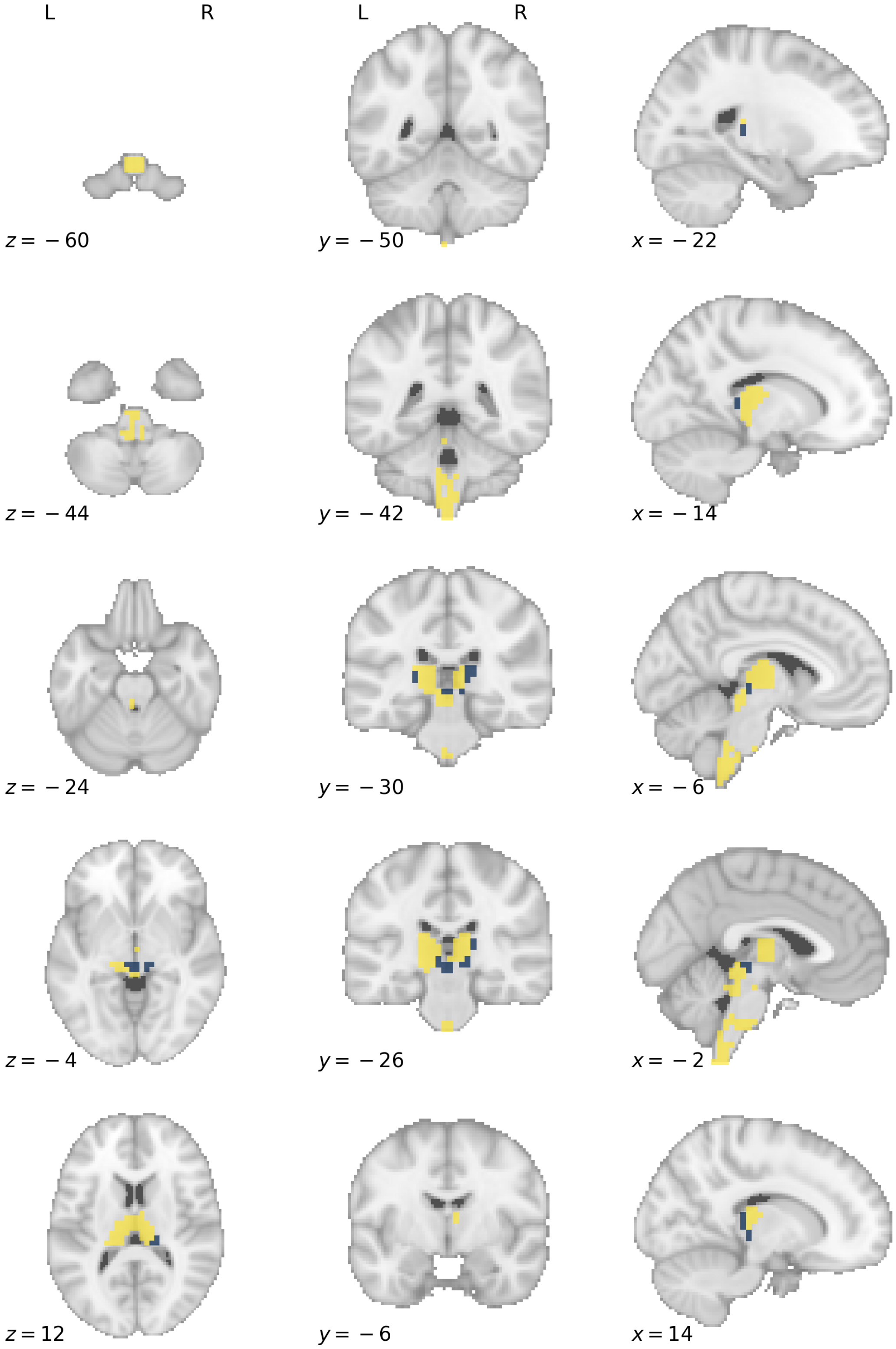

Slice visualization of the functional grey matter network representing *sleep*, overlaid onto the standard MNI152 template. The labelled co-ordinates map to MNI space. The colours label functional subnetworks separated by microarray gene expression, here represented in yellow and blue. This forms the basis upon which treatment effect heterogeneity is simulated, with hypothetical treatments selectively effective for lesions disrupting defined subnetworks.

Supplementary Figure 63: *Reward* subnetwork by transcriptome render

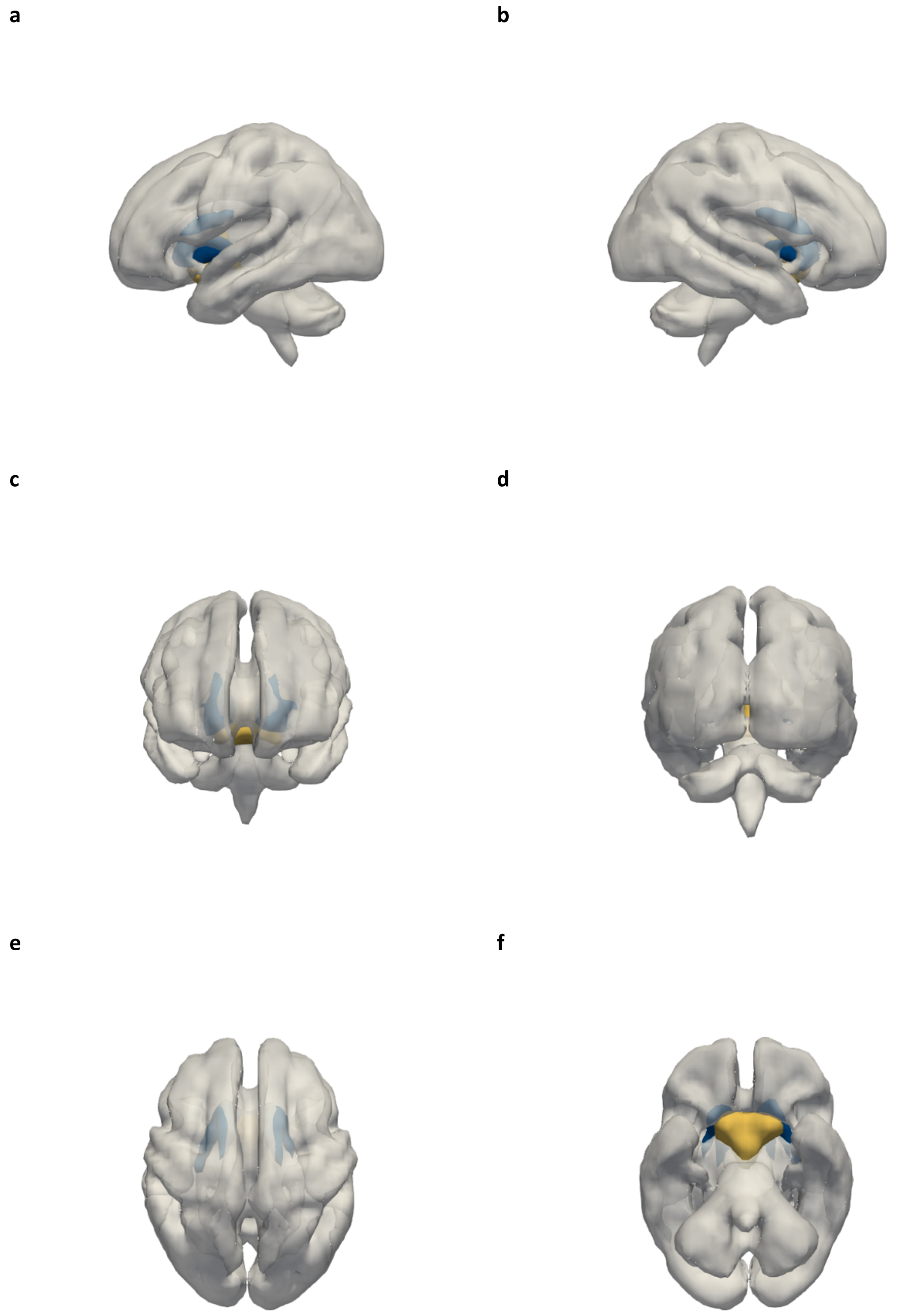

Three-dimensional rendering of the functional grey matter network representing *reward*. The colours label functional subnetworks separated by microarray gene expression data, here represented in yellow and blue. This forms the basis upon which treatment effect heterogeneity is simulated, with hypothetical treatments selectively effective for lesions disrupting defined subnetworks. Each panel shows the same render from a different spatial perspective: **a**, left; **b**, right; **c**, anterior; **d**, posterior, **e**, superior; **f**, inferior.

Supplementary Figure 64: *Reward* subnetwork by transcriptome slices

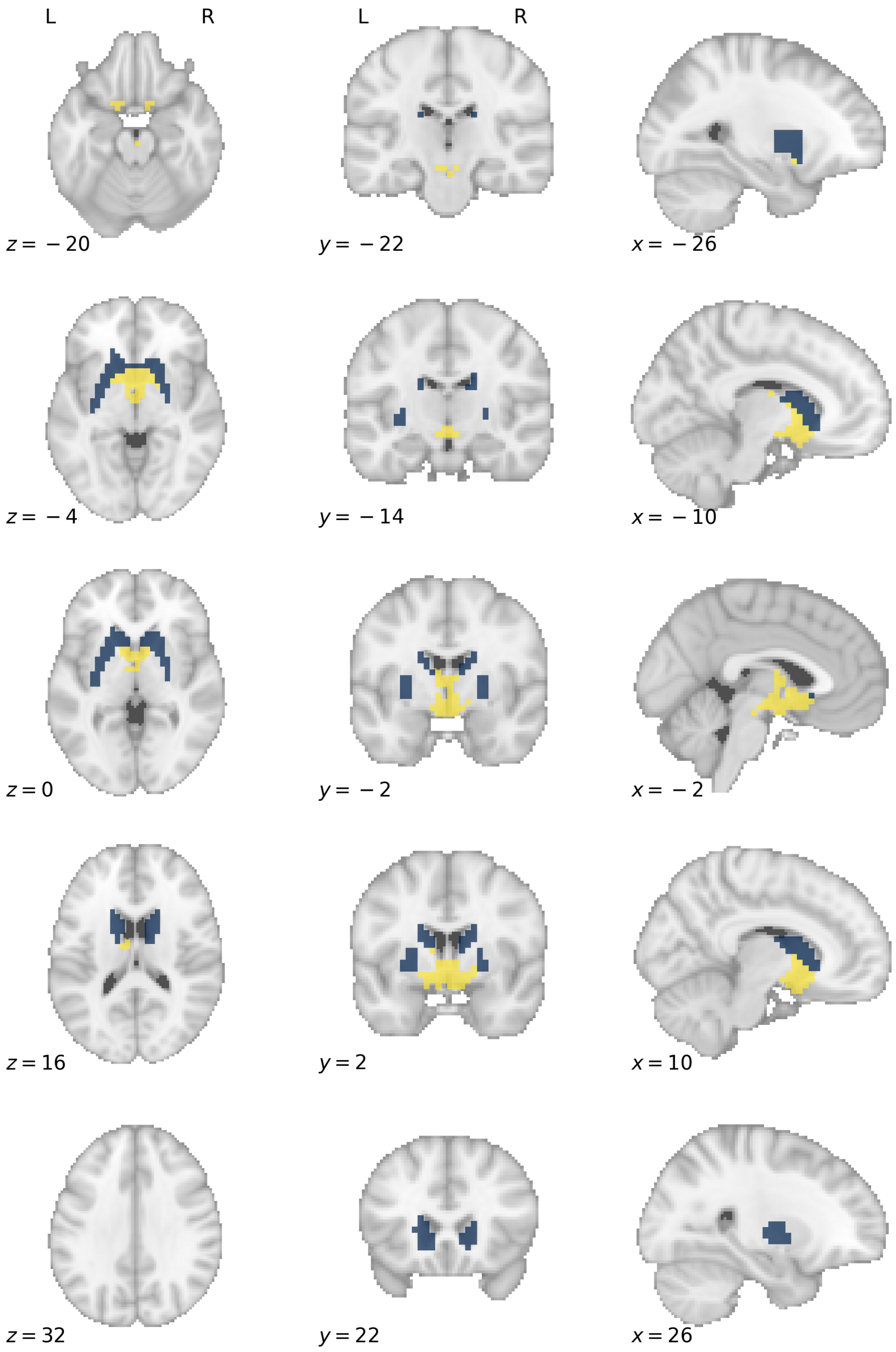

Slice visualization of the functional grey matter network representing *reward*, overlaid onto the standard MNI152 template. The labelled co-ordinates map to MNI space. The colours label functional subnetworks separated by microarray gene expression, here represented in yellow and blue. This forms the basis upon which treatment effect heterogeneity is simulated, with hypothetical treatments selectively effective for lesions disrupting defined subnetworks.

Supplementary Figure 65: *Visual Recognition* subnetwork by transcriptome render

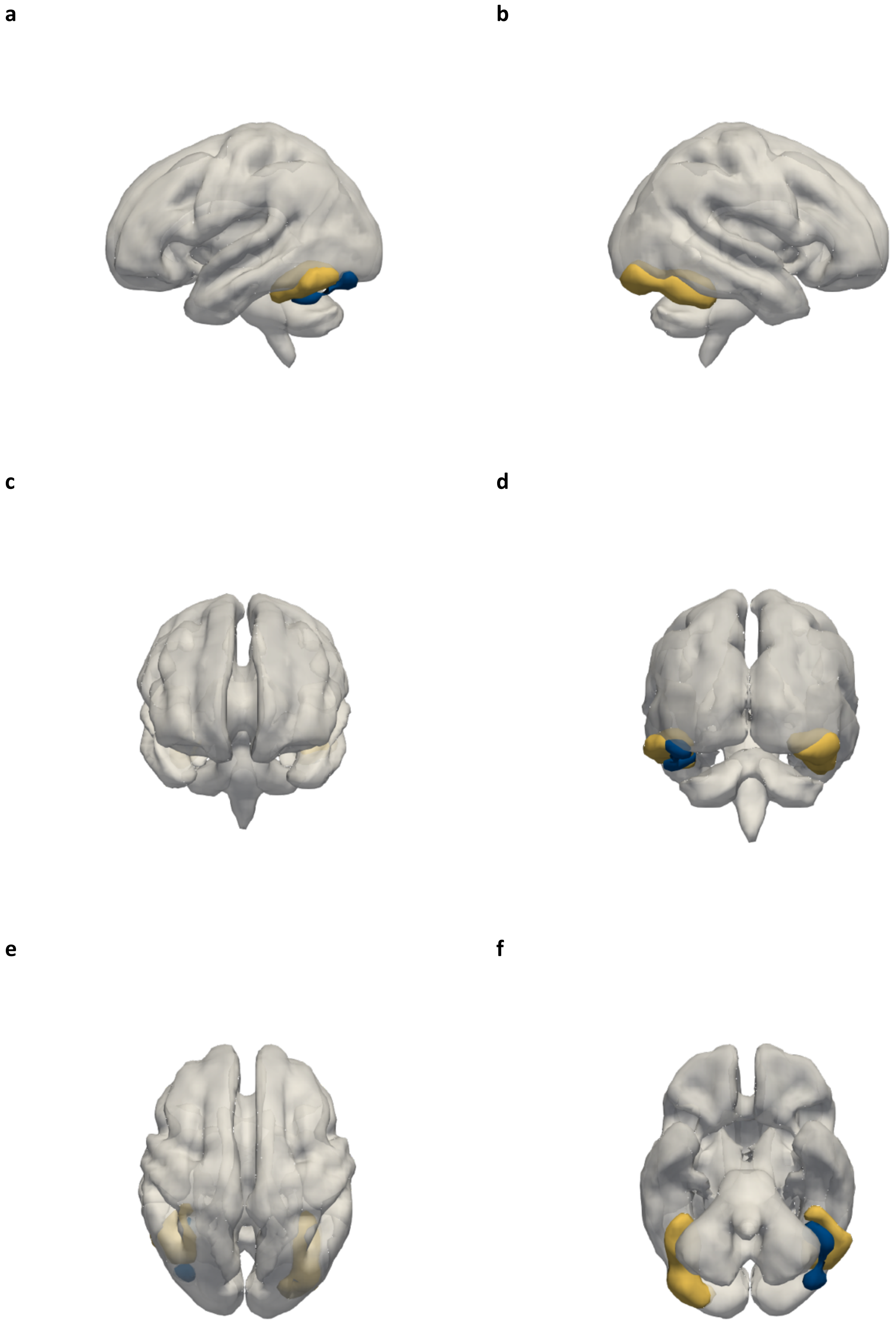

Three-dimensional rendering of the functional grey matter network representing *visual recognition*. The colours label functional subnetworks separated by microarray gene expression data, here represented in yellow and blue. This forms the basis upon which treatment effect heterogeneity is simulated, with hypothetical treatments selectively effective for lesions disrupting defined subnetworks. Each panel shows the same render from a different spatial perspective: **a**, left; **b**, right; **c**, anterior; **d**, posterior, **e**, superior; **f**, inferior.

Supplementary Figure 66: *Visual Recognition* subnetwork by transcriptome slices

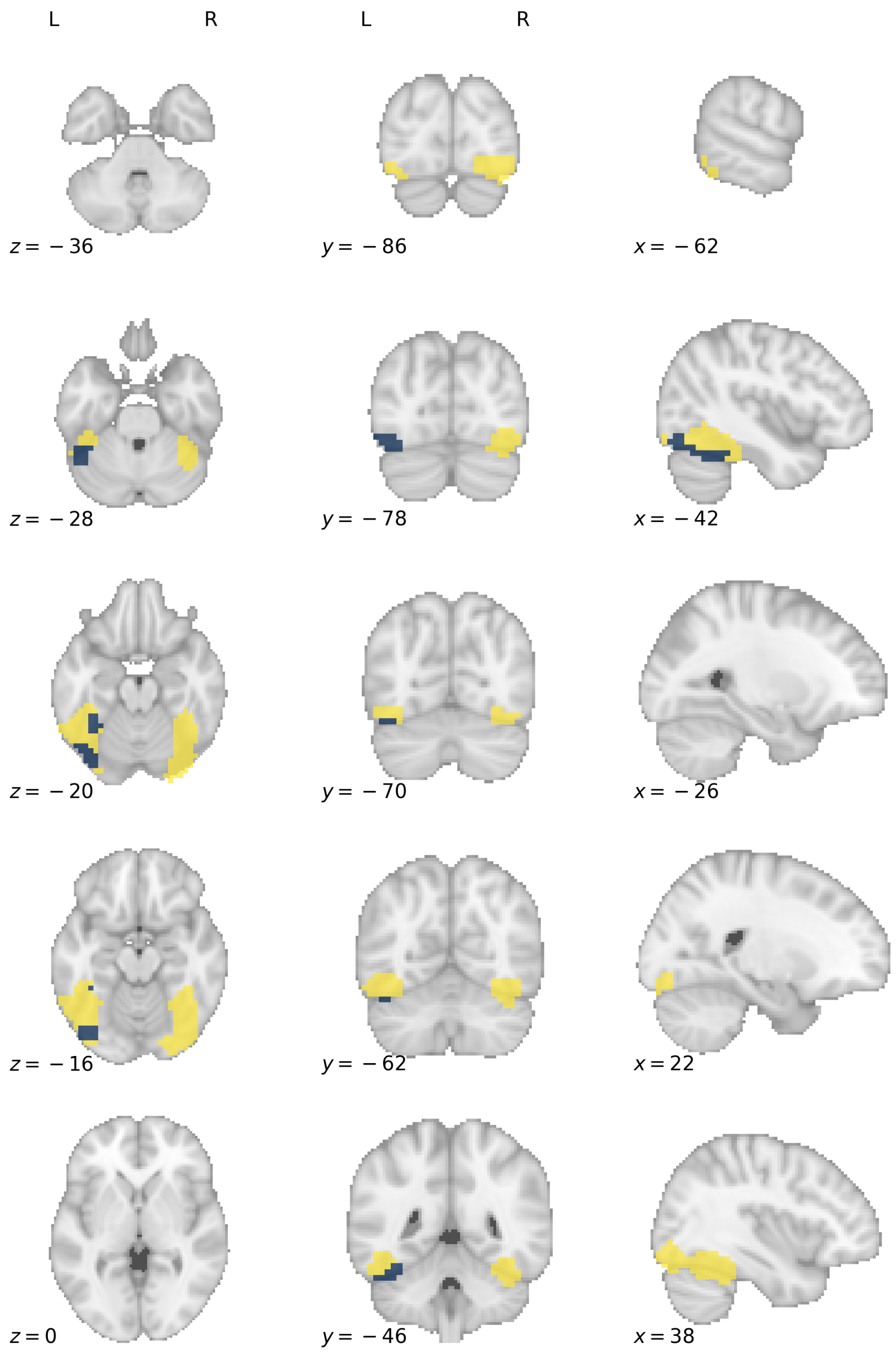

Slice visualization of the functional grey matter network representing *visual recognition*, overlaid onto the standard MNI152 template. The labelled co-ordinates map to MNI space. The colours label functional subnetworks separated by microarray gene expression, here represented in yellow and blue. This forms the basis upon which treatment effect heterogeneity is simulated, with hypothetical treatments selectively effective for lesions disrupting defined subnetworks.

Supplementary Figure 67: *Visual Perception* subnetwork by transcriptome render

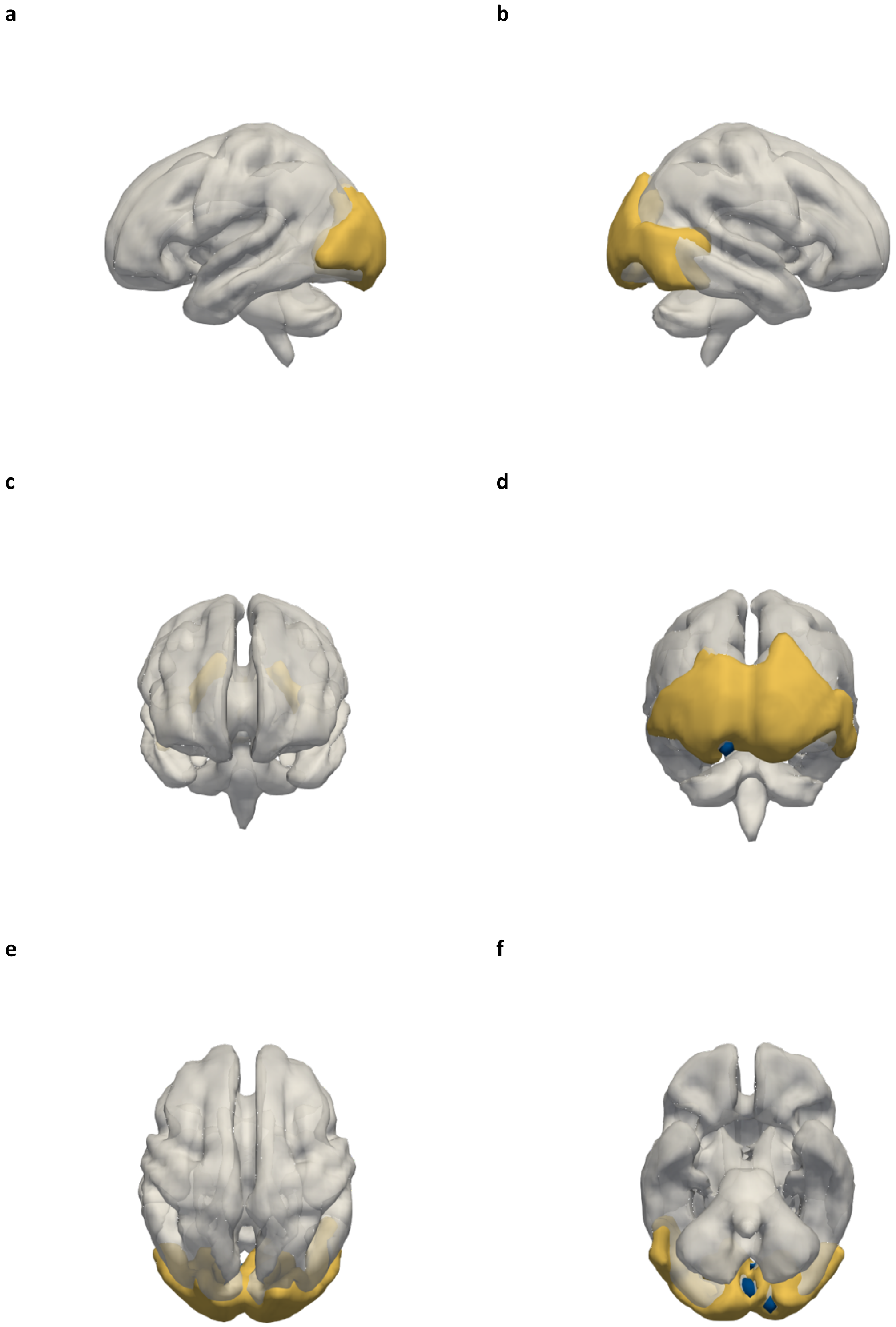

Three-dimensional rendering of the functional grey matter network representing *visual perception*. The colours label functional subnetworks separated by microarray gene expression data, here represented in yellow and blue. This forms the basis upon which treatment effect heterogeneity is simulated, with hypothetical treatments selectively effective for lesions disrupting defined subnetworks. Each panel shows the same render from a different spatial perspective: **a**, left; **b**, right; **c**, anterior; **d**, posterior, **e**, superior; **f**, inferior.

Supplementary Figure 68: *Visual Perception* subnetwork by transcriptome slices

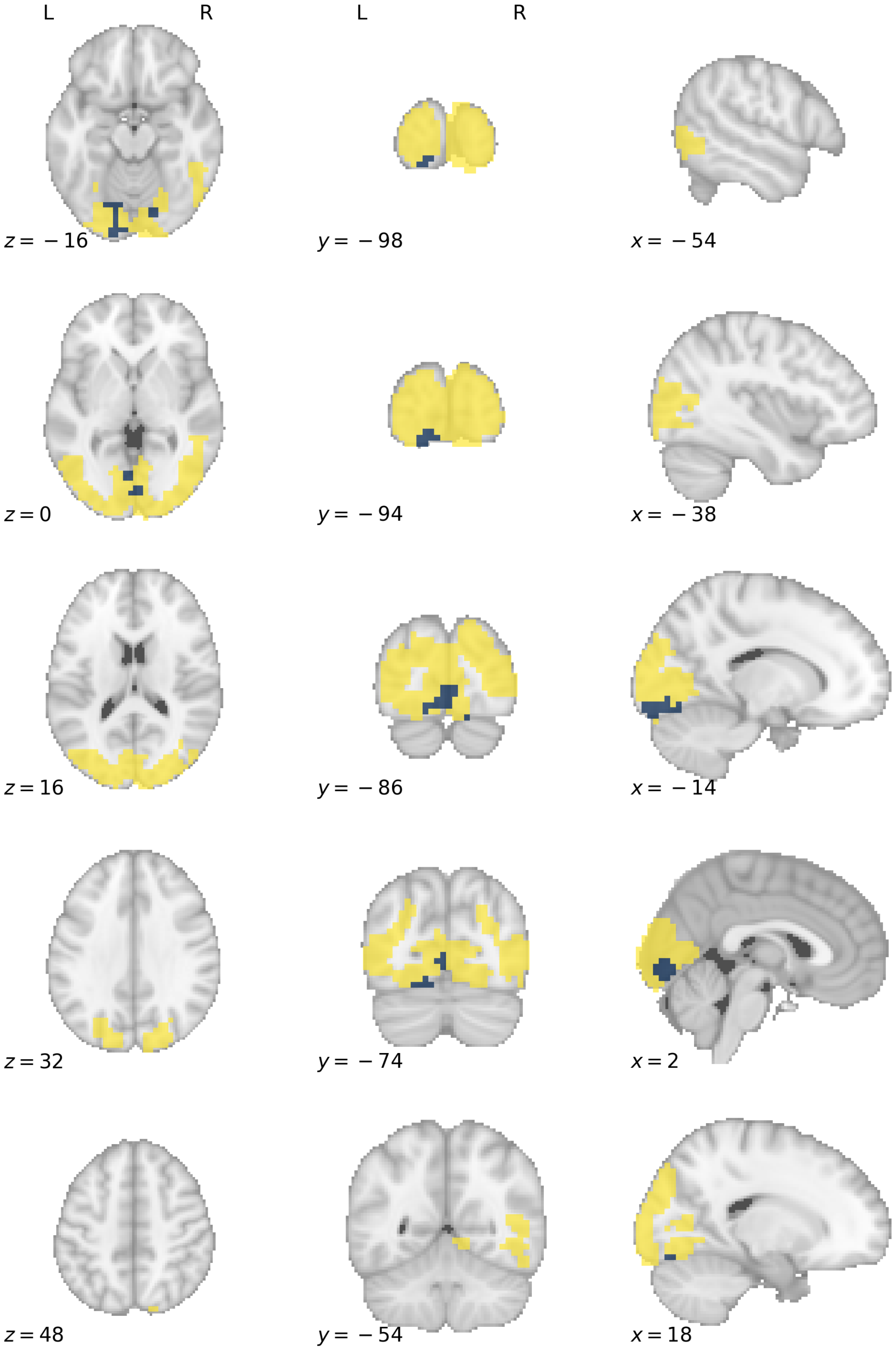

Slice visualization of the functional grey matter network representing *visual perception*, overlaid onto the standard MNI152 template. The labelled co-ordinates map to MNI space. The colours label functional subnetworks separated by microarray gene expression, here represented in yellow and blue. This forms the basis upon which treatment effect heterogeneity is simulated, with hypothetical treatments selectively effective for lesions disrupting defined subnetworks.

Supplementary Figure 69: *Spatial Reasoning* subnetwork by transcriptome render

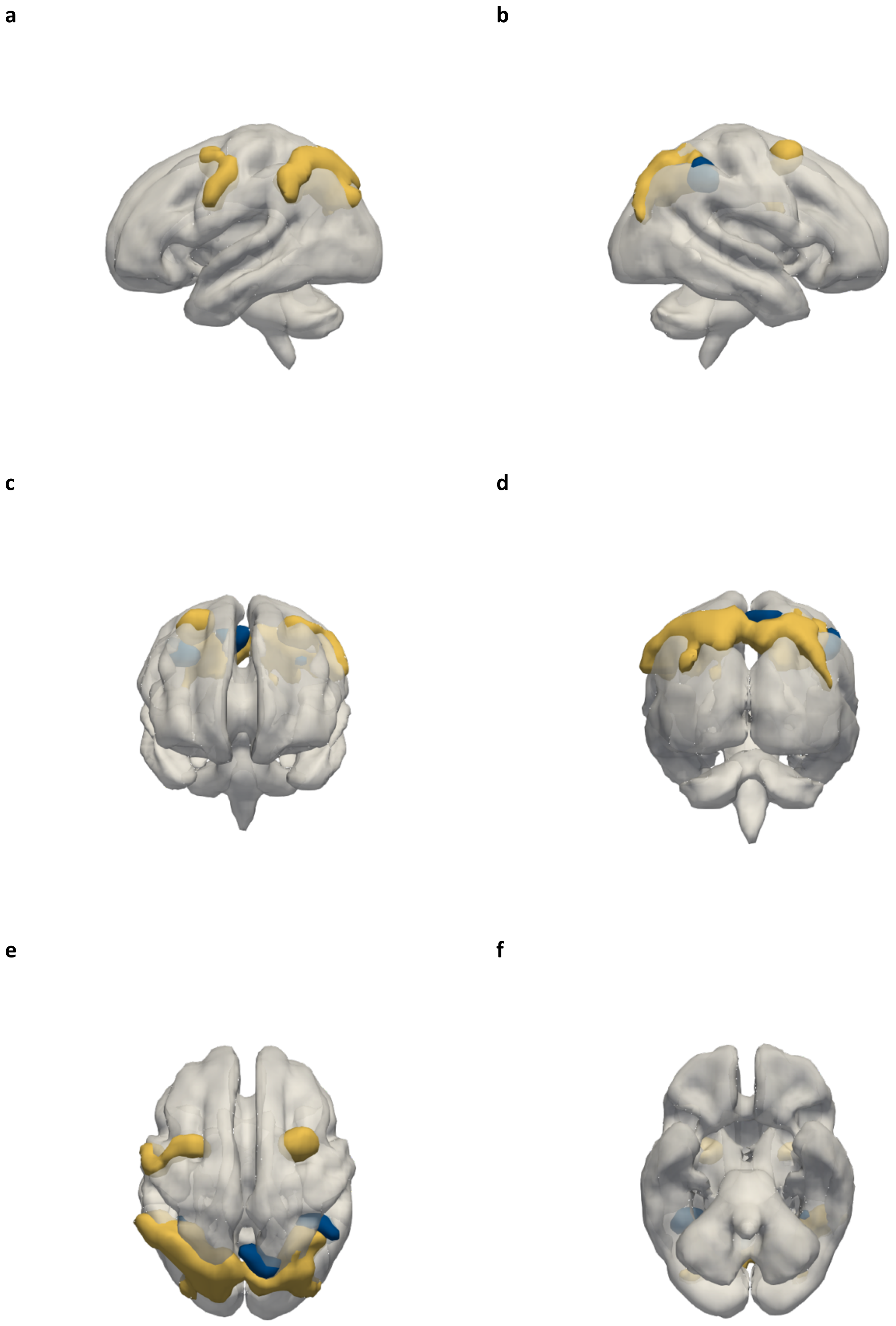

Three-dimensional rendering of the functional grey matter network representing *spatial reasoning*. The colours label functional subnetworks separated by microarray gene expression data, here represented in yellow and blue. This forms the basis upon which treatment effect heterogeneity is simulated, with hypothetical treatments selectively effective for lesions disrupting defined subnetworks. Each panel shows the same render from a different spatial perspective: **a**, left; **b**, right; **c**, anterior; **d**, posterior, **e**, superior; **f**, inferior.

Supplementary Figure 70: *Spatial Reasoning* subnetwork by transcriptome slices

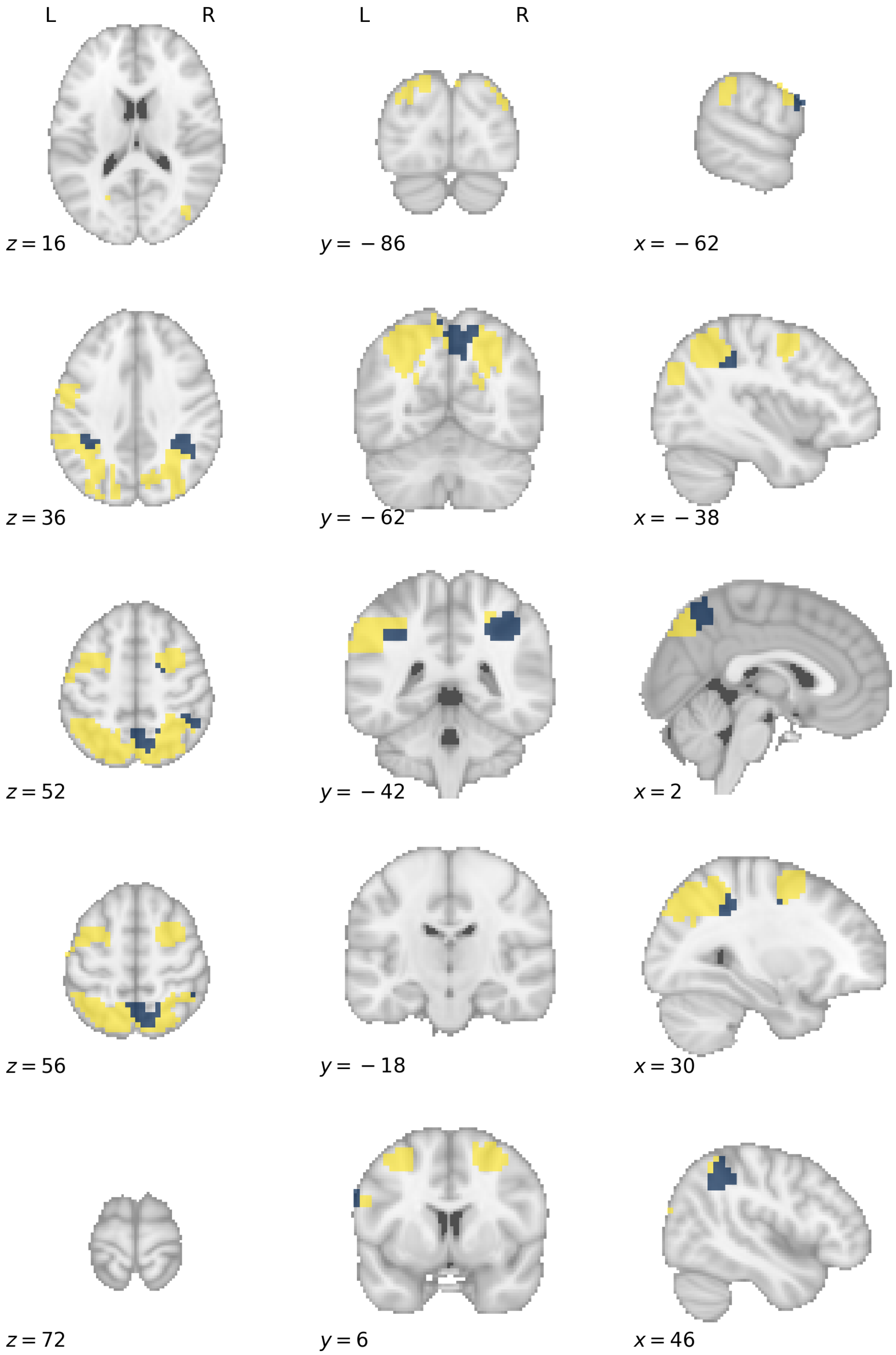

Slice visualization of the functional grey matter network representing *spatial reasoning*, overlaid onto the standard MNI152 template. The labelled co-ordinates map to MNI space. The colours label functional subnetworks separated by microarray gene expression, here represented in yellow and blue. This forms the basis upon which treatment effect heterogeneity is simulated, with hypothetical treatments selectively effective for lesions disrupting defined subnetworks.

Supplementary Figure 71: *Motor* subnetwork by transcriptome render

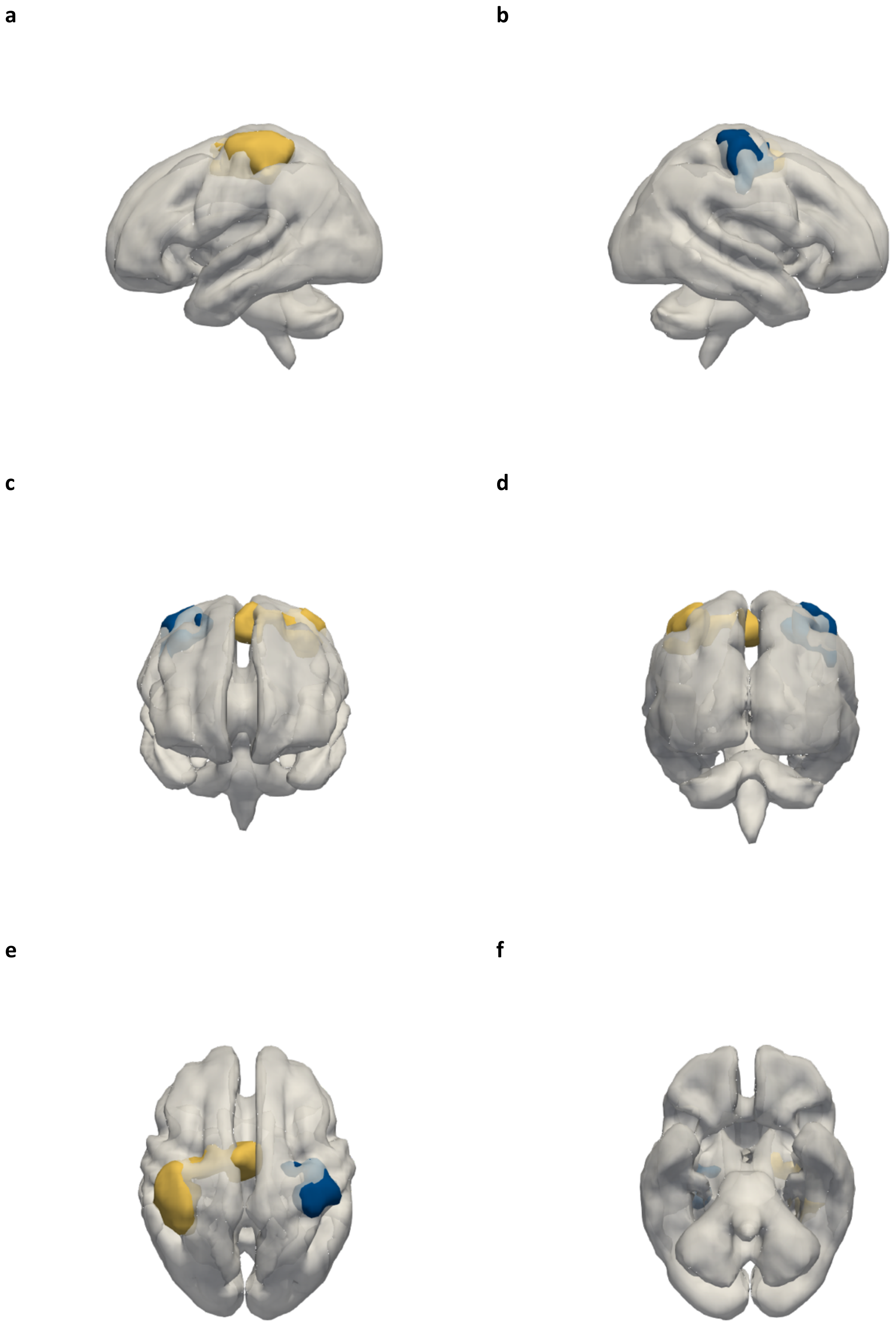

Three-dimensional rendering of the functional grey matter network representing *motor behaviour*. The colours label functional subnetworks separated by microarray gene expression data, here represented in yellow and blue. This forms the basis upon which treatment effect heterogeneity is simulated, with hypothetical treatments selectively effective for lesions disrupting defined subnetworks. Each panel shows the same render from a different spatial perspective: **a**, left; **b**, right; **c**, anterior; **d**, posterior, **e**, superior; **f**, inferior.

Supplementary Figure 72: *Motor* subnetwork by transcriptome slices

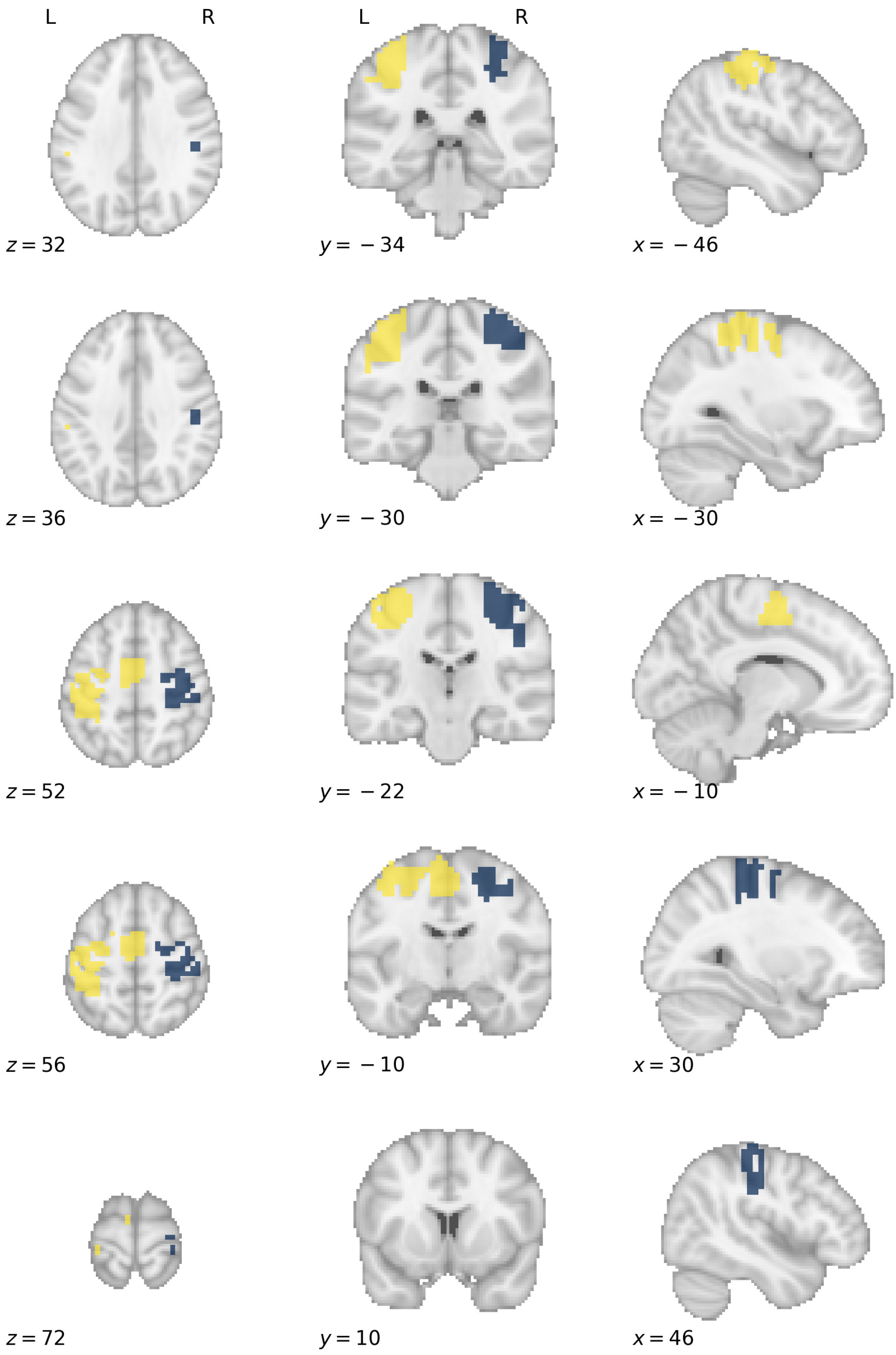

Slice visualization of the functional grey matter network representing *motor behaviour*, overlaid onto the standard MNI152 template. The labelled co-ordinates map to MNI space. The colours label functional subnetworks separated by microarray gene expression, here represented in yellow and blue. This forms the basis upon which treatment effect heterogeneity is simulated, with hypothetical treatments selectively effective for lesions disrupting defined subnetworks.

Supplementary Figure 73: *Somatosensory* subnetwork by transcriptome render

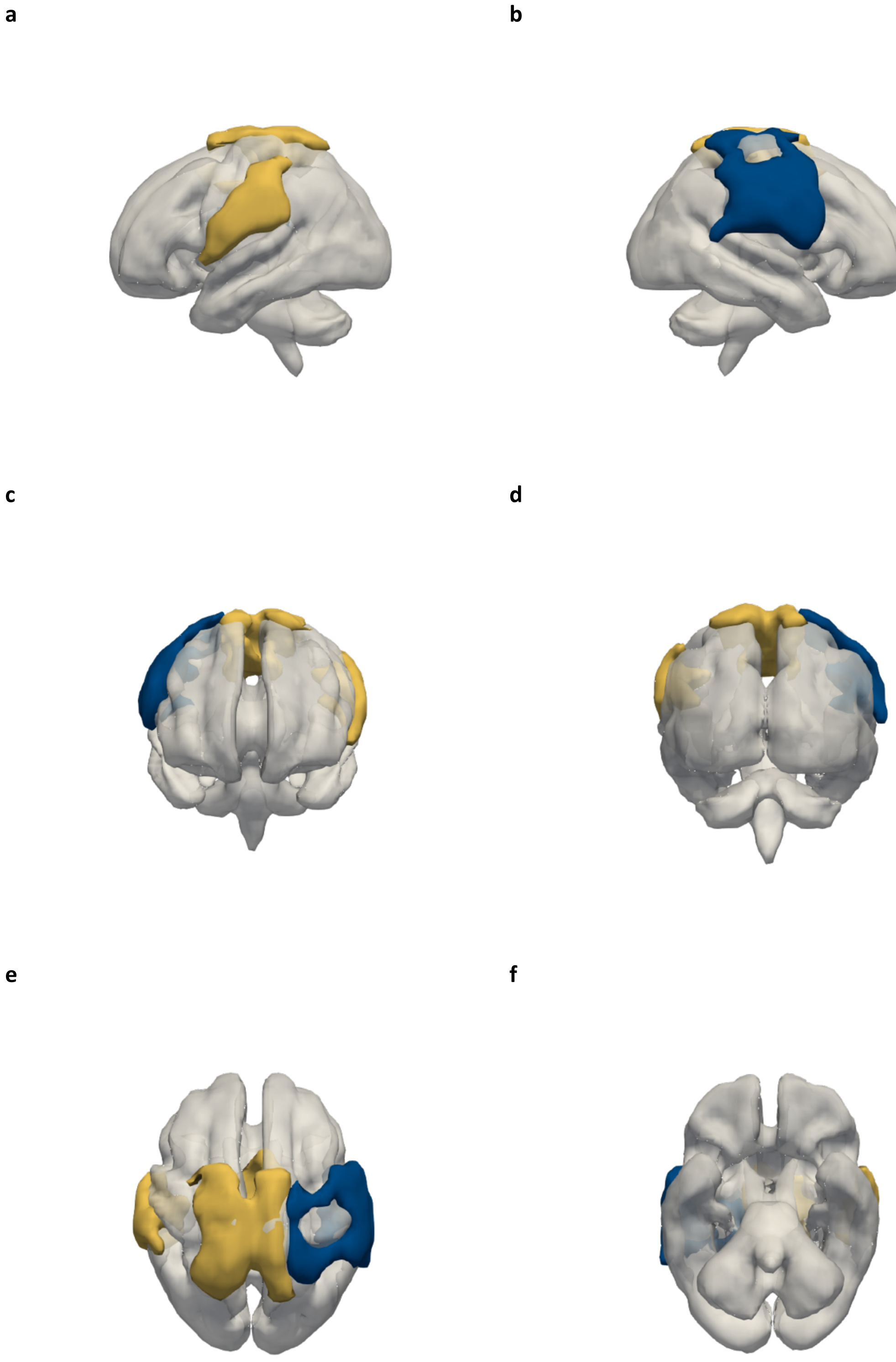

Three-dimensional rendering of the functional grey matter network representing *somatosensory function*. The colours label functional subnetworks separated by microarray gene expression data, here represented in yellow and blue. This forms the basis upon which treatment effect heterogeneity is simulated, with hypothetical treatments selectively effective for lesions disrupting defined subnetworks. Each panel shows the same render from a different spatial perspective: **a**, left; **b**, right; **c**, anterior; **d**, posterior, **e**, superior; **f**, inferior.

Supplementary Figure 74: *Somatosensory* subnetwork by transcriptome slices

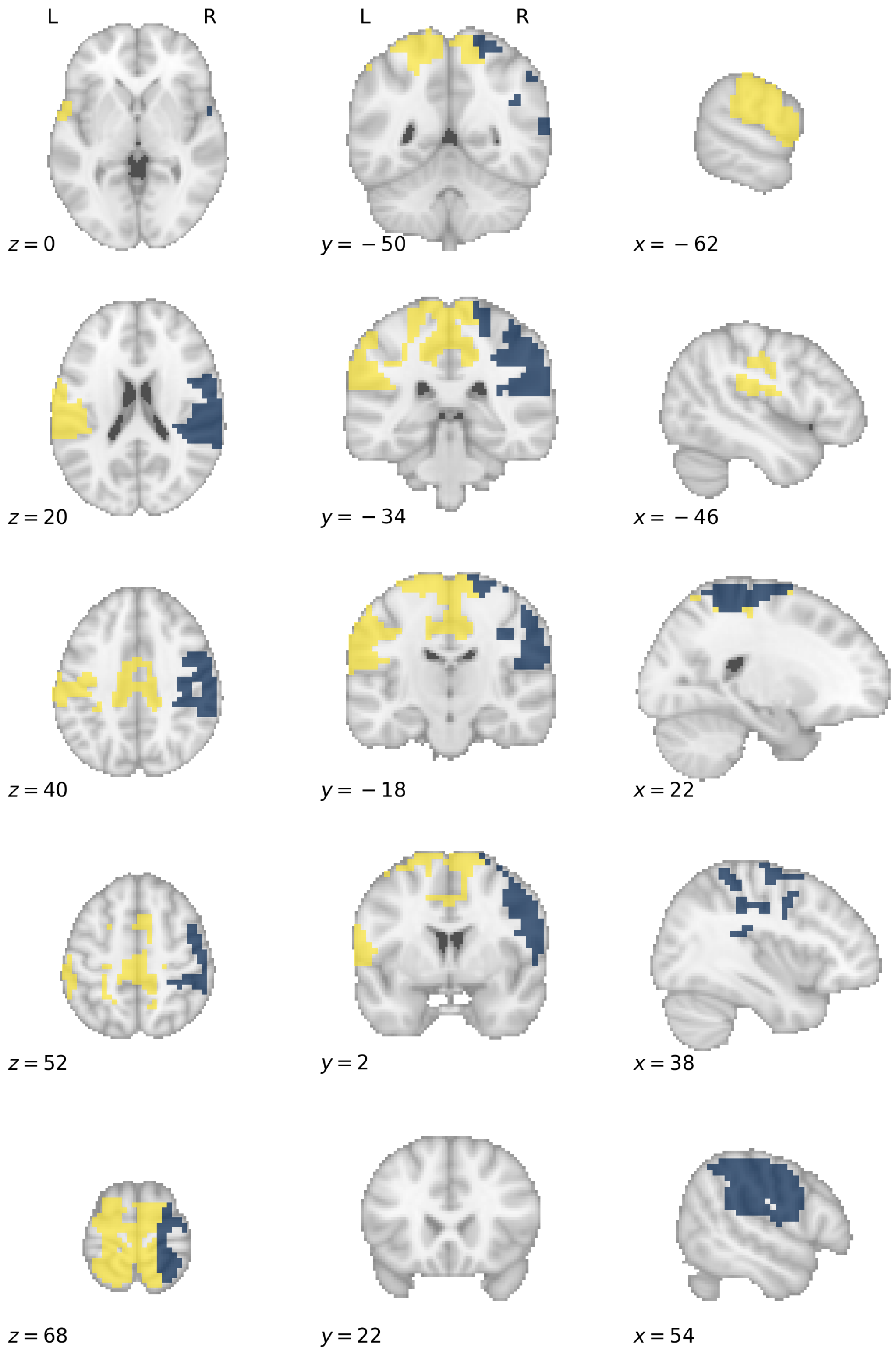

Slice visualization of the functional grey matter network representing *somatosensory function*, overlaid onto the standard MNI152 template. The labelled co-ordinates map to MNI space. The colours label functional subnetworks separated by microarray gene expression, here represented in yellow and blue. This forms the basis upon which treatment effect heterogeneity is simulated, with hypothetical treatments selectively effective for lesions disrupting defined subnetworks.

Supplementary Table 4: Full results table for lesion representations & location bias conditions

Representation	Model	Configuration	Balanced accuracy	PEHE
VAE	ExtraTrees	Two	***0.638095***	***0.670712***
VAE	Logistic regression	Two	0.632575	0.706211
VAE	Random forest	Two	0.632377	0.673765
AE	Logistic regression	Two	0.630802	0.711604
AE	ExtraTrees	Two	0.630651	0.676929
PCA	ExtraTrees	Two	0.627552	0.680252
PCA	Random forest	Two	0.625893	0.676612
AE	Random forest	Two	0.624254	0.678743
VAE	ExtraTrees	One	0.620283	0.695618
VAE	XGBoost	Two	0.620071	0.712113
PCA	XGBoost	Two	0.618296	0.714805
AE	XGBoost	Two	0.615551	0.715816
AE	ExtraTrees	One	0.614499	0.697340
PCA	ExtraTrees	One	0.613021	0.694528
PCA	Logistic regression	Two	0.612483	0.731578
NMF	ExtraTrees	Two	0.604669	0.705303
NMF	ExtraTrees	One	0.603761	0.705476
NMF	Random forest	Two	0.600153	0.700542
NMF	Random forest	One	0.580878	0.725727
VAE	Random forest	One	0.578627	0.730461
AE	Random forest	One	0.576798	0.729762
PCA	Random forest	One	0.575839	0.730990
NMF	XGBoost	Two	0.570808	0.756893
AE	XGBoost	One	0.560585	0.715964
VAE	XGBoost	One	0.559008	0.716564
PCA	XGBoost	One	0.555964	0.715761
VAE	Gaussian process	One	0.553557	0.759963
NMF	XGBoost	One	0.553063	0.720112
VAE	Gaussian process	Two	0.550553	0.758173
NMF	Gaussian process	Two	0.532766	0.712642
NMF	Logistic regression	Two	0.531445	0.709866
NMF	Gaussian process	One	0.528550	0.713111
AE	Gaussian process	One	0.527859	0.760956
PCA	Gaussian process	One	0.527066	0.757244
PCA	Gaussian process	Two	0.526727	0.755334
AE	Gaussian process	Two	0.525933	0.759227
PCA	Logistic regression	One	0.484691	0.726026
AE	Logistic regression	One	0.484600	0.726624
VAE	Logistic regression	One	0.484444	0.725735
NMF	Logistic regression	One	0.484085	0.724560

Supplementary Figure 75: Optimal model for lesion representations & location bias conditions by balanced accuracy: VAE, two-model ExtraTrees

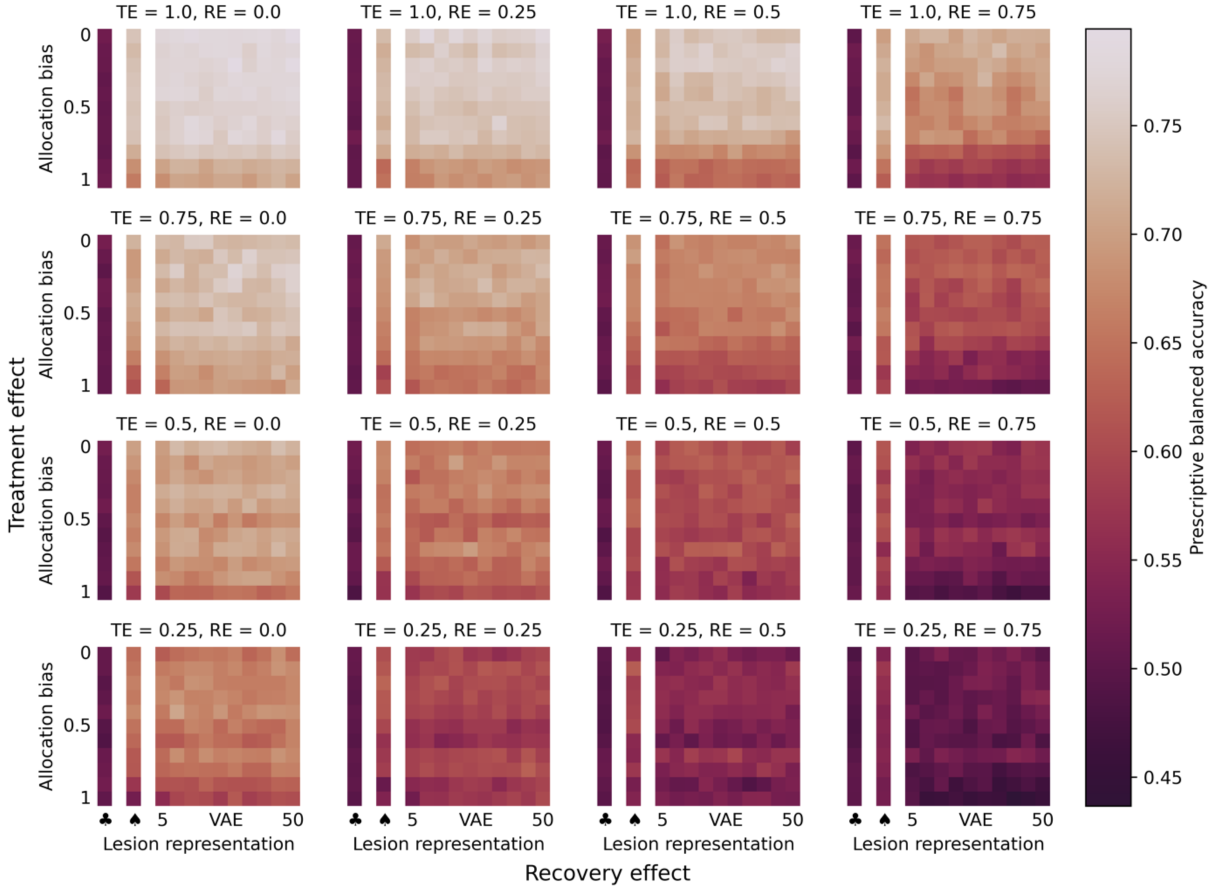

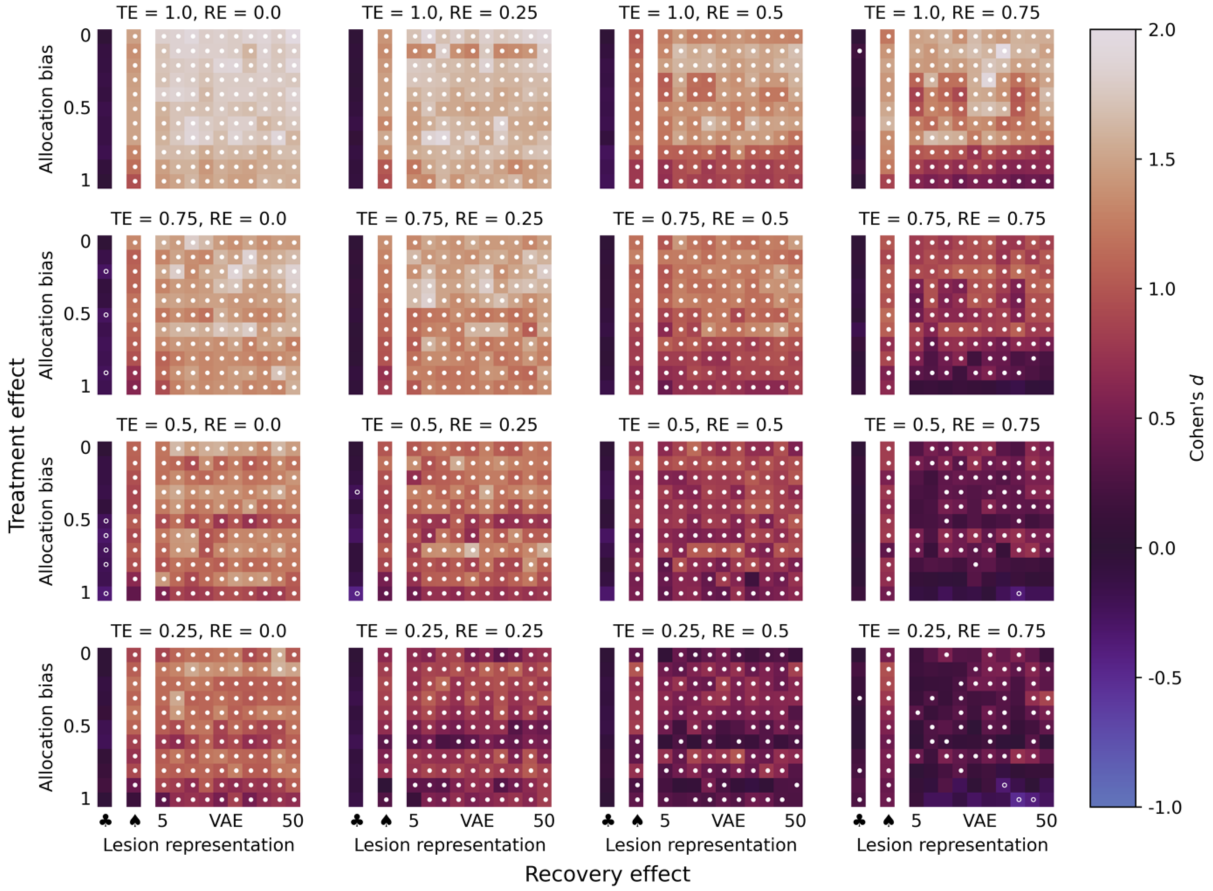

Supplementary Figure 75. Performance comparison of models with lesion representations: balanced accuracy (upper panel) and statistical analysis (lower panel). Prescriptive performance of an optimized, extremely randomized trees-based treatment recommendation system, given binary lesion representations, across the range of response noise (major axes) and allocation bias (minor y-axes), at various levels of expressivity of the individualized representation quantified by embedding length (minor x-axes). The columns to the left of each minor axis show prescriptive performance when individual phenotypes are represented by classification of major affected arterial territory: anterior or posterior circulation (♣) and ACA/MCA/posterior cerebral artery/VB (♠). The upper left subplot shows performance under zero response noise and the lower right subplot shows performance under the conditions of extremely high response noise. The top row of each subplot shows perfect randomization, with zero allocation bias. The upper panel shows prescriptive performance measured by balanced accuracy—the mean proportion of patients allocated to the treatment for which they are truly responsive—for patients that are exclusively responsive to one treatment.

The lower panel shows Cohen's d-effect sizes, by colour, when comparing the prescriptive performance (measured by balanced accuracy, as shown in the upper panel) against a randomized trial based upon individualized information consisting of the major vascular supply (anterior or posterior circulation), at each respective TE/RE pair. Warm colours show performance greater than the comparative randomized trial, while cool colours show the converse. White shows equivalent performance. Trials marked with a circle indicate an effect size exceeding the critical p-value 0.0423 (as adjusted for multiple comparisons at the 0.05 significance level, according to the Benjamini–Hochberg procedure), corresponding to the t-value in a two-sided independent sample t-test. Filled circles show superiority of the prescriptive model from observational data; unfilled circles show superiority of the randomized trial.

TE: treatment effect; RE: recovery effect; VAE: variational auto-encoder representation; ACA: anterior cerebral artery; MCA: middle cerebral artery; VB: vertebrobasilar artery

Supplementary Figure 76: Optimal model for lesion representations & location bias conditions by PEHE: VAE, two-model ExtraTrees

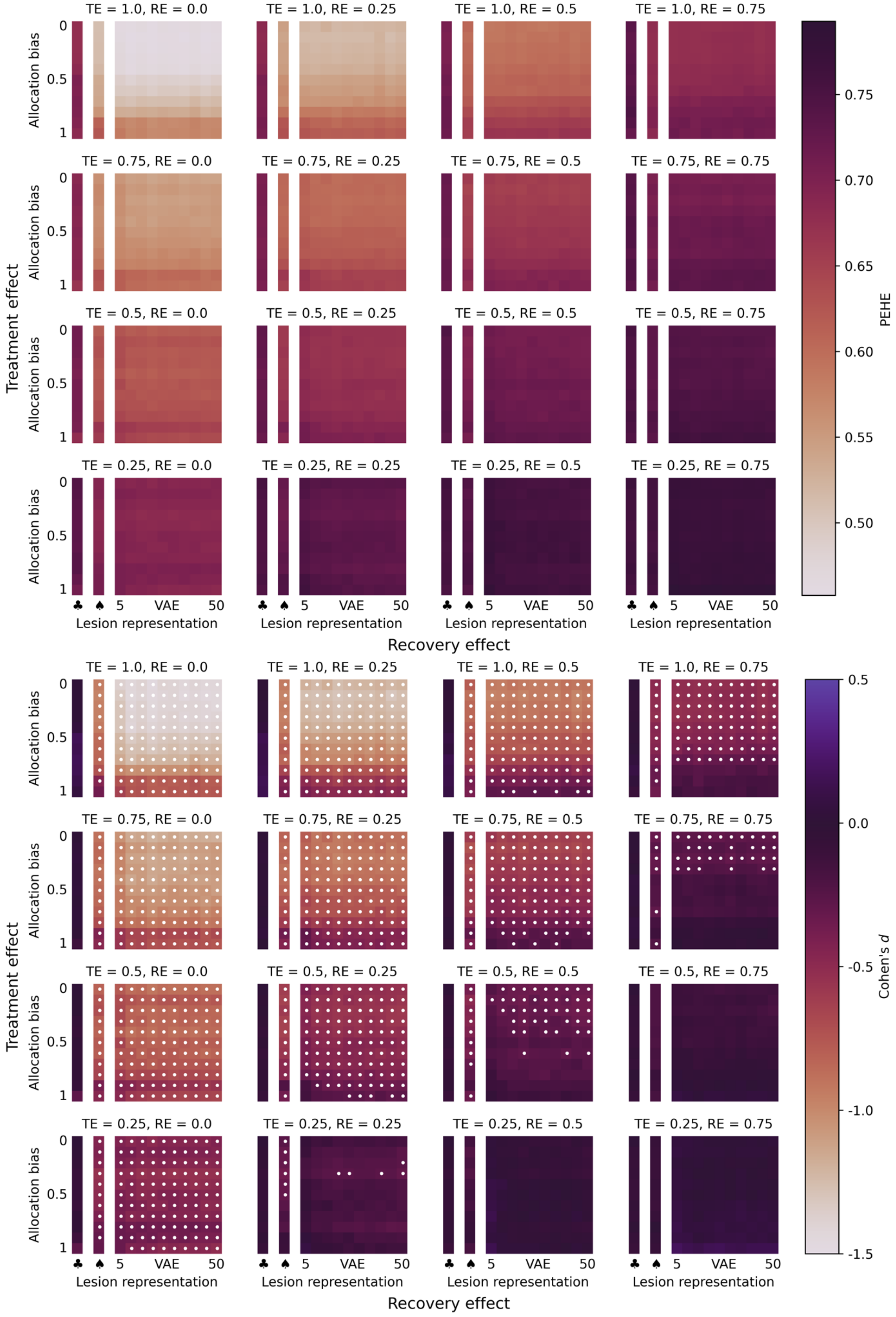

Supplementary Figure 76. Performance comparison of models with lesion representations: PEHE (upper panel) and statistical analysis (lower panel). Prescriptive performance of an optimized, extremely randomized trees-based treatment recommendation system, given binary lesion representations, across the range of response noise (major axes) and allocation bias (minor y-axes), at various levels of expressivity of the individualized representation quantified by embedding length (minor x-axes). The columns to the left of each minor axis show prescriptive performance when individual phenotypes are represented by classification of major affected arterial territory: anterior or posterior circulation (♣) and ACA/MCA/posterior cerebral artery/VB (♠). The upper left subplot shows performance under zero response noise and the lower right subplot shows performance under the conditions of extremely high response noise. The top row of each subplot shows perfect randomization, with zero allocation bias. The upper panel shows prescriptive performance measured by PEHE the root-mean-squared-error in estimation of the true individualized treatment effect (lower is better).

The lower panel shows Cohen's d-effect sizes, by colour, when comparing the prescriptive performance (measured by PEHE, as shown in the upper panel) against a randomized trial based upon individualized information consisting of the major vascular supply (anterior or posterior circulation), at each respective TE/RE pair. Warm colours show performance greater than the comparative randomized trial, while cool colours show the converse. White shows equivalent performance. Trials marked with a circle indicate an effect size exceeding the critical p-value 0.0285 (as adjusted for multiple comparisons at the 0.05 significance level, according to the Benjamini–Hochberg procedure), corresponding to the t-value in a two-sided independent sample t-test. Filled circles show superiority of the prescriptive model from observational data; unfilled circles show superiority of the randomized trial.

TE: treatment effect; RE: recovery effect; VAE: variational auto-encoder representation; ACA: anterior cerebral artery; MCA: middle cerebral artery; VB: vertebrobasilar artery

Supplementary Table 5: Full results table for disconnectome representations & location bias conditions

Representation	Model	Configuration	Balanced accuracy	PEHE
AE	ExtraTrees	Two	***0.718161***	0.573161
VAE	ExtraTrees	Two	0.712637	0.576710
AE	Logistic regression	Two	0.711435	***0.564923***
AE	Random forest	Two	0.708890	0.574772
PCA	ExtraTrees	Two	0.707576	0.579157
VAE	Logistic regression	Two	0.705775	0.568535
AE	ExtraTrees	One	0.705127	0.593895
VAE	Random forest	Two	0.704094	0.578082
VAE	ExtraTrees	One	0.699950	0.596088
AE	XGBoost	Two	0.699258	0.611886
VAE	XGBoost	Two	0.695058	0.616278
PCA	Random forest	Two	0.694213	0.581038
PCA	ExtraTrees	One	0.694118	0.596600
PCA	Logistic regression	Two	0.691628	0.590505
PCA	XGBoost	Two	0.687607	0.617454
NMF	Gaussian process	Two	0.686881	0.617289
NMF	Gaussian process	One	0.682916	0.617831
NMF	ExtraTrees	Two	0.680377	0.597582
NMF	Logistic regression	Two	0.669639	0.583176
NMF	ExtraTrees	One	0.666861	0.614481
AE	Random forest	One	0.661850	0.629989
VAE	Random forest	One	0.658928	0.631005
AE	XGBoost	One	0.653617	0.608419
VAE	XGBoost	One	0.650446	0.610225
NMF	Random forest	Two	0.648854	0.620553
NMF	XGBoost	Two	0.648703	0.646442
PCA	Random forest	One	0.647613	0.631801
PCA	XGBoost	One	0.638673	0.611830
NMF	XGBoost	One	0.629108	0.617982
NMF	Random forest	One	0.612928	0.646711
AE	Gaussian process	One	0.612842	0.679290
AE	Gaussian process	Two	0.610761	0.679116
VAE	Gaussian process	One	0.609586	0.676570
VAE	Gaussian process	Two	0.607783	0.676405
PCA	Gaussian process	Two	0.515168	0.684232
PCA	Gaussian process	One	0.514813	0.684112
PCA	Logistic regression	One	0.500099	0.633768
VAE	Logistic regression	One	0.500000	0.634573
AE	Logistic regression	One	0.500000	0.634102
NMF	Logistic regression	One	0.500000	0.634233

Supplementary Figure 77: Optimal model for disconnectome representations & location bias conditions by balanced accuracy: AE, two-model ExtraTrees

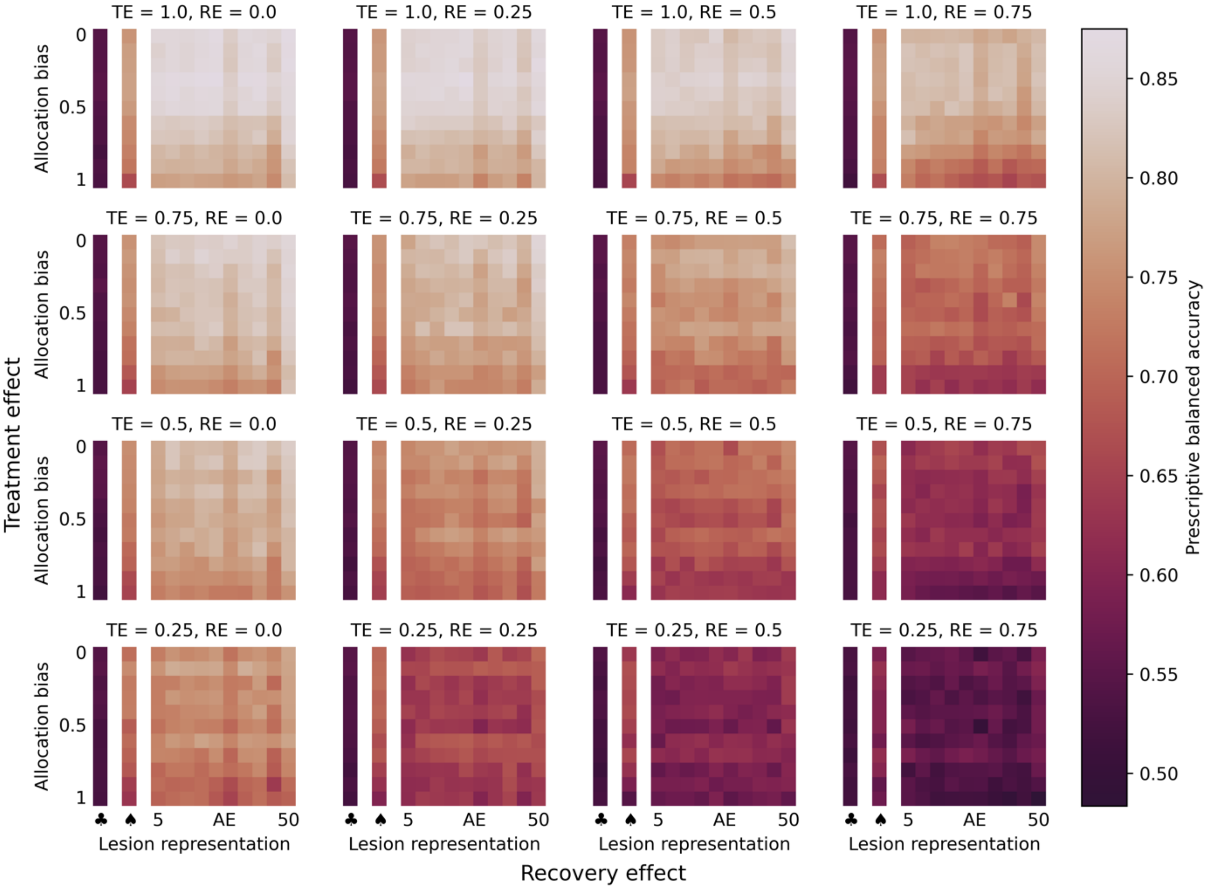

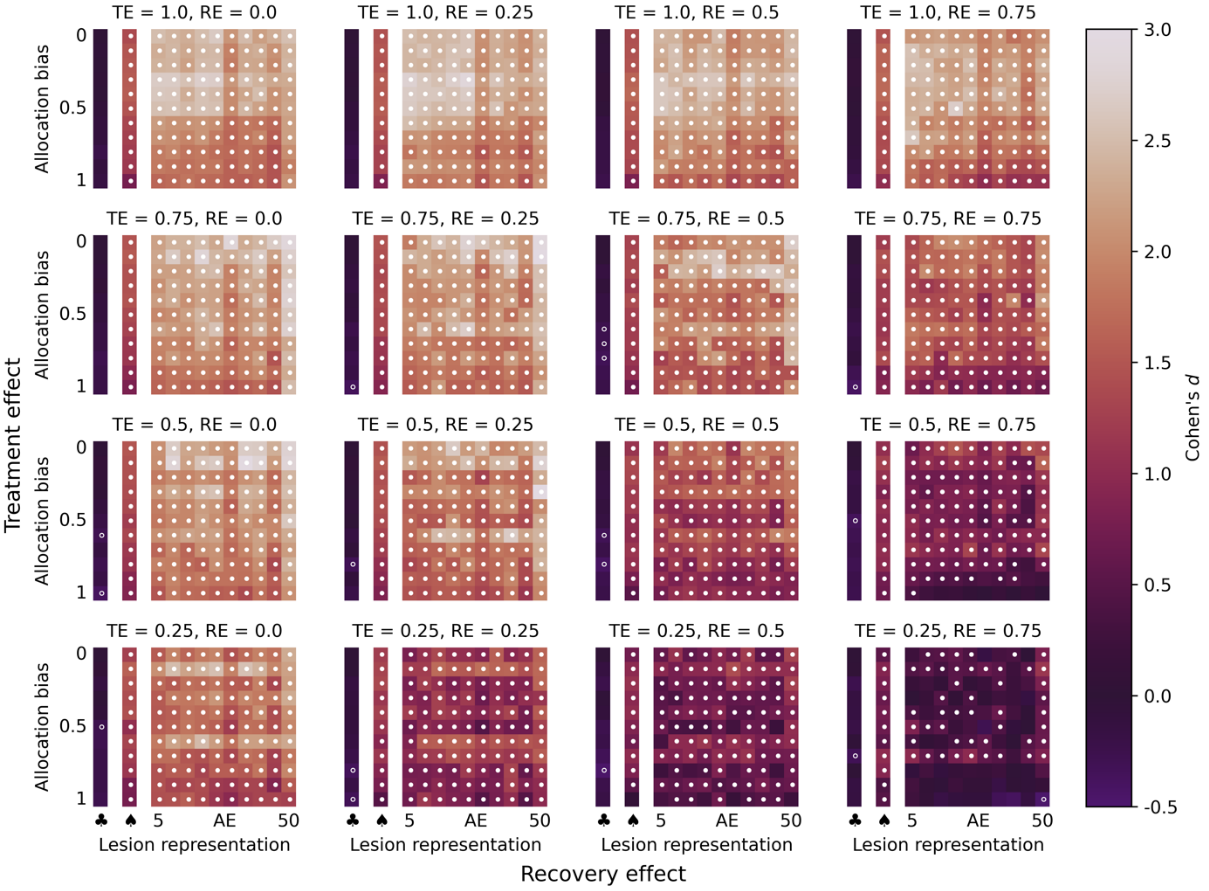

Supplementary Figure 77. Performance comparison of models with disconnectome representations: balanced accuracy (upper panel) and statistical analysis (lower panel). Prescriptive performance of an optimized, extremely randomized trees-based treatment recommendation system, given lesion disconnectome representations, across the range of response noise (major axes) and allocation bias (minor y-axes), at various levels of expressivity of the individualized representation quantified by embedding length (minor x-axes). The columns to the left of each minor axis show prescriptive performance when individual phenotypes are represented by classification of major affected arterial territory: anterior or posterior circulation (♣) and ACA/MCA/posterior cerebral artery/VB (♠). The upper left subplot shows performance under zero response noise and the lower right subplot shows performance under the conditions of extremely high response noise. The top row of each subplot shows perfect randomization, with zero allocation bias. The upper panel shows prescriptive performance measured by balanced accuracy—the mean proportion of patients allocated to the treatment for which they are truly responsive—for patients that are exclusively responsive to one treatment.

The lower panel shows Cohen's d-effect sizes, by colour, when comparing the prescriptive performance (measured by balanced accuracy, as shown in the upper panel) against a randomized trial based upon individualized information consisting of the major vascular supply (anterior or posterior circulation), at each respective TE/RE pair. Warm colours show performance greater than the comparative randomized trial, while cool colours show the converse. White shows equivalent performance. Trials marked with a circle indicate an effect size exceeding the critical p-value 0.0439 (as adjusted for multiple comparisons at the 0.05 significance level, according to the Benjamini–Hochberg procedure), corresponding to the t-value in a two-sided independent sample t-test. Filled circles show superiority of the prescriptive model from observational data; unfilled circles show superiority of the randomized trial.

TE: treatment effect; RE: recovery effect; AE: auto-encoder representation; ACA: anterior cerebral artery; MCA: middle cerebral artery; VB: vertebrobasilar artery

Supplementary Figure 78: Optimal model for disconnectome representations & location bias conditions by PEHE: AE, two-model logistic regression

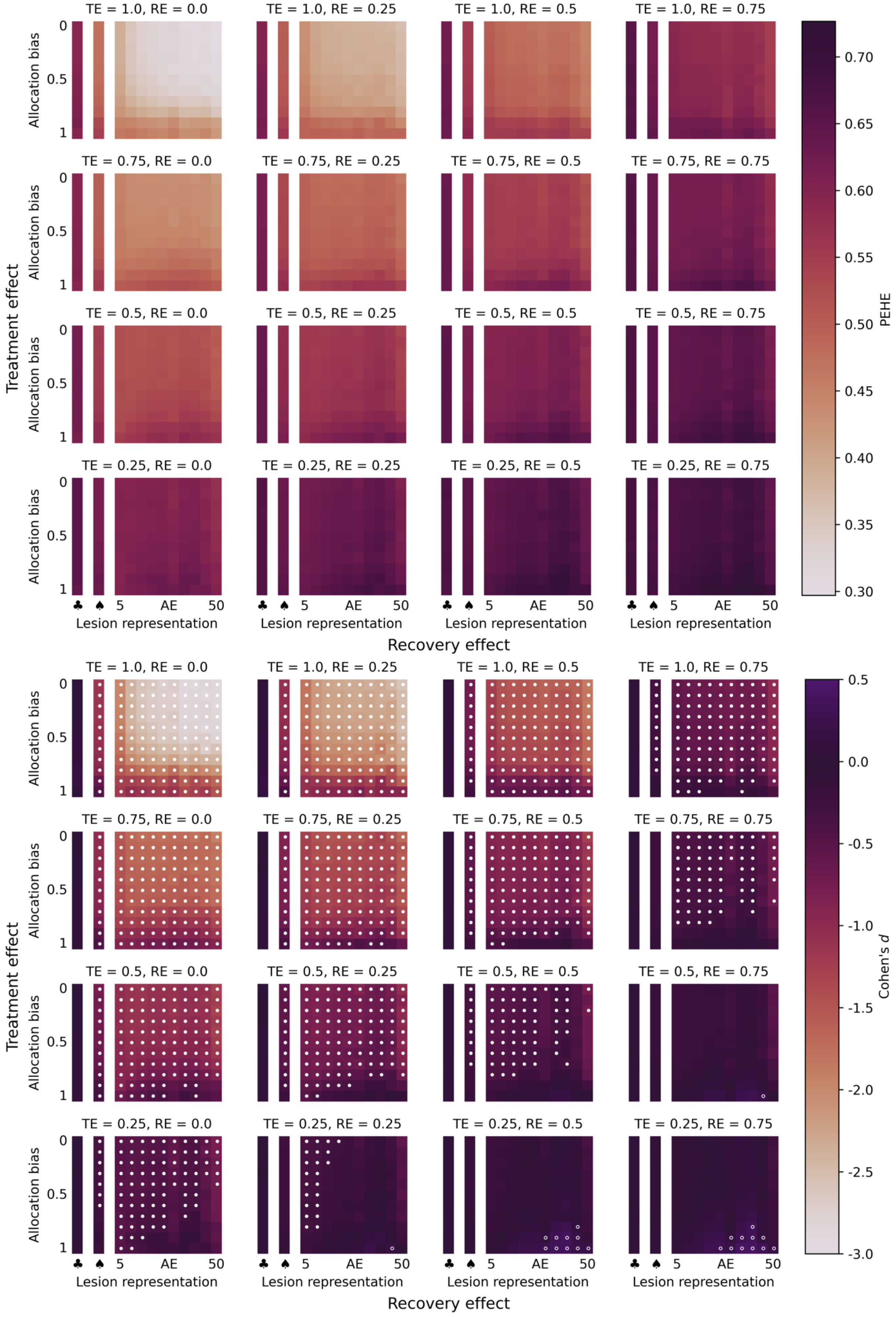

Supplementary Figure 78. Performance comparison of models with disconnectome representations: PEHE (upper panel) and statistical analysis (lower panel). Prescriptive performance of an optimized, logistic regression-based treatment recommendation system, given lesion disconnectome representations, across the range of response noise (major axes) and allocation bias (minor y-axes), at various levels of expressivity of the individualized representation quantified by embedding length (minor x-axes). The columns to the left of each minor axis show prescriptive performance when individual phenotypes are represented by classification of major affected arterial territory: anterior or posterior circulation (♣) and ACA/MCA/posterior cerebral artery/VB (♠). The upper left subplot shows performance under zero response noise and the lower right subplot shows performance under the conditions of extremely high response noise. The top row of each subplot shows perfect randomization, with zero allocation bias. The upper panel shows prescriptive performance measured by PEHE the root-mean-squared-error in estimation of the true individualized treatment effect (lower is better).

The lower panel shows Cohen's d-effect sizes, by colour, when comparing the prescriptive performance (measured by PEHE, as shown in the upper panel) against a randomized trial based upon individualized information consisting of the major vascular supply (anterior or posterior circulation), at each respective TE/RE pair. Warm colours show performance greater than the comparative randomized trial, while cool colours show the converse. White shows equivalent performance. Trials marked with a circle indicate an effect size exceeding the critical p-value 0.0308 (as adjusted for multiple comparisons at the 0.05 significance level, according to the Benjamini-Hochberg procedure), corresponding to the t-value in a two-sided independent sample t-test. Filled circles show superiority of the prescriptive model from observational data; unfilled circles show superiority of the randomized trial.

TE: treatment effect; RE: recovery effect; AE: auto-encoder representation; ACA: anterior cerebral artery; MCA: middle cerebral artery; VB: vertebrobasilar artery

Supplementary Table 6: Full results table for lesion representations & unobservable bias conditions

Representation	Model	Configuration	Balanced accuracy	PEHE
VAE	ExtraTrees	Two	***0.634868***	***0.675041***
VAE	Random forest	Two	0.632986	0.675653
PCA	Random forest	Two	0.629856	0.677745
AE	ExtraTrees	Two	0.629503	0.680522
PCA	ExtraTrees	Two	0.628197	0.683752
AE	Random forest	Two	0.626798	0.680120
VAE	Logistic regression	Two	0.626791	0.705085
AE	Logistic regression	Two	0.626455	0.709188
VAE	ExtraTrees	One	0.621575	0.698383
PCA	ExtraTrees	One	0.616874	0.697986
AE	ExtraTrees	One	0.616618	0.699988
VAE	XGBoost	Two	0.615532	0.716068
PCA	XGBoost	Two	0.613898	0.716403
AE	XGBoost	Two	0.612566	0.718603
NMF	ExtraTrees	Two	0.607165	0.705433
PCA	Logistic regression	Two	0.606519	0.723128
NMF	ExtraTrees	One	0.605393	0.708592
NMF	Random forest	Two	0.597810	0.704014
NMF	Random forest	One	0.583920	0.727636
VAE	Random forest	One	0.583522	0.731577
AE	Random forest	One	0.582877	0.731212
PCA	Random forest	One	0.581141	0.732307
AE	XGBoost	One	0.571251	0.715186
VAE	XGBoost	One	0.570046	0.716167
PCA	XGBoost	One	0.566437	0.716168
NMF	XGBoost	Two	0.565989	0.760494
VAE	Gaussian process	One	0.560465	0.759893
VAE	Gaussian process	Two	0.559801	0.758888
NMF	XGBoost	One	0.558269	0.721118
NMF	Gaussian process	Two	0.543848	0.714962
NMF	Gaussian process	One	0.538039	0.717106
NMF	Logistic regression	Two	0.537959	0.713148
PCA	Gaussian process	Two	0.533801	0.755991
AE	Gaussian process	One	0.532606	0.760836
PCA	Gaussian process	One	0.532467	0.757201
AE	Gaussian process	Two	0.532157	0.759965
PCA	Logistic regression	One	0.484090	0.730363
VAE	Logistic regression	One	0.483963	0.730514
AE	Logistic regression	One	0.483900	0.731088
NMF	Logistic regression	One	0.482509	0.731510

Supplementary Figure 79: Optimal model for lesion representations & unobservable bias conditions by balanced accuracy: VAE, two-model ExtraTrees

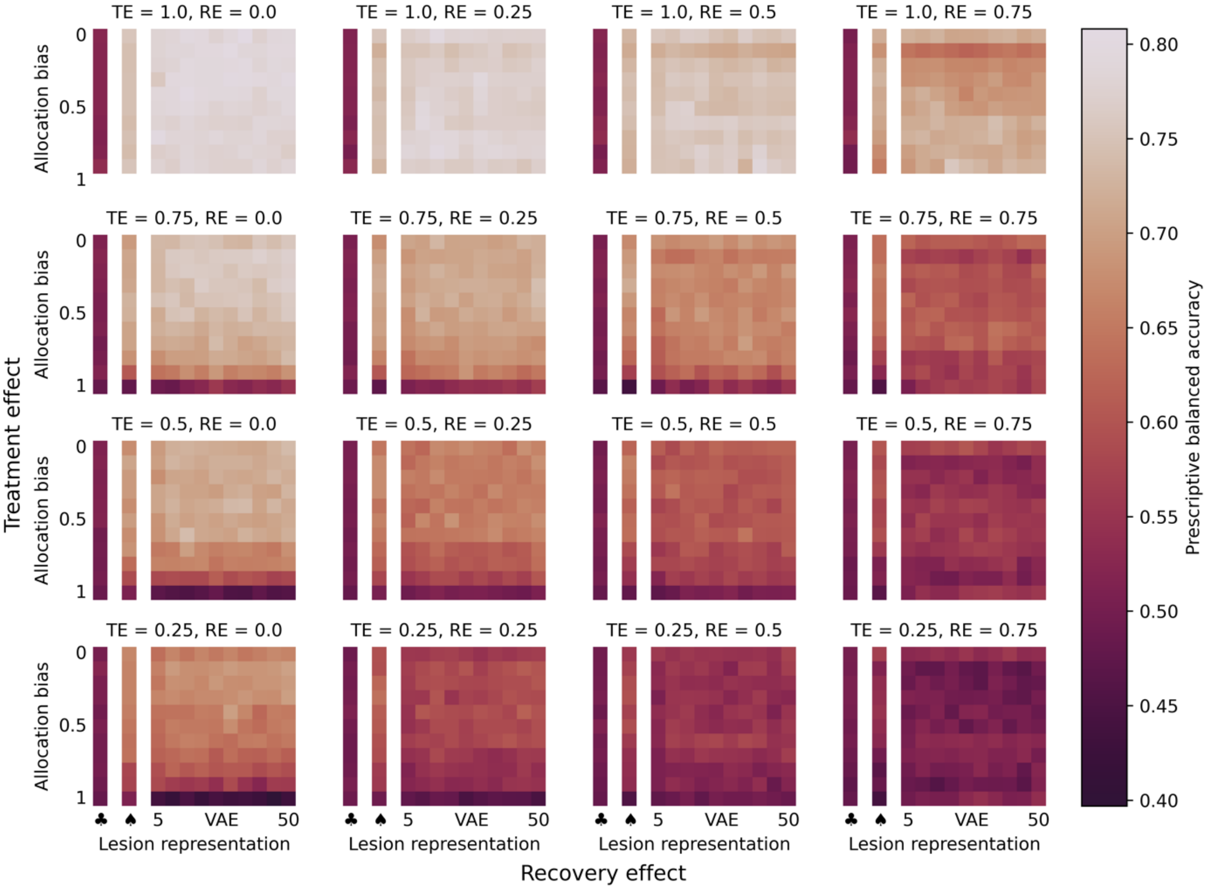

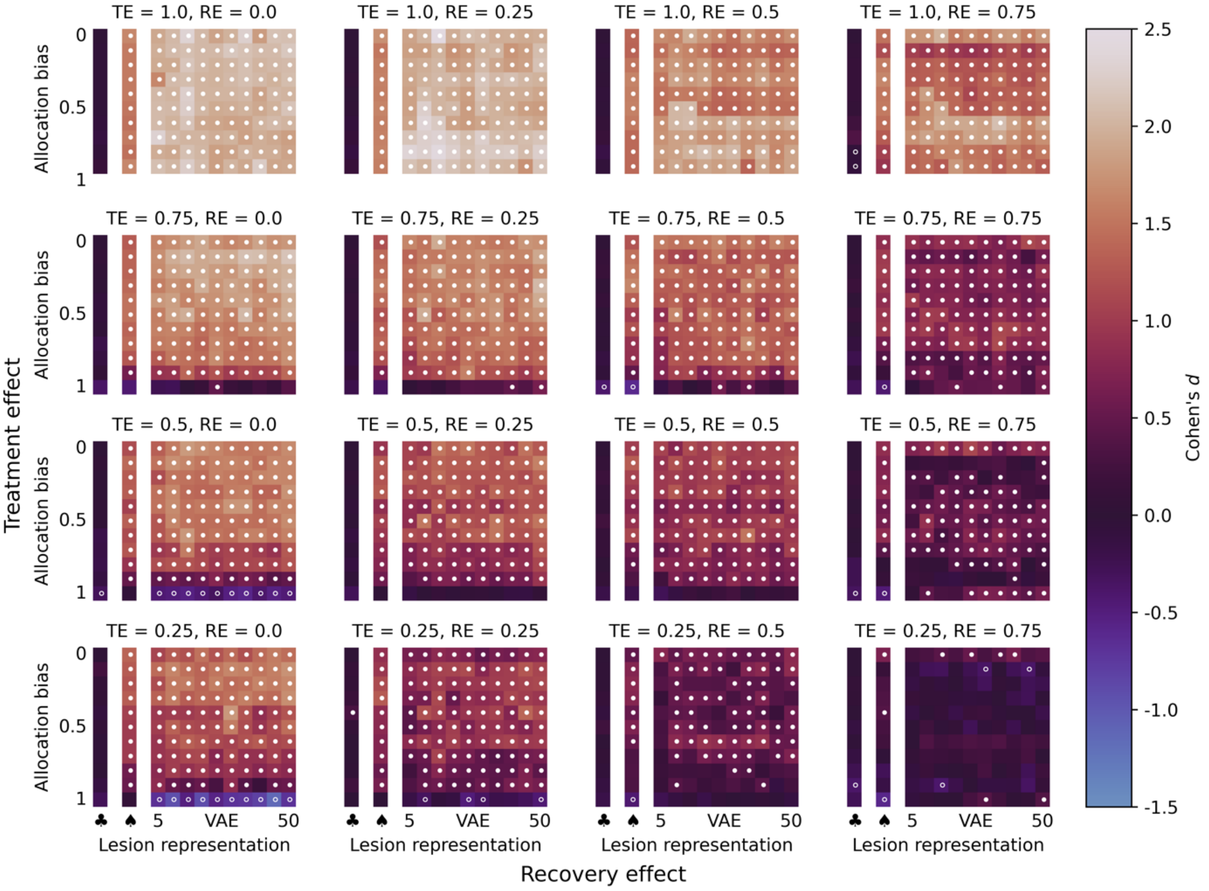

Supplementary Figure 79. Performance comparison of models with lesion representations: balanced accuracy (upper panel) and statistical analysis (lower panel). Prescriptive performance of an optimized, extremely randomized trees-based treatment recommendation system, given binary lesion representations, across the range of response noise (major axes) and allocation bias (minor y-axes), at various levels of expressivity of the individualized representation quantified by embedding length (minor x-axes). The columns to the left of each minor axis show prescriptive performance when individual phenotypes are represented by classification of major affected arterial territory: anterior or posterior circulation (♣) and ACA/MCA/posterior cerebral artery/VB (♠). The upper left subplot shows performance under zero response noise and the lower right subplot shows performance under the conditions of extremely high response noise. The top row of each subplot shows perfect randomization, with zero allocation bias. The upper panel shows prescriptive performance measured by balanced accuracy—the mean proportion of patients allocated to the treatment for which they are truly responsive—for patients that are exclusively responsive to one treatment.

The lower panel shows Cohen's d-effect sizes, by colour, when comparing the prescriptive performance (measured by balanced accuracy, as shown in the upper panel) against a randomized trial based upon individualized information consisting of the major vascular supply (anterior or posterior circulation), at each respective TE/RE pair. Warm colours show performance greater than the comparative randomized trial, while cool colours show the converse. White shows equivalent performance. Trials marked with a circle indicate an effect size exceeding the critical p-value 0.0391 (as adjusted for multiple comparisons at the 0.05 significance level, according to the Benjamini–Hochberg procedure), corresponding to the t-value in a two-sided independent sample t-test. Filled circles show superiority of the prescriptive model from observational data; unfilled circles show superiority of the randomized trial.

TE: treatment effect; RE: recovery effect; VAE: variational auto-encoder representation; ACA: anterior cerebral artery; MCA: middle cerebral artery; VB: vertebrobasilar artery

Supplementary Figure 80: Optimal model for lesion representations & unobservable bias conditions by PEHE: VAE, two-model ExtraTrees

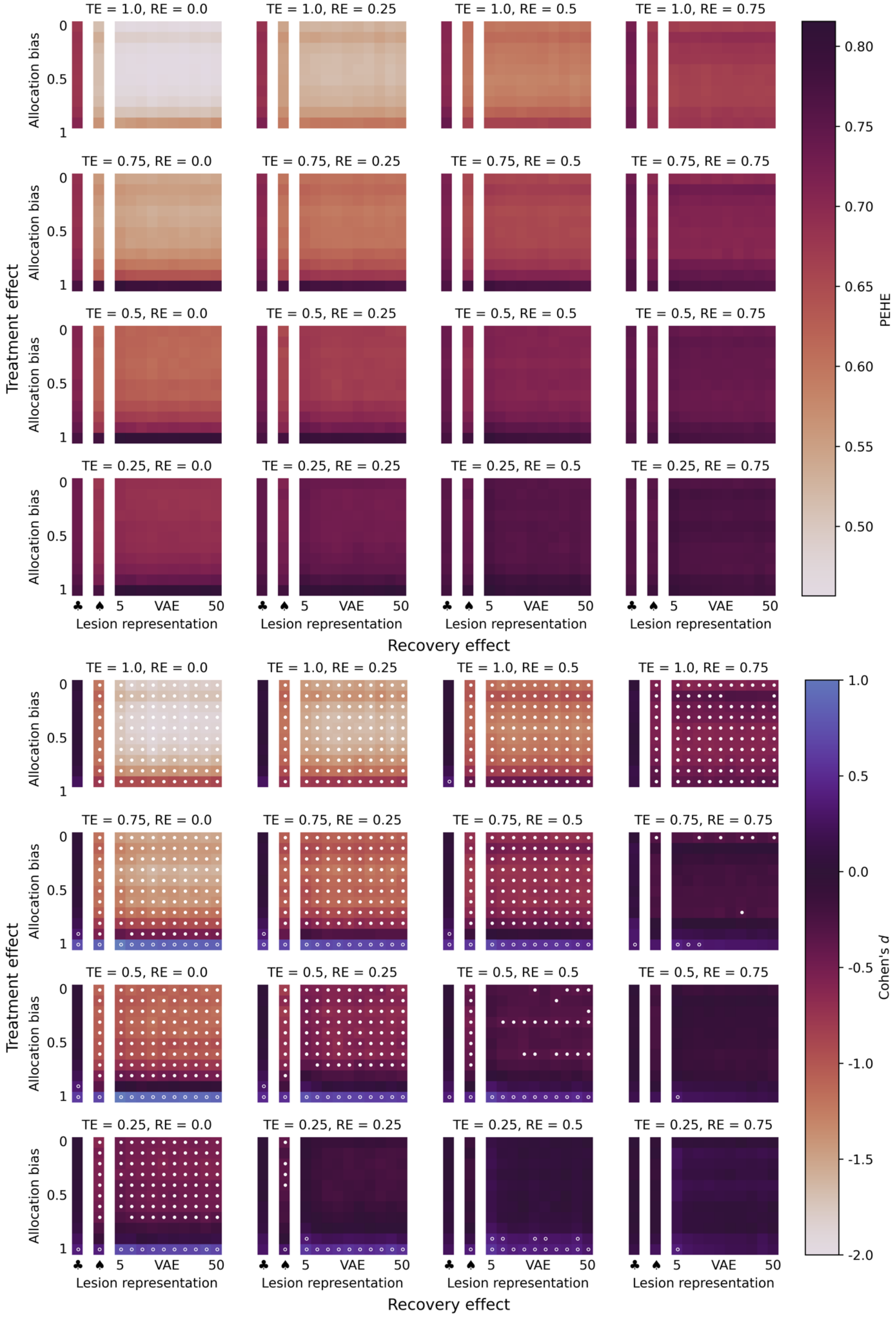

Supplementary Figure 80. Performance comparison of models with lesion representations: PEHE (upper panel) and statistical analysis (lower panel). Prescriptive performance of an optimized, extremely randomized trees-based treatment recommendation system, given binary lesion representations, across the range of response noise (major axes) and allocation bias (minor y-axes), at various levels of expressivity of the individualized representation quantified by embedding length (minor x-axes). The columns to the left of each minor axis show prescriptive performance when individual phenotypes are represented by classification of major affected arterial territory: anterior or posterior circulation (♣) and ACA/MCA/posterior cerebral artery/VB (♠). The upper left subplot shows performance under zero response noise and the lower right subplot shows performance under the conditions of extremely high response noise. The top row of each subplot shows perfect randomization, with zero allocation bias. The upper panel shows prescriptive performance measured by PEHE the root-mean-squared-error in estimation of the true individualized treatment effect (lower is better).

The lower panel shows Cohen's d-effect sizes, by colour, when comparing the prescriptive performance (measured by PEHE, as shown in the upper panel) against a randomized trial based upon individualized information consisting of the major vascular supply (anterior or posterior circulation), at each respective TE/RE pair. Warm colours show performance greater than the comparative randomized trial, while cool colours show the converse. White shows equivalent performance. Trials marked with a circle indicate an effect size exceeding the critical p-value 0.0280 (as adjusted for multiple comparisons at the 0.05 significance level, according to the Benjamini–Hochberg procedure), corresponding to the t-value in a two-sided independent sample t-test. Filled circles show superiority of the prescriptive model from observational data; unfilled circles show superiority of the randomized trial.

TE: treatment effect; RE: recovery effect; VAE: variational auto-encoder representation; ACA: anterior cerebral artery; MCA: middle cerebral artery; VB: vertebrobasilar artery

Supplementary Table 7: Full results table for disconnectome representations & unobservable bias conditions

Representation	Model	Configuration	Balanced accuracy	PEHE
AE	ExtraTrees	Two	***0.718837***	0.579313
AE	Random forest	Two	0.714471	0.578065
VAE	ExtraTrees	Two	0.712073	0.582655
PCA	ExtraTrees	Two	0.710911	0.584353
VAE	Random forest	Two	0.708585	0.581181
AE	ExtraTrees	One	0.708433	0.596302
AE	Logistic regression	Two	0.707938	***0.569637***
VAE	Logistic regression	Two	0.703190	0.572824
VAE	ExtraTrees	One	0.703130	0.598384
PCA	Random forest	Two	0.702288	0.583597
PCA	ExtraTrees	One	0.699538	0.599767
NMF	Gaussian process	Two	0.699174	0.619340
AE	XGBoost	Two	0.697737	0.618557
NMF	Gaussian process	One	0.695420	0.620670
PCA	Logistic regression	Two	0.693443	0.585198
VAE	XGBoost	Two	0.693418	0.622570
PCA	XGBoost	Two	0.690214	0.621575
NMF	ExtraTrees	Two	0.680675	0.601148
AE	XGBoost	One	0.675524	0.604095
VAE	XGBoost	One	0.672516	0.606058
NMF	Logistic regression	Two	0.671997	0.589040
NMF	ExtraTrees	One	0.671001	0.615725
AE	Random forest	One	0.670605	0.629387
VAE	Random forest	One	0.666972	0.630420
PCA	XGBoost	One	0.661126	0.608323
PCA	Random forest	One	0.655172	0.632082
NMF	Random forest	Two	0.650144	0.623045
NMF	XGBoost	One	0.648049	0.614402
NMF	XGBoost	Two	0.647411	0.651443
AE	Gaussian process	One	0.643552	0.678775
AE	Gaussian process	Two	0.642924	0.677752
VAE	Gaussian process	Two	0.631617	0.675007
VAE	Gaussian process	One	0.631354	0.676044
NMF	Random forest	One	0.621269	0.646555
PCA	Gaussian process	Two	0.516954	0.683877
PCA	Gaussian process	One	0.516913	0.684464
PCA	Logistic regression	One	0.500040	0.638890
AE	Logistic regression	One	0.500000	0.639670
NMF	Logistic regression	One	0.500000	0.642166
VAE	Logistic regression	One	0.500000	0.640058

Supplementary Figure 81: Optimal model for disconnectome representations & unobservable bias conditions by balanced accuracy: AE, two-model ExtraTrees

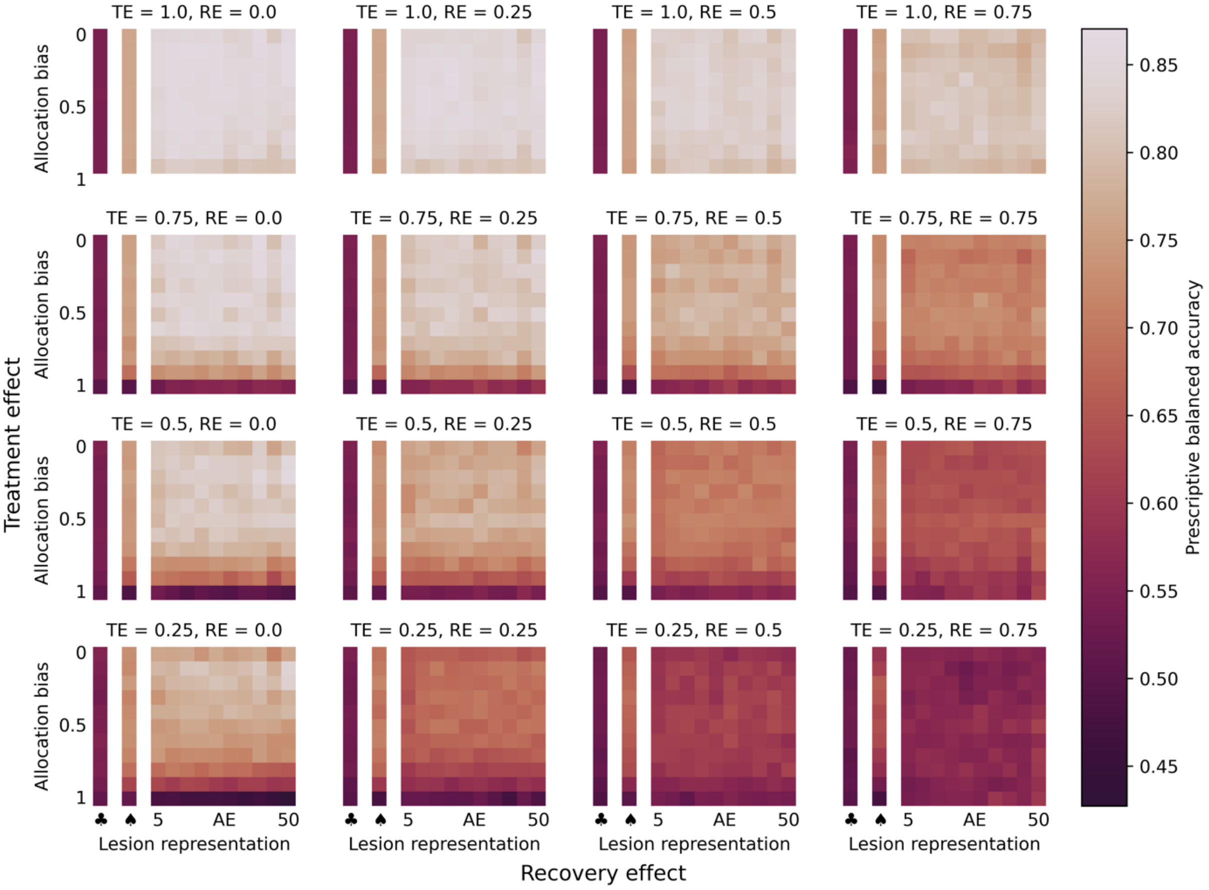

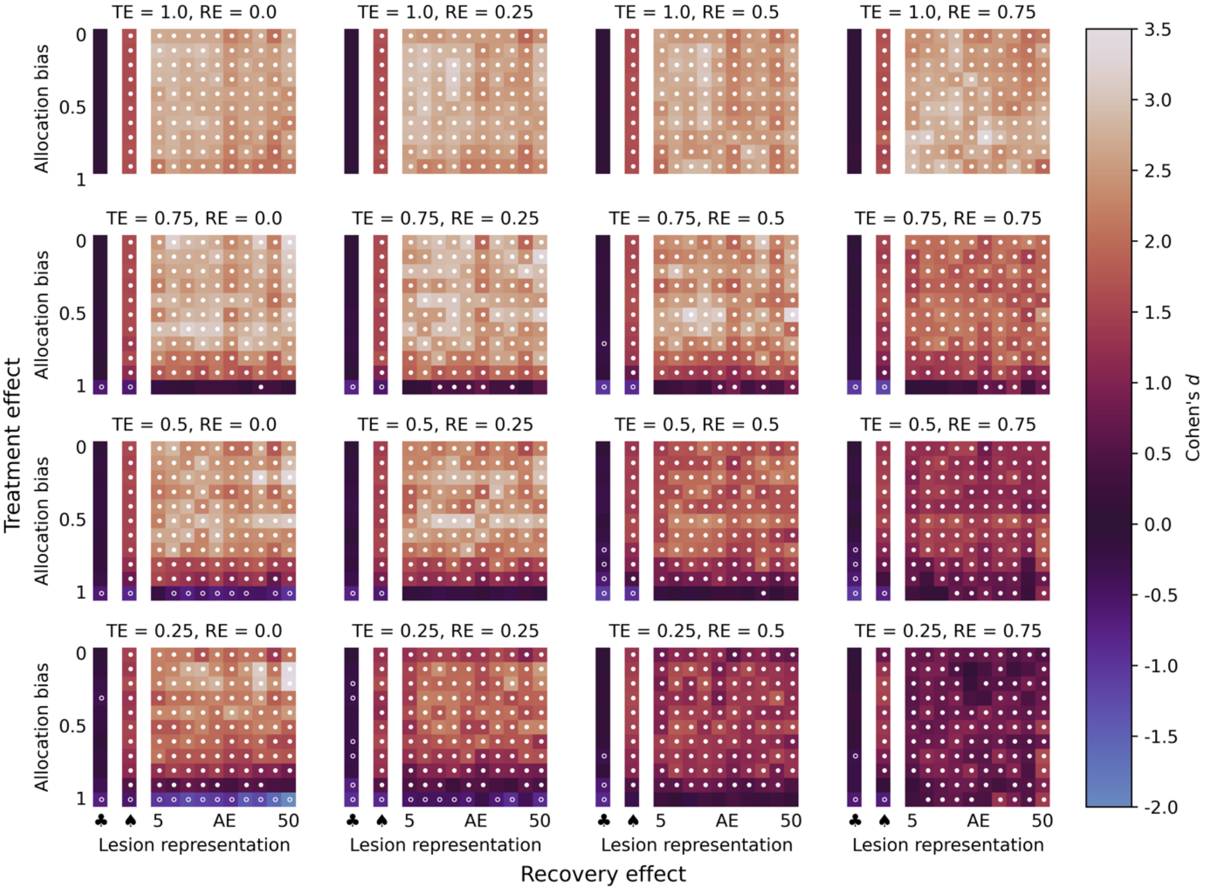

Supplementary Figure 81. Performance comparison of models with disconnectome representations: balanced accuracy (upper panel) and statistical analysis (lower panel). Prescriptive performance of an optimized, extremely randomized trees-based treatment recommendation system, given lesion disconnectome representations, across the range of response noise (major axes) and allocation bias (minor y-axes), at various levels of expressivity of the individualized representation quantified by embedding length (minor x-axes). The columns to the left of each minor axis show prescriptive performance when individual phenotypes are represented by classification of major affected arterial territory: anterior or posterior circulation (♣) and ACA/MCA/posterior cerebral artery/VB (♠). The upper left subplot shows performance under zero response noise and the lower right subplot shows performance under the conditions of extremely high response noise. The top row of each subplot shows perfect randomization, with zero allocation bias. The upper panel shows prescriptive performance measured by balanced accuracy—the mean proportion of patients allocated to the treatment for which they are truly responsive—for patients that are exclusively responsive to one treatment.

The lower panel shows Cohen's d-effect sizes, by colour, when comparing the prescriptive performance (measured by balanced accuracy, as shown in the upper panel) against a randomized trial based upon individualized information consisting of the major vascular supply (anterior or posterior circulation), at each respective TE/RE pair. Warm colours show performance greater than the comparative randomized trial, while cool colours show the converse. White shows equivalent performance. Trials marked with a circle indicate an effect size exceeding the critical p-value 0.0429 (as adjusted for multiple comparisons at the 0.05 significance level, according to the Benjamini–Hochberg procedure), corresponding to the t-value in a two-sided independent sample t-test. Filled circles show superiority of the prescriptive model from observational data; unfilled circles show superiority of the randomized trial.

TE: treatment effect; RE: recovery effect; AE: auto-encoder representation; ACA: anterior cerebral artery; MCA: middle cerebral artery; VB: vertebrobasilar artery

Supplementary Figure 82: Optimal model for disconnectome representations & unobservable bias conditions by PEHE: AE, two-model logistic regression

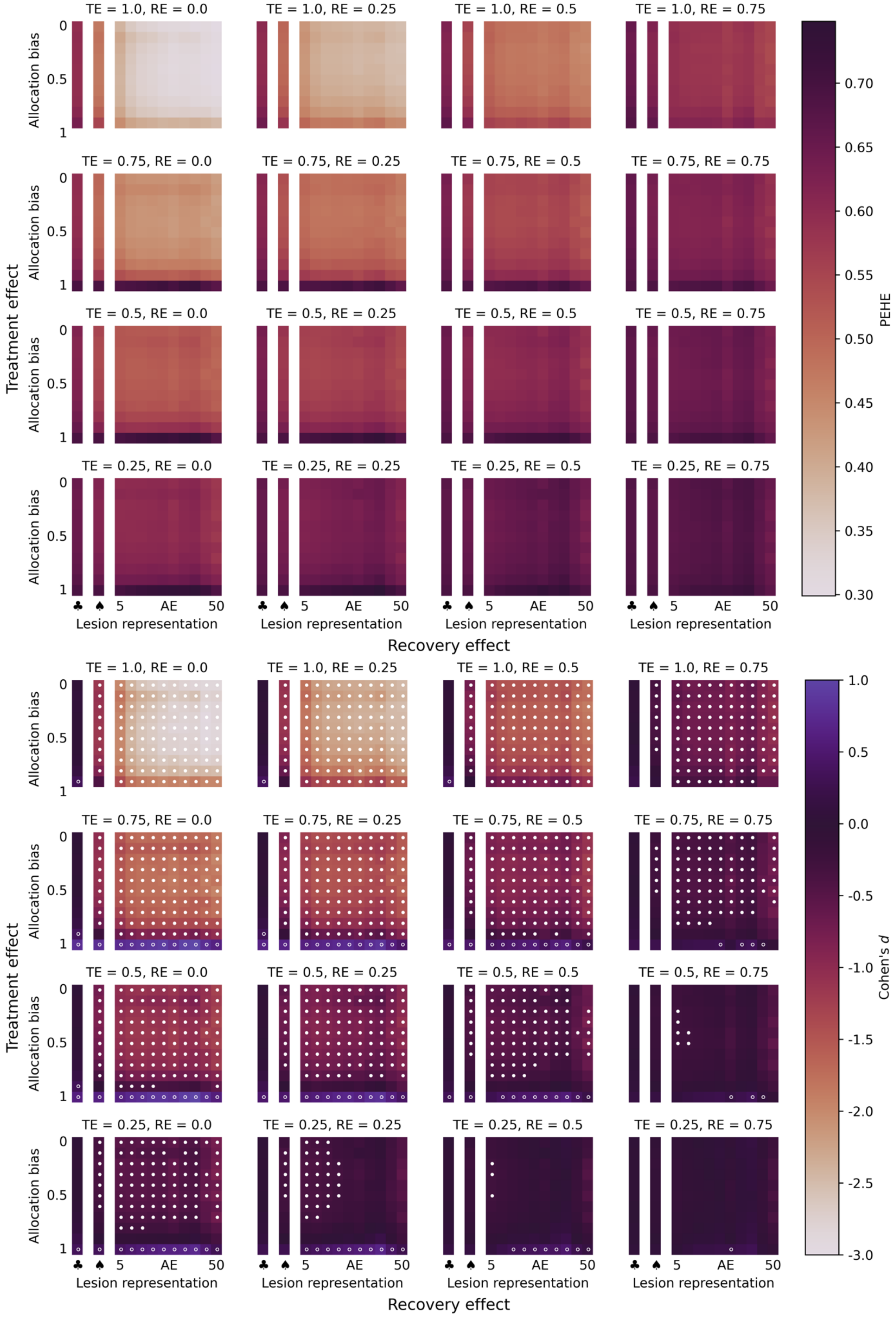

Supplementary Figure 82. Performance comparison of models with disconnectome representations: PEHE (upper panel) and statistical analysis (lower panel). Prescriptive performance of an optimized, logistic regression-based treatment recommendation system, given lesion disconnectome representations, across the range of response noise (major axes) and allocation bias (minor y-axes), at various levels of expressivity of the individualized representation quantified by embedding length (minor x-axes). The columns to the left of each minor axis show prescriptive performance when individual phenotypes are represented by classification of major affected arterial territory: anterior or posterior circulation (♣) and ACA/MCA/posterior cerebral artery/VB (♠). The upper left subplot shows performance under zero response noise and the lower right subplot shows performance under the conditions of extremely high response noise. The top row of each subplot shows perfect randomization, with zero allocation bias. The upper panel shows prescriptive performance measured by PEHE the root-mean-squared-error in estimation of the true individualized treatment effect (lower is better).

The lower panel shows Cohen's d-effect sizes, by colour, when comparing the prescriptive performance (measured by PEHE, as shown in the upper panel) against a randomized trial based upon individualized information consisting of the major vascular supply (anterior or posterior circulation), at each respective TE/RE pair. Warm colours show performance greater than the comparative randomized trial, while cool colours show the converse. White shows equivalent performance. Trials marked with a circle indicate an effect size exceeding the critical p-value 0.0327 (as adjusted for multiple comparisons at the 0.05 significance level, according to the Benjamini–Hochberg procedure), corresponding to the t-value in a two-sided independent sample t-test. Filled circles show superiority of the prescriptive model from observational data; unfilled circles show superiority of the randomized trial.

TE: treatment effect; RE: recovery effect; AE: auto-encoder representation; ACA: anterior cerebral artery; MCA: middle cerebral artery; VB: vertebrobasilar artery

Supplementary Figure 83: Results summary by PEHE

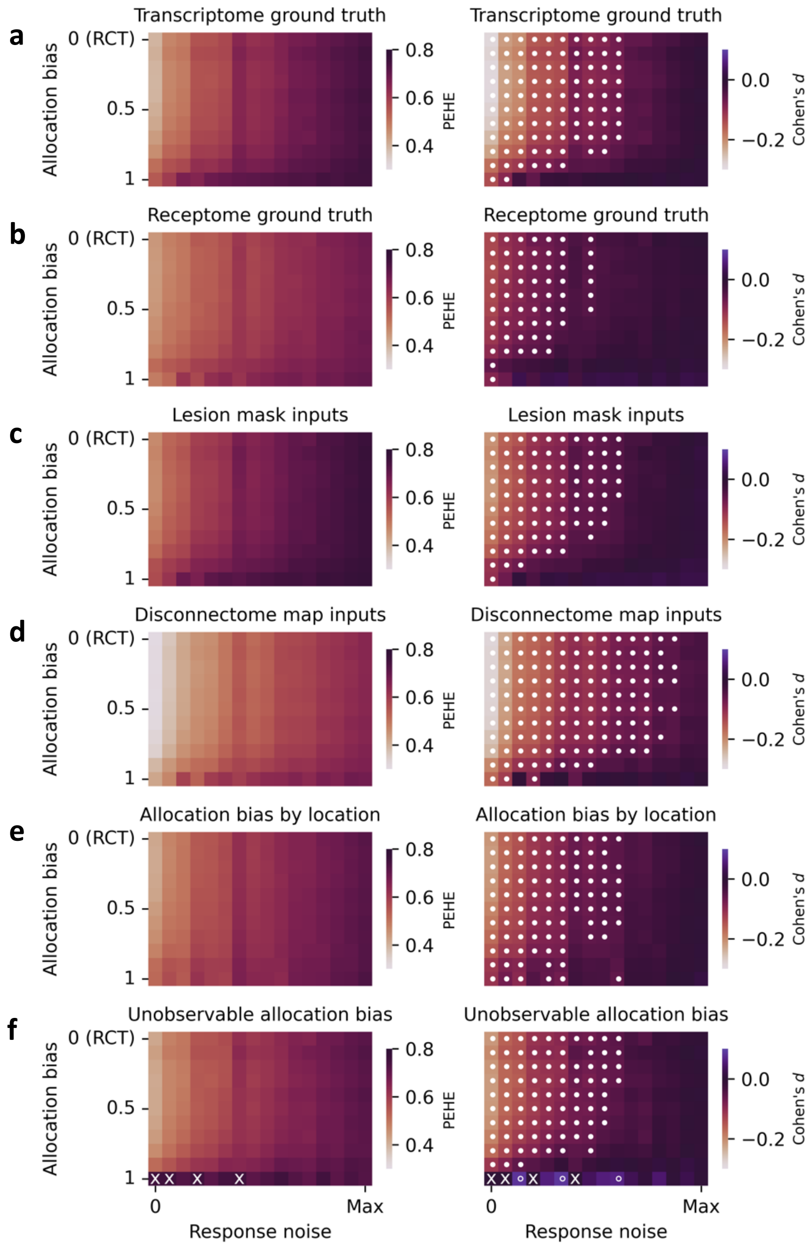

Supplementary Figure 83. Prescriptive performance with the optimal richly expressive representation quantified by PEHE (left panel column, see Supplementary Tables 4-7; Supplementary Figures 75-82 for full descriptions), and two-sided independent sample *t*-test statistical comparisons against a simple vascular baseline (anterior or posterior vascular territories) (right panel column). A filled circle indicates significantly higher performance for the expressive representation, and an unfilled circle significantly higher performance for the baseline model (using a Benjamini–Hochberg corrected critical value for 0.05 significance level). An 'x' indicates simulation conditions with insufficient class balance to permit prescriptive model fitting (e.g. because there are no non-responders). Rows **a** and **b** show performance averaged across the criteria for treatment responsiveness (transcriptome or receptome); rows **c** and **d** across lesion input representation type (lesion masks or disconnectomes); and rows **e** and **f** across allocation bias observability (location-based or unobservable). The optimal expressive representation is shown to be non-inferior to simple vascular territories at informing prescription across almost the full landscape of observational conditions, and superior in the majority. See Figure 6 for respective balanced accuracy plots.

Supplementary Figure 84: Results summary, stratified by deficit, by PEHE

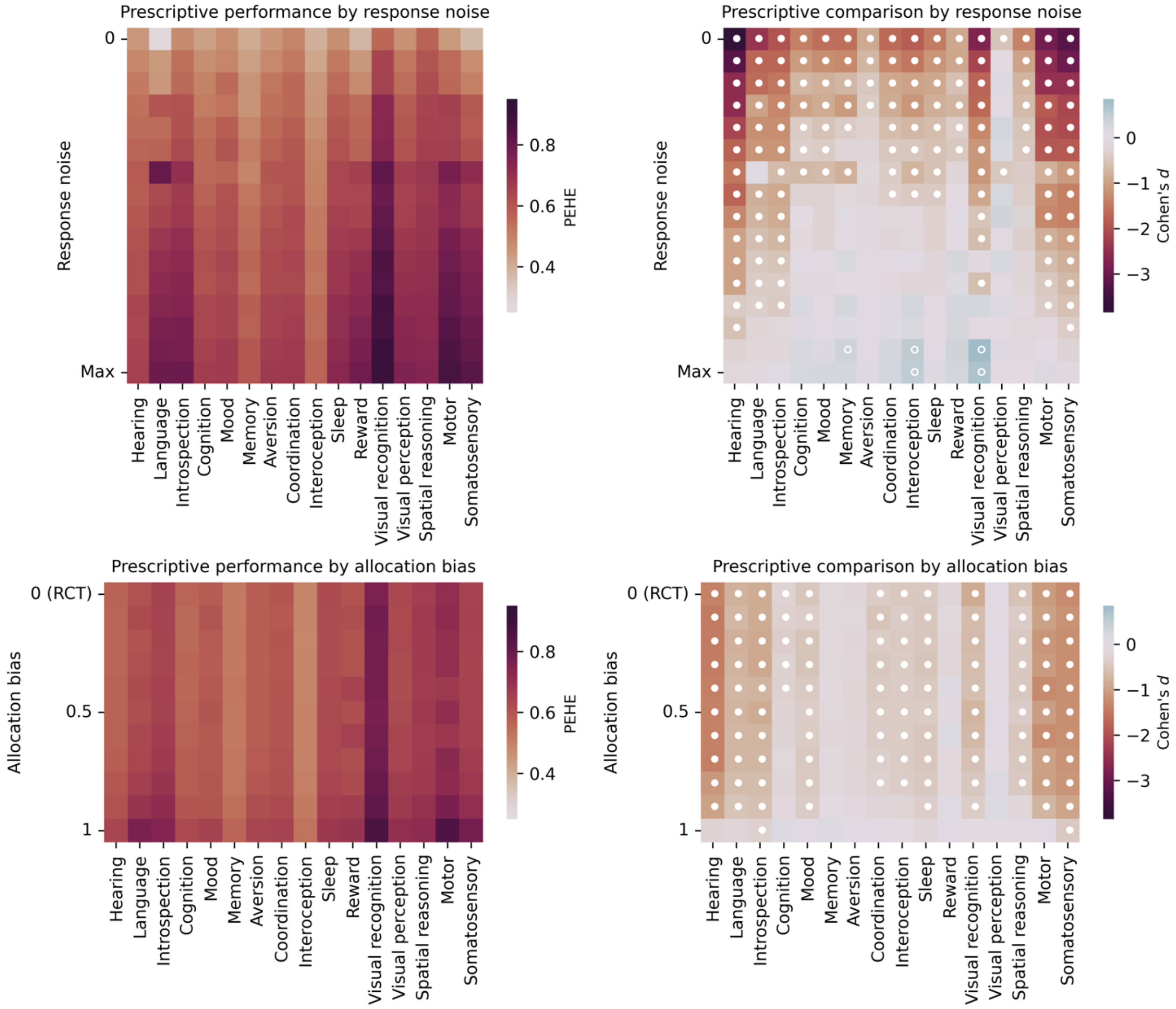

Supplementary Figure 84. Prescriptive performance with the optimal richly expressive representation quantified by PEHE (see Supplementary Tables 4-7; Supplementary Figures 75-82 for full description), stratified according to the modelled functional deficit, and averaged across all simulation conditions. The right column shows Cohen's d-effect size when comparing prescriptive performance using the optimal representation against a simple vascular baseline. A filled circle indicates superior performance for the optimal representation, according to two-sided independent sample t-tests, beyond the Benjamini–Hochberg corrected critical value for 0.05 significance level; an unfilled circle indicates superior performance for the simple vascular baseline correspondingly. The full anatomical—physiological modelling framework is visualized in Supplementary Figures 11-42 for receptome and Supplementary Figures 43-74 for transcriptome. The advantage in prescriptive performance for the richly expressive approach is shown to generalize across modelled functional deficits as well as data conditions defined by outcome response noise and treatment allocation bias. See Figure 7 for respective balanced accuracy plot.

Supplementary Table 8: Performance comparison under ideal experimental conditions

a. Balanced accuracy; lesion representation; location bias; two-sided independent sample *t*-test

Representation	Embedding length	Prescriptive model	Inferential configuration	Performance (95% CI)	Control (95% CI)	t	p	d
VAE	30	ExtraTrees	Two-model	0.778 (0.74, 0.84)	0.521 (0.48, 0.56)	5.78	0.0000038	1.76

b. Balanced accuracy; lesion representation; unobservable bias; two-sided independent sample *t*-test

Representation	Embedding length	Prescriptive model	Inferential configuratio n	Performance (95% CI)	Control (95% CI)	t	p	d
AE	25	ExtraTrees	Two-model	0.773 (0.71, 0.84)	0.524 (0.48, 0.57)	5.98	0.0000019	1.70

c. Balanced accuracy; disconnectome representation; location bias; two-sided independent sample *t*-test

Representation	Embedding length	Prescriptive model	Inferential configuration	Performance (95% CI)	Control (95% CI)	t	p	d
AE	50	ExtraTrees	Two-model	0.873 (0.80, 0.94)	0.546 (0.51, 0.58)	8.85	0.000000024	2.63

d. Balanced accuracy; disconnectome representation; unobservable bias; two-sided independent sample *t*-test

Representation	Embedding length	Prescriptive model	Inferential configuration	Performance (95% CI)	Control (95% CI)	t	p	d
AE	50	ExtraTrees	Two-model	0.862 (0.79, 0.94)	0.546 (0.51, 0.58)	8.29	0.000000098	2.52

e. PEHE; lesion representation; location bias; two-sided independent sample *t*-test

Representation	Embedding length	Prescriptive model	Inferential configuration	Performance (95% CI)	Control (95% CI)	t	p	d
AE	25	ExtraTrees	Two-model	0.465 (0.42, 0.50)	0.676 (0.61, 0.74)	-5.53	0.0000058	-1.44

f. PEHE; lesion representation; unobservable bias; two-sided independent sample *t*-test

Representation	Embedding length	Prescriptive model	Inferential configuration	Performance (95% CI)	Control (95% CI)	t	p	d
Lateralized arterial territory atlas	N/A	Random forest	Two-model	0.516 (0.46, 0.57)	0.676 (0.62, 0.73)	-3.71	0.00083	-0.957

g. PEHE; disconnectome representation; location bias; two-sided independent sample *t*-test

Representation	Embedding length	Prescriptive model	Inferential configuration	Performance (95% CI)	Control (95% CI)	t	p	d
VAE	50	Logistic regression	Two-model	0.305 (0.26, 0.34)	0.592 (0.54, 0.64)	-6.04	0.0000066	-2.27

h. PEHE; disconnectome representation; unobservable bias; two-sided independent sample *t*-test

Representation	Embedding length	Prescriptive model	Inferential configuration	Performance (95% CI)	Control (95% CI)	t	p	d
VAE	50	Logistic regression	Two-model	0.304 (0.27, 0.34)	0.592 (0.54, 0.64)	-6.33	0.0000028	-2.34

Supplementary Figure 85: Glossary

Average treatment effect, ATE: $\mathbb{E}\left[Y_i^{(\mathrm{A})} - Y_i^{(\mathrm{B})}\right]$. The expected difference between two potential outcomes. Sometimes referred to as the population average treatment effect, or PATE.

Conditional average treatment effect, CATE: $\hat{\tau}(\mathbf{x}_i) := \mathbb{E}[Y_i^{(\mathrm{A})} - Y_i^{(\mathrm{B})} \mid \mathbf{X} = \mathbf{x}_i]$. The expected difference between potential outcomes for individual i, conditioned on phenotype characterization, $\mathbf{x}_i$.

Confounding hyperparameter: a hyperparameter that controls the association between treatment allocation and outcome when generating virtual trial data.

Identifiability: a causal quantity such as the ATE, or the CATE, is *identifiable* if it can be estimated, typically given a set of assumptions.

Individualized treatment effect, ITE: $\tau_i := Y_i^{(\mathrm{A})} - Y_i^{(\mathrm{B})}$. The difference between the two potential outcomes for individual i.

Prescriptive inference: inferring how to prescribe a treatment from predictive models of potential outcomes.

Treatment responsiveness: $\mathbb{P}(Y = 1 \mid W = w,\ \boldsymbol{X} = \mathbf{x})$. The probability of a favourable outcome for an individual, with phenotype characterization $\mathbf{x}$, given treatment w.

Virtual trial: a set of semi-synthetic observational data, $\{(\mathbf{x}_i, w_i, y_i)\}_{i=1}^{n}$, with a framework for deciding treatment allocation and responsiveness, CATE estimation, inferring prescriptions and then evaluating this inference. The phenotype characterizations are representations of real ischaemic stroke lesions. The allocation and responsiveness framework is designed to promote biological plausibility, using functional, transcriptomic and receptomic data. Framework hyperparameters control confounding and response noise.
