# Supplementary material for: Individualized prescriptive inference in ischaemic stroke
Source: Nat Commun. 2025 Oct 16;16:8968. doi: 10.1038/s41467-025-64593-7 (PMC12531334; doi:10.1038/s41467-025-64593-7)
Supplement: Supplementary file 3 — Reporting summary [file 41467_2025_64593_MOESM3_ESM.pdf]

nature portfolio

Corresponding author(s): Dominic Giles and Parashkev Nachev

Last updated by author(s): Jun 30, 2025

Reporting Summary

Nature Portfolio wishes to improve the reproducibility of the work that we publish. This form provides structure for consistency and transparency in reporting. For further information on Nature Portfolio policies, see our Editorial Policies and the Editorial Policy Checklist.

Statistics

For all statistical analyses, confirm that the following items are present in the figure legend, table legend, main text, or Methods section.

n/a	Confirmed	
☐	☒	The exact sample size (*n*) for each experimental group/condition, given as a discrete number and unit of measurement
☐	☒	A statement on whether measurements were taken from distinct samples or whether the same sample was measured repeatedly
☐	☒	The statistical test(s) used AND whether they are one- or two-sided<br>*Only common tests should be described solely by name; describe more complex techniques in the Methods section.*
☐	☒	A description of all covariates tested
☐	☒	A description of any assumptions or corrections, such as tests of normality and adjustment for multiple comparisons
☐	☒	A full description of the statistical parameters including central tendency (e.g. means) or other basic estimates (e.g. regression coefficient) AND variation (e.g. standard deviation) or associated estimates of uncertainty (e.g. confidence intervals)
☐	☒	For null hypothesis testing, the test statistic (e.g. *F*, *t*, *r*) with confidence intervals, effect sizes, degrees of freedom and *P* value noted<br>*Give P values as exact values whenever suitable.*
☒	☐	For Bayesian analysis, information on the choice of priors and Markov chain Monte Carlo settings
☒	☐	For hierarchical and complex designs, identification of the appropriate level for tests and full reporting of outcomes
☐	☒	Estimates of effect sizes (e.g. Cohen's *d*, Pearson's *r*), indicating how they were calculated

Our web collection on statistics for biologists contains articles on many of the points above.

Software and code

Policy information about availability of computer code

Data collection	Volumetric magnetic resonance data were acquired from patients with confirmed acute ischaemic stroke diagnoses at University College London NHS Foundation Trust from 2005 to 2020, culminating in a dataset describing 4119 ischaemic events.
Data analysis	Python 3.10 was used for all analyses, with open source packages: matplotlib 3.6.0, nibabel 4.0.2, nilearn 0.9.2, nimfa 1.4.0, numpy 1.23.4, pandas 1.5.1, Pillow 9.2.0, scipy 1.9.2, seaborn 0.12.1, scikit-learn 1.1.3 and pytorch 1.13 were all used.<br><br>In addition, GUI-controlled visualisation software used includes ITK-SNAP 3.6.0, MRIcroGL 1.2, ParaView 5.9.0 and Surf Ice 1.0.<br><br>The custom software repository that was developed using the above libraries for this project will be available on request.

For manuscripts utilizing custom algorithms or software that are central to the research but not yet described in published literature, software must be made available to editors and reviewers. We strongly encourage code deposition in a community repository (e.g. GitHub). See the Nature Portfolio guidelines for submitting code & software for further information.

Data

Policy information about availability of data

All manuscripts must include a data availability statement. This statement should provide the following information, where applicable:

- Accession codes, unique identifiers, or web links for publicly available datasets
- A description of any restrictions on data availability
- For clinical datasets or third party data, please ensure that the statement adheres to our policy

Functional grey matter parcellations including their subdivided versions will be made available after publication. Neuroimaging data cannot be released publicly owing to the terms of access to clinical data specified in the ethical approval.

Research involving human participants, their data, or biological material

Policy information about studies with human participants or human data. See also policy information about sex, gender (identity/presentation), and sexual orientation and race, ethnicity and racism.

Reporting on sex and gender	Data concerning patient sex was available for 3478/4119 events, with 1960 recorded as male (56.4%) and 1518 recorded as female (43.6%). This information was acquired from the clinical data, and therefore subject to the clinical protocol for recording patient sex and gender.
Reporting on race, ethnicity, or other socially relevant groupings	Data was collected in an unselected manner, with ischaemic stroke diagnosis as the only inclusion criteria. The data should therefore be representative of the diverse stroke patient population presenting to a central London hospital.
Population characteristics	This clinically-derived sample is representative of the population of ischaemic stroke patients in London, UK, as the sole inclusion criteria. This dataset is an expands upon data used in previously published work (e.g. Xu et al. (2018); Bonkhoff et al. (2021)), that now comprises 4119 events and is the largest dataset of its kind to our knowledge.<br><br>Data concerning patient age was available for 3487/4119 events, with mean 67.042 years and standard deviation 15.46 years.<br><br>Data concerning patient sex was available for 3478/4119 events, with 1960 recorded as male (56.4%) and 1518 recorded as female (43.6%)<br><br>The lesion volume distribution is visualised in Supplementary Figure S7 and histogram of patient age in S8.
Recruitment	Recruitment was guided by neuro-radiological reporting of patients undergoing imaging in response to stroke-suggestive symptoms, recruited upon clinical confirmation of acute ischaemic stroke diagnosis.
Ethics oversight	This study was performed under ethical approval by the West London & GTAC research ethics committee for consentless use of fully anonymized data. The data is an unselected sample based on clinical diagnosis of acute ischaemic stroke; no other specific inclusion or exclusion criteria were used. It should therefore be proportionally representative of the acute ischaemic stroke patient population presenting to hospital in London, UK.

Note that full information on the approval of the study protocol must also be provided in the manuscript.

Field-specific reporting

Please select the one below that is the best fit for your research. If you are not sure, read the appropriate sections before making your selection.

☒ Life sciences ☐ Behavioural & social sciences ☐ Ecological, evolutionary & environmental sciences

For a reference copy of the document with all sections, see nature.com/documents/nr-reporting-summary-flat.pdf

Life sciences study design

All studies must disclose on these points even when the disclosure is negative.

Sample size	This dataset comprises 4119 ischaemic stroke patient events, the largest of its kind to our knowledge. Interventions and outcomes were synthetic, and repeated virtual trial recruitment was dependent upon intersection with a custom-built functional parcellation, with performance of prescriptive modelling evaluated at all virtual sample sizes.
Data exclusions	Only patients with clinically confirmed acute ischaemic stroke were included.
Replication	The study is replicable through re-execution of code (available on request). Some stochastic methods are used which can lead to variability. The whole analysis (including dimensionality reduction) was repeated 10-fold with separate train/test (90%/10%) divisions, demonstrating reproducible findings.

Randomization	This study involves the simulation of trial data across the range of allocation bias from completely randomised to completely dependent upon patient information, and assesses the ability to select optimal treatments across this range.
Blinding	The true optimal treatments were programmatically blinded for prescriptive model fitting. These models were exposed only to lesion representation, intervention received and outcome observed. The optimal intervention is the target to be inferred and its true value (known due to semi-synthetic frame) is disclosed only at the evaluation stage.

Reporting for specific materials, systems and methods

We require information from authors about some types of materials, experimental systems and methods used in many studies. Here, indicate whether each material, system or method listed is relevant to your study. If you are not sure if a list item applies to your research, read the appropriate section before selecting a response.

Materials & experimental systems

n/a	Involved in the study
☒	☐ Antibodies
☒	☐ Eukaryotic cell lines
☒	☐ Palaeontology and archaeology
☒	☐ Animals and other organisms
☐	☒ Clinical data
☒	☐ Dual use research of concern
☒	☐ Plants

Methods

n/a	Involved in the study
☒	☐ ChIP-seq
☒	☐ Flow cytometry
☐	☒ MRI-based neuroimaging

Clinical data

Policy information about clinical studies

All manuscripts should comply with the ICMJE guidelines for publication of clinical research and a completed CONSORT checklist must be included with all submissions.

Clinical trial registration	This is not a clinical trial.
Study protocol	This is not a clinical trial.
Data collection	Data were collected from all patients with confirmed acute ischaemic stroke that received magnetic resonance imaging at University College London Hospitals NHS Foundation Trust, from 2005 to 2020.
Outcomes	This is a simulation study; no empirical outcomes were collected.This is a simulation study; no empirical outcomes were collected.

Plants

Seed stocks	N/A
Novel plant genotypes	N/A
Authentication	N/A

Magnetic resonance imaging

Experimental design

Design type	This is not a functional imaging experiment but an analysis of structural imaging derived from acute diffusion-weighted imaging in patients with radiologically confirmed ischaemic stroke.
Design specifications	This is not a functional imaging experiment.
Behavioral performance measures	No behavioural data was collected. Lesion-deficit associations were simulated by intersecting each stroke lesion image volume with a custom functional parcellation. The only empirical clinical information used was the diffusion-weighted neuroimaging data.

Acquisition

Imaging type(s)	Diffusion-weighted magnetic resonance imaging data were acquired.
Field strength	All data were acquired in routine clinical settings, using machinery and protocols in place at the time of event. The dates of acquisition range from 2005 to 2020, from scanners manufactured by General Electric, Hitachi, Philips, Siemens, or Toshiba and field strengths of either 1.5T or 3T. Note that the nature and scale of ischaemic lesions makes their spatial parameters relatively invariant to the specifics of the imaging instrument.
Sequence & imaging parameters	A variety of diffusion-weighted echoplanar imaging protocols were used, reflecting clinical practice over the period of data collection. The b0 and b1000 image from each sequence were used to derive intersubject registration and lesion segmentation parameters respectively.
Area of acquisition	Whole brain scans were used.

Diffusion MRI ☒ Used ☐ Not used

Parameters: b-value = 0 and b-value = 1000 were used, the latter representing the geometric mean of constituent directionally-specific b1000 images.

Preprocessing

Preprocessing software	Non-linear registration to MNI152 template space was performed using SPM12. Segmentation was performed in using Python 3, making use of the following packages: bcblib 0.2.11, einops 0.4.1, matplotlib 3.5.1, monai 0.7.0, nibabel >= 3, nilearn 0.9.0, numpy 1.18.5, scipy 1.7.3, torch 1.10.2, torchio 0.18.73 and torchvision 0.11.3.
Normalization	All images were normalised to standard space comprising (x, y, z) = (91, 109, 91) voxels of 2 cubic mm, using non-linear registration computed from the b-value = 0 images and applied to the b-value = 1000 images. Voxel intensity was normalised to between 0 and 1.
Normalization template	Image volumes were normalised into the template space of the MNI152 co-ordinate system.
Noise and artifact removal	Multiple model fitting with multiple metric objectives (Dice coefficient and binary cross-entropy) were used to improve the capacity for generalisation to unseen data. Various augmentation functions were applied to training data for further improvement to model generalisability. These functions were randomly applied in order to artificially increase the size of the dataset by adding noise or bias, shifting the intensity of the voxels, applying slight affine and elastic transformations and inverting the left and right hemispheres.<br><br>33 CoordConv (El Jurdi et al., 2021) channels were added: 3 arrays of equal size to the images containing the increasing x, y and z coordinates, providing the model with voxel locations, with the intention to reduce the risk of false positives due to artefacts.<br><br>Images were manually inspected and corrected if necessary by experts as described in the Methods section.
Volume censoring	As above. Various mechanisms were applied to mitigate the risks of suboptimal preprocessing and segmentation due to artefact and all images underwent a manual inspection process.

Statistical modeling & inference

Model type and settings	Prescriptive inference was evaluated: the machine selection of intervention with the objective to maximise the probability of success for each individual, comparing various representations and inferential configurations within an overarching crossvalidation arrangement. Balanced accuracy (the mean of the proportions of individuals correctly inferred to be susceptible to each intervention, is able to be modelled due to the use of an empirically-driven semi-synthetic ground truth. Evaluation by precision in estimation of treatment effects, PEHE, is also presented in the supplementary material, S11-17.
Effect(s) tested	For comparative performance relative to simulated randomized controlled trials, two-sided paired t-tests were used to obtain t- and p-values, under each of the deficit and ground truth modelling conditions. Further detail is provided in the methods section.

Specify type of analysis: ☐ Whole brain ☐ ROI-based ☒ Both

Anatomical location(s): A custom parcellation for lesion-deficit simulation was established from meta-analytic functional data. A further custom division within this functional parcellation was established for treatment susceptibility inference from large-scale gene expression or receptor distribution data.

Statistic type for inference (See Eklund et al. 2016)	Simulation of deficit was based upon intersection of binary lesion mask or disconnectome distribution with functional grey matter parcellation. Simulation of true optimal treatment was based upon intersection with gene expression or receptor distribution-informed functional subnetworks. Inference of optimal treatment was then performed on semi-synthetic ground truths of patient-representative vector embedding, index of hypothetical intervention received and hypothetical observed outcome. Further details are described in the methods section.
Correction	Benjamini-Hochberg correction for false discovery rate, with alpha = 0.05, was applied.

Models & analysis

n/a	Involved in the study
☐	☒ Functional and/or effective connectivity
☒	☐ Graph analysis
☐	☒ Multivariate modeling or predictive analysis

Functional and/or effective connectivity	Voxel-based agglomerative clustering of Z-valued meta-analytic data from NeuroQuery (Dockès et al., 2020) was performed, leading to a hierarchical functional grey matter parcellation, according to agglomeration thresholding of Euclidean distances of each voxel across their functional distributions.
Multivariate modeling and predictive analysis	Embedding vectors representing each individual were processed from the preprocessed diffusion-weighted image using various methods including deep autoencoders, NMF and PCA. Various machine learning classification models were trained to predict binary outcome, given patient features. An ensemble of these well-fitted models was thereby applied in a prescriptive manner, with the objective of selecting the intervention that has the greatest probability of success, tailored to each individual patient. This system was tested across the ranges of outcome noise and allocation bias, typical of observational data.
